# Validation of MEWS, NEWS, NEWS-2 and qSOFA for different infection foci at the emergency department, the acutelines cohort

**DOI:** 10.1007/s10096-024-04961-1

**Published:** 2024-10-16

**Authors:** Carolina Hincapié-Osorno, Raymond J. van Wijk, Douwe F. Postma, Jacqueline Koeze, Jan C. Ter Maaten, Fabian Jaimes, Hjalmar R. Bouma

**Affiliations:** 1 Department of Internal Medicine, University of Groningen, University Medical Center Groningen, Groningen, The Netherlands; 2 Department of Acute Care, University of Groningen, University Medical Center Groningen, Groningen, The Netherlands; 3 Department of Internal Medicine and Infectious Diseases, University of Groningen, University Medical Center Groningen, Groningen, The Netherlands; 4 Department of Critical Care, University of Groningen, University Medical Center Groningen, Groningen, The Netherlands; 5 Department of Internal Medicine, Universidad de Antioquia, Medellín, Antioquia Colombia; 6 Department of Clinical Pharmacy and Pharmacology, University of Groningen, University Medical Center Groningen, Groningen, The Netherlands

**Keywords:** Sepsis, Early warning score, Mortality, Infections, Prognosis, Intensive care unit

## Abstract

**Purpose:**

Sepsis is a leading cause of morbidity and mortality globally. The lack of specific prognostic markers necessitates tools for early risk identification in patients with suspected infections in emergency department (ED). This study evaluates the prognostic accuracy of various Early Warning Scores (EWS)—MEWS, NEWS, NEWS-2, and qSOFA—for in-hospital mortality, 30-day mortality, and ICU admission, considering the site of infection.

**Methods:**

A retrospective analysis was conducted using data from the Acutelines cohort, which included data collected from patients admitted to the University Medical Centre Groningen ED between September 2020 and July 2023. Patients were included if they had an ICD-10 code for infection. EWS were calculated using clinical data within 8 h post-admission. Predictive performance was assessed using AUC-ROC, calibration by the Hosmer-Lemeshow test and calibration curves, and operative characteristics like sensitivity and specificity.

**Results:**

A total of 1661 patients were analyzed, with infections distributed as follows: lower respiratory tract (32.9%), urinary tract (30.7%), abdominal (12.5%), skin and soft tissue (9.5%), and others (8.2%). The overall in-hospital mortality was 6.7%, and ICU admission was 7.1%. The highest AUC-ROC for in-hospital mortality prediction was observed with NEWS and NEWS-2 in abdominal infections (0.86), while the lowest was for qSOFA in skin and soft tissue infections (0.57). Predictive performance varied by infection site.

**Conclusions:**

The study highlights the variability in EWS performance based on infection site, emphasizing the need to consider the source of infection in EWS development for sepsis prognosis. Tailored or hybrid models may enhance predictive accuracy, balancing simplicity and specificity.

**Supplementary Information:**

The online version contains supplementary material available at 10.1007/s10096-024-04961-1.

## Introduction

Sepsis remains one of the leading causes of morbidity and mortality worldwide, posing significant challenges for healthcare systems. Early identification of patients at high risk for poor outcomes, such as death or ICU admission, is essential for timely intervention and optimal resource allocation. However, predicting outcomes in sepsis is complicated by the absence of a single, reliable prognostic marker. This underscores the need for clinically applicable tools that can swiftly assess the severity of sepsis and guide critical care decisions, especially in emergency settings where time-sensitive management is vital [1, 2].

One of the first attempts to estimate the risk of adverse outcomes in sepsis patients was the systemic inflammatory response syndrome (SIRS) criteria, introduced as part of the Sepsis-1 definition. Using this definition, patients with a suspected or confirmed infection who scored positive on two out of the four criteria were diagnosed with sepsis [3]. Although the SIRS definition is straightforward to implement due to its simplicity, it has been criticized for its lack of specificity and sensitivity [4, 5]. Subsequently, in an attempt to improve diagnostic accuracy, the Sepsis-2 definition proposed to define sepsis in the presence of a suspected infection and any combination of one or multiple general, inflammatory, hemodynamic, organ dysfunction and of tissue hypoperfusion parameters, which would also allow to classify the severity of the septic patients [6]. However, the increasing number of criteria in the definitions made it more difficult to identify critically ill patients [7, 8].

To address these limitations, simpler scoring systems, like the Modified Early Warning Score (MEWS) [9] and National Early Warning Score system (NEWS and NEWS-2) [10, 11], have been proposed to identify patients with a worse prognosis, including but not limited to sepsis, at an early stage. The latest score, proposed by the Third International Consensus Definitions for Sepsis and Septic Shock (Sepsis-3), is the quick Sequential Organ Failure Assessment (qSOFA) [1, 12]. These alternative scores consider a wider range of clinical parameters compared to the traditional SIRS criteria. The MEWS and NEWS systems incorporate many variables for a more comprehensive assessment of patient’s risk. In contrast, qSOFA focuses specifically on three critical indicators of organ dysfunction, each of which is also part of the MEWS and NEWS systems.

Although these scoring systems enhance sensitivity and specificity in predicting adverse outcomes, their performance may vary across patient populations and clinical settings. One important factor that these scoring systems do not take into account is the site of infection, despite evidence suggesting that the location of infection is an independent predictor of patient outcomes. Sepsis is a highly heterogeneous condition [13, 14], and it is likely that a single scoring system may not adequately capture the full spectrum of its severity across different types of infections, that this component had been shown as an important, independent predictor of patient severity [15, 16]. However, this none of the scores mentioned above differentiate based on the site of infection. Accordingly, this study aims to compare the prognostic value of, MEWS, NEWS, NEWS-2, and qSOFA for in-hospital mortality, 30-day mortality and ICU admission, considering the site of infection, in patients presenting to the Emergency Department (ED) with a confirmed or suspected infection.

## Methods

### Participants and source of data

We performed a retrospective analysis using data obtained from the Acutelines data-biobank. Acutelines is a multi-disciplinary prospective hospital-based cohort study examining 24/7 the complete acute patient journey admitted to the ED of the University Medical Centre Groningen (UMCG), a tertiary care teaching hospital in the Netherlands. It employs a broad range of investigative procedures in assessing the pre-hospital, in-hospital, and long-term health factors that affect outcome in patients with acute conditions. The cohort population is broadly representative of the people living in the Northern Netherlands with acute medical conditions. Primary screening of patients for eligibility on arrival at the ED is performed 24 h a day by the ED nurse together with a trained research team. Bedside monitoring data were automatically captured and stored, and information from other data sources including the electronic health records of the hospital was securely imported via the electronic patient file (EPIC systems, Boston, MA, USA). More detailed information about the Acutelines cohort and participant selection can be found elsewhere [17, 18]. Participants were asked for written informed consent, when applicable by proxy or implied consent. The Acutelines cohort study is approved by the Institutional Review Board (IRB) of the UMCG, the Netherlands and registered under trial registration number NCT04615065 at ClinicalTrials.gov [19]. The current study was approved by the ethics committee of the UMCG (METC ID: 11236). The Strengthening the Reporting of Observational Studies in Epidemiology (STROBE) guidelines were followed.

For the current study, adult patients (≥ 18 years) who visited the ED for internal medicine, nephrology, geriatric medicine, pulmonology, rheumatology, gastro-enterology, medical oncology, urology or emergency medicine (non-trauma) [17] between September 2020 to July 2023 were screened. They were included in the study if at least one International Classification of Diseases 10 (ICD-10) codes for infection was registered at the ED working diagnoses, with either suspicion or diagnosis of infection (supplemental Table S1), no additional exclusion criteria were applied. Subsequently, patients were categorized based on the most prevalent infections. Undefined unique infections were assigned when the source was ambiguous or not well-defined, and “other” infections were designated when the frequency was less than 100; these patients were then aggregated.

### Outcome

The primary outcome was in-hospital mortality. Secondary outcomes included any ICU admission, and 30-day mortality.

### Sample size

For this study, we used a convenience sample, including all available cases from the Acutelines cohort, which determined a fixed sample size. Given this, we calculated the statistical power to detect the expected difference in the area under the receiver operating characteristic curve (AUC-ROC), using the fixed number of patients in the cohort. The type I error rate was set at 0.05 (Appendix 1).

### Predictors

Early Warning Scores Calculation: EWS (MEWS, NEWS, NEWS-2 and qSOFA) were computed by utilizing the first available clinical data from each patient within the initial 8 h post ED-admission, using the original variables and cutoffs (Table 1).

**Table 1:** Early warning scores comparison

Variable	MEWS		NEWS		NEWS-2		qSOFA	
	Cutoff	Points	Cutoff	Points	Cutoff	Points	Cutoff	Points
**Respiratory Rate (rpm)**	9–14	0	12–20	0	12–20	0	< 22	0
	15–20	1	9–11	1	9–11	1	≥ 22	1
	< 9 or 21–29	2	21–24	2	21–24	2	-	-
	≥ 30	3	≤ 8 or ≥ 25	3	≤ 8 or ≥ 25	3	-	-
**Oxygen Saturation** **(%)**	-	-	≥ 96	0	≥ 96	0	-	-
	-	-	94–95	1	94–95	1	-	-
	-	-	92–93	2	92–93	2	-	-
	-	-	≤ 91	3	≤ 91	3	-	-
Oxygen Saturation 2* **(%)**	-	-	-	-	88–92 or ≥ 93 on air	0	-	-
	-	-	-	-	86–87 or 93–94 on oxygen	1	-	-
	-	-	-	-	84–85 or 95–96 on oxygen	2	-	-
	-	-	-	-	≤ 83 or 95–96 on oxygen	3	-	-
**Supplemental Oxygen**	-	-	No	0	No	0	-	-
	-	-	Yes	2	Yes	2	-	-
**Systolic Blood Pressure (mmHg)**	101–199	0	111–219	0	111–219	0	> 100	0
	81–100	1	101–110	1	101–110	1	≤ 100	1
	71–80 or ≥200	2	91–100	2	91–100	2	-	-
	≤ 70	3	≤ 90 or ≥ 220	3	≤ 90 or ≥ 220	3	-	-
**Heart Rate (bpm)**	51–100	0	51–90	0	51–90	0	-	-
	41–50 101–110	1	41–50 or 91–110	1	41–50 or 91–110	1	-	-
	≤ 40 or 111–129	2	111–130	2	111–130	2	-	-
	≥ 130	3	≤ 40 or ≥ 131	3	≤ 40 or ≥ 131	3	-	-
**Mental status**	A	0	A	0	A	0	GCS = 15	0
	V	1	-	-			GCS < 15	1
	P	2	-	-			-	-
	U	3	V, P or U	3	C, V, P or U	3	-	-
**Temperature** **(°C)**	35–38.4	0	36.1–38	0	36.1–38	0	-	-
	-	-	35.1 – 36 38.1–39	1	35.1 – 36 38.1–39	1	-	-
	< 35 or > 38.5	2	≥ 39.1	2	≥ 39.1	2	-	-
	-	-	≤ 35	3	≤ 35	3	-	-
**Total points**	0–14		0–20		0–20		0–3	
**Cut off**	≥ 5		≥ 7		≥ 7		≥ 2	

*Separate scoring system for oxygen saturation in patients with type 2 respiratory failure (scale 2). Abbreviations: RMP: breaths per minute, BPM: beats per minute, A: Alert, V: Voice, P: Pain, U: Unresponsive, C: Confusion

### Missing data

We observed the presence of missing values in variables encompassed by the predictive scores, especially in the respiratory rate, Glasgow Coma Scale (GCS), temperature, and peripheral capillary oxygen saturation (SpO2). These variables were frequently not recorded in patients with stable condition. Consequently, in the primary analysis, missing data were treated as normal values, constituting a best-case scenario. Furthermore, a sensitivity analysis was performed with two additional models: the worst-case scenario, considering the missing data as abnormal values, and with a multivariate normal regression (MVN), multiple imputation technique, taking the vital signs, age, gender, Charlson Comorbidity Index and SOFA as independent values.

### Statistical analysis

The characteristics of the study population are presented as counts and percentages for the categorical variables, while continuous data are presented using medians and Interquartile Ranges (IQRs). To assess the predictive performance of each risk score for the outcomes given the source of infection, in terms of discrimination, we performed area under the receiver operating characteristics curve (AUC-ROC) based on the models defined as the sum of the corresponding predictors. The differences between the highest and the lowest AUC-ROC of each group were tested using the DeLong statistic [20]. The calibration was determined by the degree of correspondence given by the Hosmer-Lemeshow goodness-of-fit test (*p* > 0.05). Additionally, calibration curves were performed based on the results of the models in each of the groups. Predictive performance was assessed through the comparison of sensitivity, specificity, and positive and negative predictive values, applying Bayes’ theorem, while maintaining the original scores cutoffs: qSOFA ≥ 2, MEWS ≥ 5, NEWS and NEWS-2 ≥ 7. The threshold for statistical significance was < 0.05. Analyses were performed with Stata version 18^®^ (StataCorp).

## Results

**Fig. 1:**
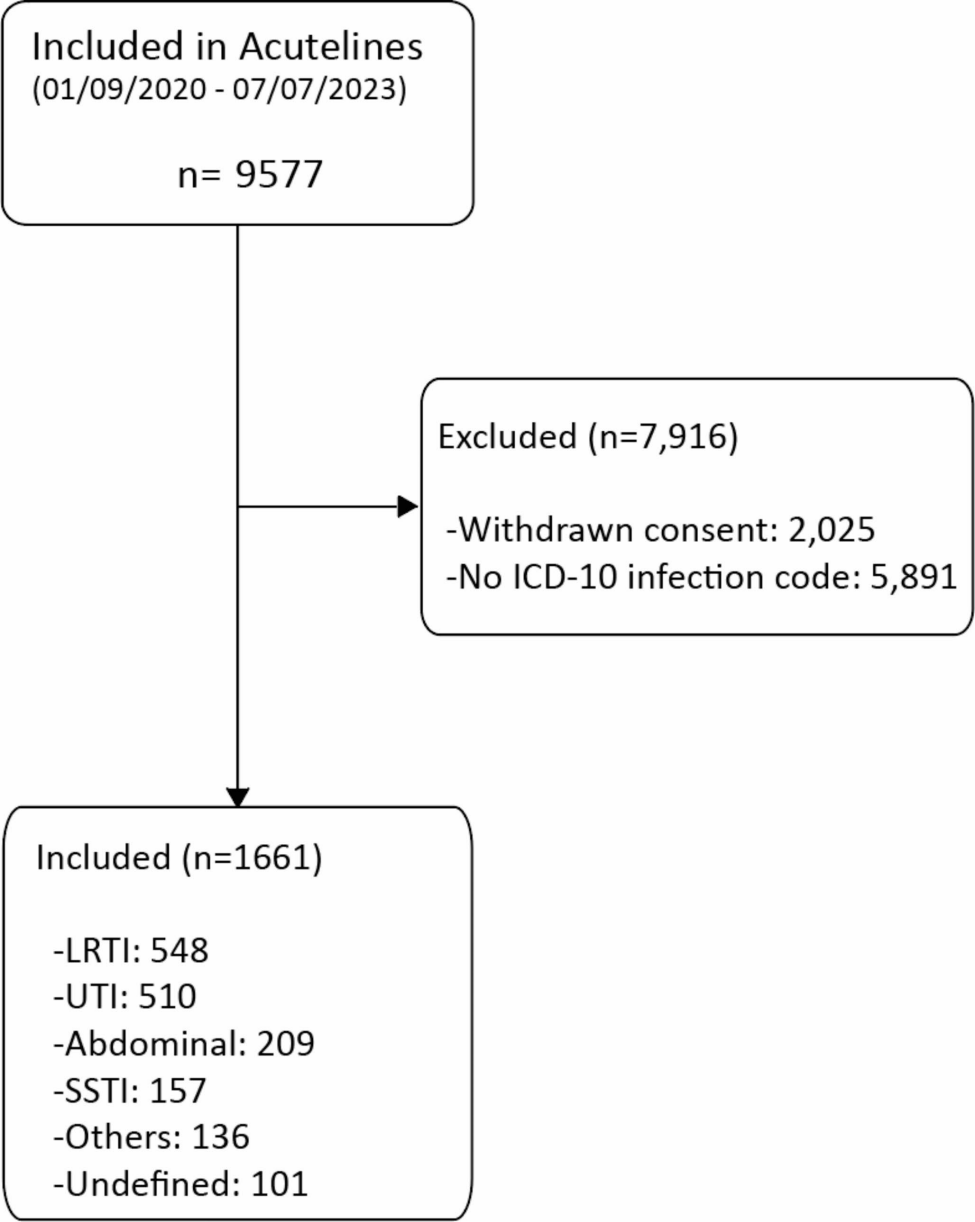
Flow diagram for study patients. Abbreviations: LRTI: Lower Respiratory Tract Infection, UTI: Urinary Tract Infection, SSTI: Skin and Soft Tissue Infection

A total of 1661 patients, who presented at the emergency department with a working diagnosis of infection, were enrolled in the study (Fig. 1). Among these patients, the distribution of infections was as follows: lower respiratory tract infections (LRTI) accounted for 548 (32.9%), urinary tract infections (UTI) for 510 (30.7%), abdominal infections for 209 (12.5%), skin and soft tissue infections (SSTI) for 157 (9.5%), and other types of infections for 136 (8.2%). Additionally, 101 (6.1%) of cases had suspected infection but an undefined primary source of infection. The category labeled “others” predominantly involved gastrointestinal infections (*n* = 63, 46.3%), but also included other types of infection such as otorhinolaryngological, central nervous system (CNS), bone and joint infections, endocarditis, and malaria. The median age of patients in the cohort was 67 years (IQR: 55–75), with 902 male visits (54.3%). The overall in-hospital mortality rate was 6.7% (*N* = 112). The subgroup analysis based on the source of infection showed, the highest in-hospital mortality rate in the category of undefined primary source infection at 14.9% (*n* = 15), followed by LRTI at 9.7% (*n* = 53). The lowest in-hospital mortality rate was identified in the SSTI group at 1.9% (*n* = 3). The overall Intensive Care Unit (ICU) admission rate was 7.1% (*n* = 118), with the highest ICU admission rate was documented in the undefined primary infection group at 11.9% (*n* = 12), followed by LRTI at 11.1% (*n* = 61), with the lowest observed in UTI at 2.8% (*n* = 14) (Table 2). In our cohort, 111 (6.7%) patients had neutropenia (i.e., neutrophil count < 0.5 × 106/mL). Notably, 14 of these cases fell into the undefined source group. Among the neutropenic patients, most cases (*n* = 60) had a lower respiratory tract infection (LRTI).

**Table 2:** Baseline characteristics of the study population, according to the source of infection

	LRTI *n* = 548	UTI *n* = 510	Abdominal *n* = 209	SSTI *n* = 157	Others *n* = 136	Undefined *n* = 101	Total *N* = 1661
**Characteristics**							
Male	326 (59.5%)	244 (47.8%)	103 (49.3%)	86 (54.8%)	82 (60.3%)	61 (60.4%)	902 (54.3%)
Age (median (IQR))	68 (58–74)	69 (57–76)	64 (49–73)	66 (54–76)	62 (45-73.5)	67 (56–75)	67 (55–75)
Patients with blood cultures (taken in the first 48 H)	418 (76.3%)	433 (84.4%)	176 (84.2%)	131 (83.4%)	101 (74.3%))	85 (84.2%)	1344 (80.9%)
Patient with positive blood cultures (taken in the first 48 H)	62 (14.83%) n: 418	136 (31.4%) n: 433	74 (42.1%) n: 176	40 (30.5%) n: 131	31 (30.7%) n: 101	22 (25.9%) n: 85	365 (27.2%) n: 1344
**Severity**							
Charlson median (IQR)	4 (2–6)	3 (2–6)	3 (2–6)	4 (2–6)	4 (2–6)	5 (2–7)	4 (2–6)
SOFA 24 H median (IQR)	3 (1–4)	2 (1–4)	3 (2–4)	2 (0–4)	2 (1–4)	4 (2–6)	2 (1–3)
Triage color							
Green	0	1 (0.2%)	0	2 (1.3%)	0	0	3 (0.2%)
Yellow	329 (60%)	365 (71.6%)	159 (76.1%)	113 (72%)	101 (74.3%)	62 (61.4%)	1129 (68%)
Orange	200 (36.5%)	134 (26.3%)	43 (20.6%)	37 (23.6%)	30 (22.1%)	33 (32.7%)	477 (28.7%)
Red	7 (1.3%)	1 (0.2%)	1 (0.5%)	1 (0.6%)	2 (1.5%)	2 (1.3%)	14 (0.8%)
**Variables**							
Respiratory Rate (rpm) (median (IQR))	22 (18–26) n: 473	19 (16–24) n: 399	18 (16–22) n: 171	20 (16–23) n: 120	18 (15–22) n: 101	21 (18–24) n: 81	20 (16–24) n: 1345
Heart Rate (bpm) (median (IQR))	100 (87–114)	95 (83–109)	99 (84–111)	95.5 (84–111) n: 154	96.5 (82-101.5)	105 (88–116)	99 (85–112) n: 1658
Systolic blood pressure (mmHg) (median (IQR))	129 (112-145.5)	125 (110–144) n: 507	127 (109–141)	124 (110–138) n: 154	127 (110–145)	121 (105–138)	126 (110–143) n: 1655
Diastolic blood pressure (mmHg) (median (IQR))	76 (65–87)	73 (63–84) n: 507	73 (63–83)	72 (62–82) n: 154	77 (67.5–88.5)	73 (64–81)	64 (74–85) n: 1655
Glasgow coma score (median (IQR))	15 (15–15) n: 515	15 (15–15) n: 499	15 (15–15) n: 204	15 (15–15) n: 153	15 (15–15) n: 134	15 (14–15) n: 94	15 (15–15) n: 1599
Temperature (°C) (median (IQR))	37.3 (36.8–38.1) n: 546	37.2 (36.7–38.1) n: 506	37.3 (36.7–38.1) n: 206	37.4 (36.8–38.2) n: 153	37.3 (36.6–38.0) n: 135	37.7 (36.8–38.3) n: 100	37.3 (36.7–38.1) n: 1646
SpO2 (%) (median (IQR))	97 (93–97) n: 546	97 (95–98) n: 504	97 (96–99) n: 207	97 (95–99) n: 153	98 (96–99) n: 135	96 (95–98) n: 99	97 (94–98) n: 1644
**Scores**							
MEWS median (IQR)	3 (2–4)	2 (1–3)	2 (1–4)	2 (1–4)	2 (1–4)	3 (1–4)	2 (1–4)
NEWS median (IQR)	5 (3–7)	3 (1–5)	2 (1–5)	3 (1–5)	2 (1–4)	5 (3–6)	4 (2–6)
NEWS- 2 median (IQR)	5 (3–7)	3 (1–5)	2 (1–5)	3 (1–6)	2.5 (1–4)	5 (3–7)	4 (2–6)
qSOFA median (IQR)	1 (1–2)	1 (0–1)	0 (0–1)	0 (0–1)	0 (0–1)	1 (0–1)	0 (0–1)
**Outcomes**							
ICU admission	61 (11.1%)	14 (2.8%)	12 (5.7%)	11 (7.0%)	8 (5.9%)	12 (11.9%)	118 (7.1%)
In-hospital mortality	53 (9.7%)	24 (4.7%)	10 (4.8%)	3 (1.9%)	7 (5.2%)	15 (14.9%)	112 (6.7%)
30-day mortality	77 (14.1%)	42 (8.2%)	17 (8.1%)	5 (3.2%)	11 (8.1%)	16 (15.8%)	168 (10.11%)

Abbreviations: LRTI: Lower Respiratory Tract Infection, UTI: Urinary Tract Infection, SSTI: Skin and Soft Tissue Infection, IQR: Interquartile Range, RPM: breaths per minute, BPM: beats per minute, ICU: Intensive Care Unit

**Fig. 2:**
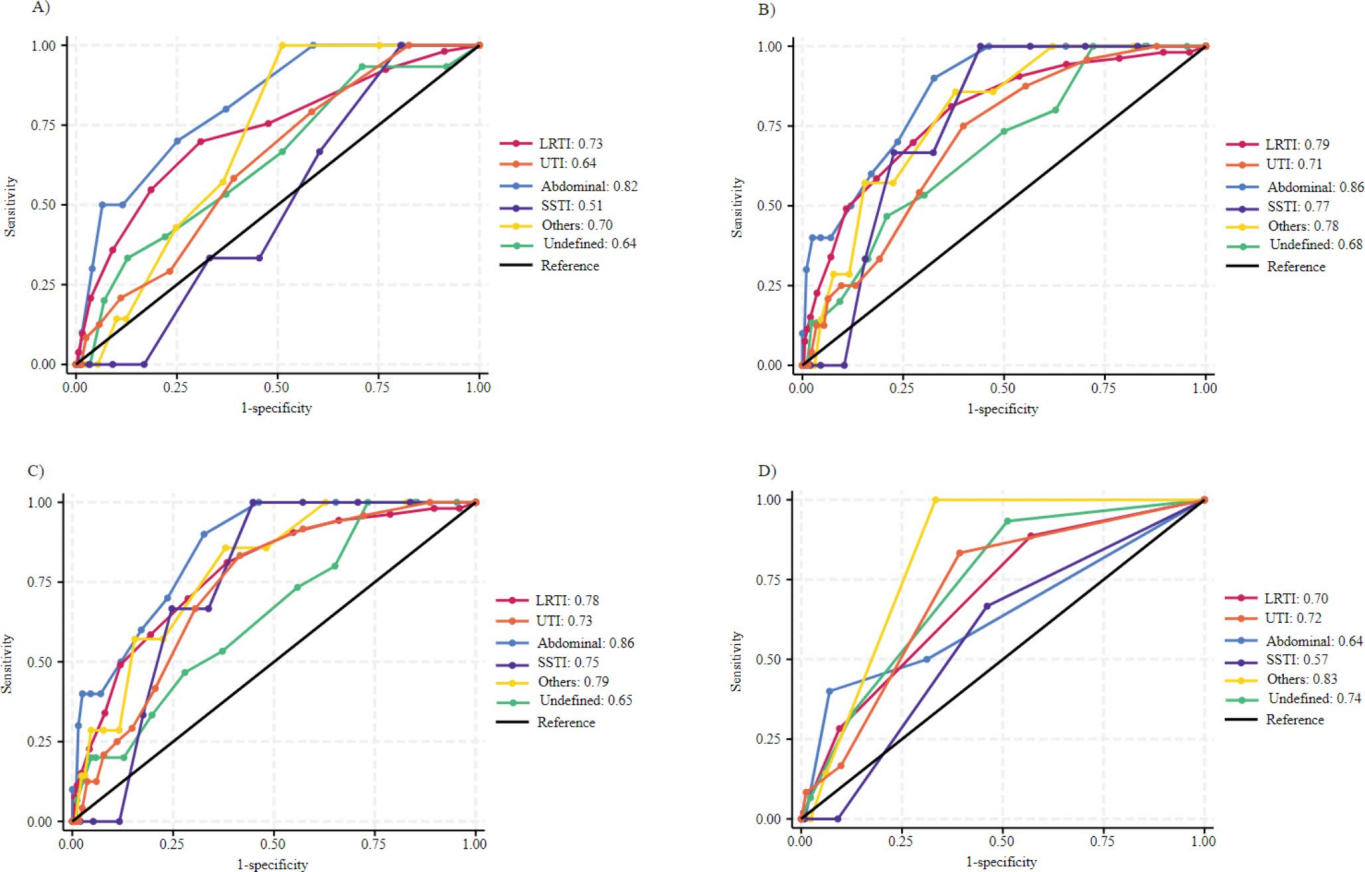
Receiver operating characteristic curve in the different subcohorts for EWS in the prediction of in-hospital mortality. **A**) MEWS **B**) NEWS **C**) NEWS-2 **D**) qSOFA

The AUC ROC for predicting in-hospital mortality based on the NEWS and NEWS-2 was highest (both scoring 0.86 (95% CI: 0.76–0.95)) in patients with abdominal infections and lowest in patients with an undefined primary source of infection (0.68 (95% CI: 0.55–0.82, *p* = 0.04) and 0.65 (95% CI: 0.51–0.79, *p* = 0.02, respectively). Similarly, MEWS demonstrated a higher AUC-ROC for predicting mortality in patients with abdominal infections (0.82, 95% CI: 0.70–0.94) compared to those with urinary tract infections, which had a lower AUC-ROC of 0.64 (95% CI: 0.54–0.73, *p* = 0.02). The qSOFA showed the highest AUC-ROC for mortality in patients with other infections group (0.83, 95% CI: 0.76–0.89) and the lowest for those with skin and soft tissue infections (0.57, 95% CI: 0.27–0.87), with a non-significant difference (*p* = 0.11) (Fig. 2 and Table S2).

**Fig. 3:**
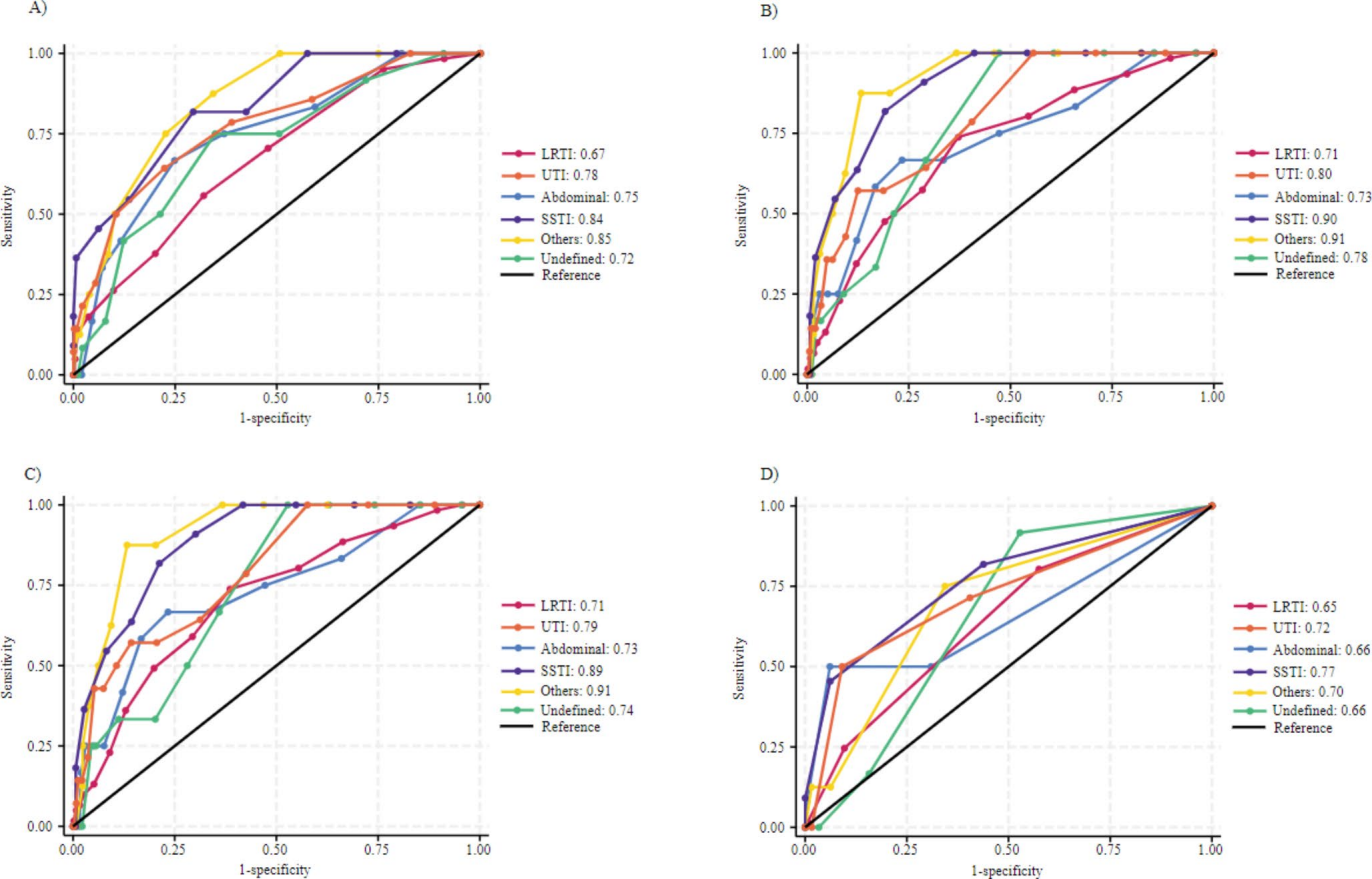
Receiver operating characteristic curve in the different subcohorts for EWS in the prediction of ICU admission. **A**) MEWS **B**) NEWS **C**) NEWS-2 **D**) qSOFA

**Fig. 4:**
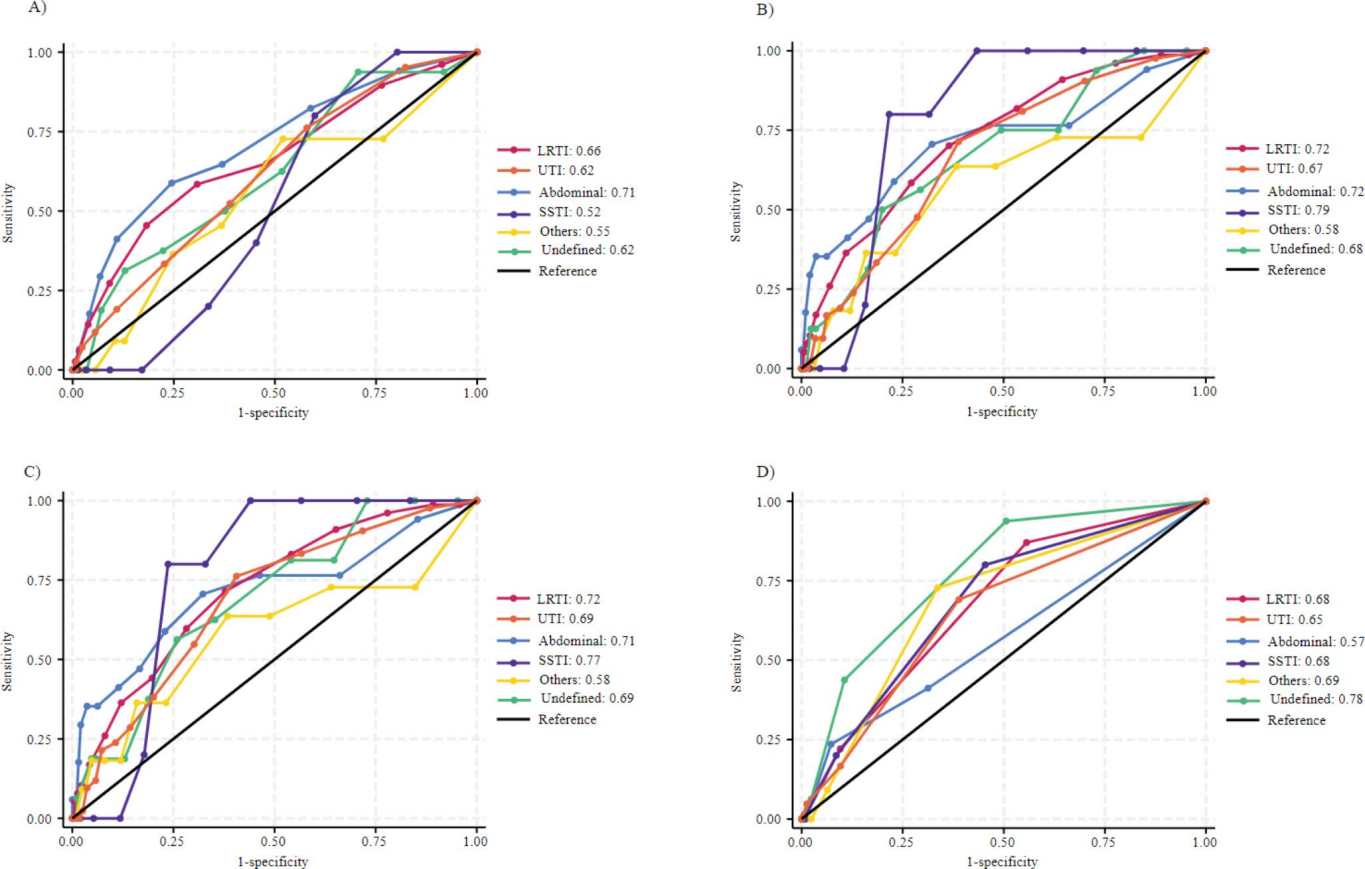
Receiver operating characteristic curve in the different subcohorts for EWS in the prediction of 30-day mortality. **A**) MEWS **B**) NEWS **C**) NEWS-2 **D**) qSOFA

In predicting ICU admission, the group of patients with other infections reached the highest AUC-ROC in NEWS and NEWS-2, both scoring 0.91 (95% CI: 0.84–0.98), and MEWS with an AUC-ROC of 0.85 (95% CI: 0.74–0.95). On the contrary, qSOFA reached its highest AUC-ROC in patients with SSTI at 0.77 (95% CI: 0.61–0.93). The lowest AUC-ROC values for ICU admission prediction were observed for LRTI across all four scores: MEWS at 0.67 (95% CI: 0.61–0.74), NEWS at 0.71 (95% CI: 0.64–0.78), NEWS-2 at 0.71 (95% CI: 0.64–0.77), and qSOFA at 0.65 (95% CI: 0.58–0.71) (Fig. 3 and Table S3). The results for predicting 30-day mortality are shown in Fig. 4 and Table S4.

The calibration of the models was adequate in the most of the subcohorts for predicting admission to the ICU, as well as in-hospital and 30-day mortality outcome, according to the Hosmer-Lemeshow statistic (*p* > 0.05) (Table S5, S6, S7). Additionally, calibration curves were performed for all the outcomes in the different models in each of the subcohorts, and a high degree of correspondence of the scores was shown in most of the subcohorts, in some cases calibration graph could not be obtained by insufficient quantiles (Supplementary Figure S1-S12).

Regarding the performance of the models in their operative characteristics, the greatest sensitivity for in-hospital mortality was for NEWS and NEWS-2 for LRTI (69.8%, 95% confidence interval (CI): 65.97 -73.65%) while the best specificity was for qSOFA for other sources at 93.8% (95% CI: 89.74 -97.85%). The greatest specificity for ICU admission was for NEWS and NEWS-2 for SSTI (63.64% 95% CI: 56.11 -71.16%) while the best specificity was for qSOFA for other sources at 93.75% (95% CI: 89.68 -97.82%) (Tables S2 -S4).

## Discussion

In this study, we evaluated the prognostic accuracy of EWS to predict in-hospital mortality, 30-day mortality, and ICU admission, considering the source of infection. To our knowledge, this is the first study to stratify the performance of EWS by infection type. Our results reveal that the predictive accuracy of EWS varies according to the infection source, highlighting the importance of considering infection type in clinical risk assessment, a feature that is rarely explored. This unique aspect of our analysis provides valuable insights into how EWS might be optimized for different clinical contexts, emphasizing the need for more personalized approaches in risk stratification.

In the case of LRTI, the NEWS was best in predicting in-hospital mortality (AUC = 0.79) and the qSOFA (AUC = 0.70) had the lowest performance. The performance was relatively consistent across the different EWS, almost always among the best performances, and never the worst in any of the EWS. For the UTI the best predicting in-hospital mortality was the NEWS-2 (AUC = 0.73), and the lowest MEWS (AUC = 0.64). Abdominal infection has the best performance in predicting in-hospital mortality for the NEWS, NEWS-2 and MEWS (AUC = 0.86, 0.86 and 0.82 respectively), however, it was the second lowest for the qSOFA (AUC = 0.64). Overall, calibration was good across all scores, except for qSOFA in different subgroups across the different outcomes (Tables S5-S7, Figures S1-S12). This is likely due to the limited number of variables in qSOFA, reducing its ability to accurately capture varying risk levels. These findings are particularly notable because none of these EWS were developed with a specific focus on the infection source, and some were not even exclusively designed for patients with infections. This highlights a potential limitation of using universal EWS across diverse infection types, as a “one-size-fits-all” approach may compromise accuracy for certain infections [9–11]. In addition, our cohort includes patients with COVID-19 infections, which was not considered when these EWS were developed. Despite this, the proportion of patients with LRTI were comparable to those reported globally in the pre-COVID era [21–23], from the 548 patients with LRTI, only 43 (7.85%) were positive with COVID-19. This reinforces the robustness of our results and underscores the need for future EWS development to consider the infection source, as it significantly influences the predictive accuracy of these scores.

Another important consideration is the accuracy of diagnoses at the ED and the timing of calculating EWS considering the dynamic nature of variables included in these scores. These are critical factors, particularly in elderly patients with multiple comorbidities, where initial diagnoses may be unclear, or competing diagnoses may exist. Relying solely on ICD-10 data introduces some uncertainty, as diagnoses often evolve during a patient’s hospital stay. To minimize the risk of missing relevant cases due to miscoding, we included not only the ICD-10 codes for confirmed infections but also those used as working diagnoses or suspected infections. Additionally, we used the broadest possible level of ICD-10 classification to identify “infection” (Table S1). Early identification of the infection is crucial, as a consequence, the timing of EWS calculation may affect its performance in the risk stratification process, particularly in elderly, and highly comorbid patients. This underlines the relevance of more dynamic and flexible tools, particularly in patients with frailty and others at higher risk for deterioration.

Although there is no direct comparison of the performance of EWS based on the source of infection, previous studies have demonstrated a difference in prognosis depending on the site of infection and the type of microorganism [24, 25]. For instance, Oduncu et al. compared qSOFA, SIRS, and NEWS scores. Their cohort had a higher ICU admission rate (17.3% vs. 7% in our cohort) and higher 30-day mortality (18.1% vs. 10.1%). Although they did not directly compare the infection source with the performance of the scores, they did present outcomes based on the infection source. For example, in their cohort, respiratory infections accounted for 39% of cases, with a mortality rate of 23.2% and an ICU admission rate of 30.9%. UTI comprised 23.9%, with a mortality rate of 10.8% and ICU admission rate of 9%. Abdominal infections made up 17.3% of cases, with 12.5% mortality and 5% ICU admissions. Soft tissue infections accounted for 6.9%, with 6.3% mortality and 3% ICU admissions. Other infections made up 6.6%, with 29% mortality and 12.9% ICU admissions, while undetermined infections comprised 6%, with 32% mortality and 17% ICU admissions [26]. Notably, our cohort had a higher representation of urinary tract infections (30.7%). Despite this, their results, like ours, show that mortality varies depending on the infection source, further suggesting that sepsis prediction scores should consider the origin of infection for more accurate assessments.

This discrepancy raises a critical question: should we strive to develop a universal EWS that performs equally well across all infection foci, or should we tailor EWS to specific types of infections? A universal score would simplify clinical protocols but may compromise accuracy for certain infection types. On the other hand, specialized scores could provide more precise predictions but add complexity to clinical decision-making. For instance, our data show that the performance of EWS for predicting in-hospital mortality for LRTI falls between those for abdominal infections and UTIs. Given that abdominal and UTI impacts on respiratory variables are more comparable, the differences in EWS performance underscore the need for tailored approaches. Consequently, our results could contribute to the development of a new sepsis scoring system where the type of infection will be considered alongside other prognostic factors. One potential solution to this issue could be the implementation of a hybrid model, where a general EWS is used initially, followed by infection-specific adjustments once the infection source is identified. This approach could balance the need for both simplicity and accuracy. Additionally, machine learning algorithms could be employed to dynamically adjust the weight of different variables in the EWS based on real-time data, potentially providing a more nuanced and accurate risk assessment for diverse patient populations. Further research into the development and validation of such adaptive scoring systems is needed.

One of the limitations of our study was the sample size. We based the difference of 0.6–0.7 in the discrimination (AUC-ROC) of qSOFA in a cohort of UTI versus LRTI on information from Madrazo et al. and Kolditz et al. [27, 28]. However, this difference between AUC may not hold clinical significance. Furthermore, the small sample size in our study, particularly in some subgroups, resulted in limited statistical power, increasing the likelihood of a type II error. However, despite these limitations, we were still able to demonstrate notable differences within these smaller subgroups, such as in patients with abdominal infections. Thus, although the relatively small size may have precluded us from identifying more differences between groups, it does not affect the conclusion that the performance of the EWS depends on the type of infection. Importantly, these results highlight the need for further research and should encourage other research groups to investigate this area more comprehensively with larger cohorts to validate and expand upon our findings. Another potential limitation is that we only included patients with suspected infections at the ED, but did not include patients diagnosed with nosocomial infections. The study was conducted at a single, tertiary university hospital, which serves as a reference for the northern region. However, the large catchment region of our hospital leads to a diverse case-mix of both academic and non-academic care, which increases the generalizability of our findings to other, non-academic centers. Finally, concerning our outcomes, the use of all-cause in-hospital mortality as the primary outcome, instead of infection related mortality, might have led to an overestimation of the actual mortality attributable to infections, as patients could have died to various other causes. Nevertheless, our mortality rate aligns with those reported in previous studies [12, 29]. It is also important to note that the median length of stay in our cohort was only 4 days, largely due to our healthcare system’s focus on resolving as much as possible outside the hospital, supported by the necessary infrastructure to do so safely. This minimizes the potential for not infection-related factors to influence our results in-hospital mortality. Regarding one of our secondary outcome, the EWS used in any hospital may affect ICU admission based on local guidelines in place. However, at the ED in the UMCG we use the Emergency Severity Index (ESI) as triage tool and do not have an additional EWS. The decision to admit a patient to the ICU, is not based on triage urgency, but depends on the physician’s decision. The EWS in use at wards in the UMCG is a modified MEWS that includes the following additional criteria beyond the original model: (1) urine production < 75 mL in the last 4 h and (2) any concern raised by healthcare staff regarding the patient’s status. The UMCG-MEWS did not outperform other EWS systems in predicting ICU admissions in this cohort. This may reflect limitations in its design or compliance with its use. Although the UMCG-MEWS score informs the nurse when to call the physician, no protocol at UMCG ties triage urgency or MEWS directly to ICU admission. Hence, discrepancies between predicted and actual ICU admissions likely stem from variation in clinical decision-making and adherence to EWS. These observations highlight the complexity of relying on a single scoring system and the need for ongoing evaluation of performance in real-world settings.

One of the strengths of our study was the collection of prospective data, resulting in fewer missing values and higher quality data. Additionally, we included calibration in our analysis, a crucial component, often overlooked in similar studies. Calibration assesses the agreement between predicted and observed outcomes, which is essential since poor calibration can lead to risk misclassification. Even a model with good discrimination (e.g., high AUC) can be poorly calibrated if predicted probabilities do not align with observed event rates across risk levels. In this study, we aimed to assess both discrimination and calibration to provide a comprehensive evaluation of model performance (Appendix 2, Tables S-S7, Figures S1-S12).

## Conclusion

Our results suggest variations in the prognostic performance of EWSs for in-hospital mortality based on the site of infection. These findings underscore the importance of considering infection source when developing EWSs for risk prediction and patient management in clinical practice.

## Electronic supplementary material

Below is the link to the electronic supplementary material.

Supplementary Material 1

## Data Availability

Data and script are available upon reasonable request.
