# Supplementary material for: Validation of MEWS, NEWS, NEWS-2 and qSOFA for different infection foci at the emergency department, the acutelines cohort
Source: Eur J Clin Microbiol Infect Dis. 2024 Oct 16;43(12):2441–52. doi: 10.1007/s10096-024-04961-1 (PMC11608163; doi:10.1007/s10096-024-04961-1)
Supplement: Supplementary file 1 — Supplementary Material 1 [file 10096_2024_4961_MOESM1_ESM.docx]

**Supplemental material**

**Table S1.** International Classification of Diseases (ICD) 10th Revision codes for the identification of infection

**Table S2.** Operative characteristics for in-hospital mortality prediction.

**Table S3.** Operative characteristics for ICU admission prediction.

**Table S4.** Operative characteristics for in 30 day mortality prediction.

**Table S5.** Calibration of the models for in-hospital mortality.

**Table S6.** Calibration of the models for ICU admission.

**Table S7.** Calibration of the models for 30-day mortality.

**Figure S1.** Calibration curves for ICU admission-MEWS A) LRTI B) UTI C) Abdominal D) SSTI E) Others F) Undefined.

**Figure S2.** Calibration for ICU admission-NEWS A) LRTI B) SSTI C) Others D) Undefined.

**Figure S3.** Calibration curves for ICU admission-NEWS2 A) LRTI B) UTI C) Abdominal D) SSTI E) Others F) Undefined.

**Figure S4.** Calibration curves for ICU admission-qSOFA A) LRTI B) SSTI.

**Figure S5.** Calibration curves for In-hospital mortality-MEWS A) LRTI B) Abdominal

**Figure S6.** Calibration curves for In-hospital mortality-NEWS A) LRTI B) Abdominal C) Undefined

**Figure S7.** Calibration curves for In-hospital mortality-NEWS2 A) LRTI B) Abdominal C) Others D) Undefined

**Figure S8.** Calibration curves for In-hospital mortality-qSOFA A) LRTI B) Undefined

**Figure S9.** Calibration curves for 30-day mortality-MEWS A) LRTI B) Abdominal

**Figure S10.** Calibration curves for 30-day mortality-NEWS A) LRTI B) Abdominal C) Undefined

**Figure S11.** Calibration curves for 30-day mortality-NEWS2 A) LRTI B) UTI C) Abdominal D) Undefined

**Figure S12.** Calibration curves for 30-day mortality-qSOFA A) LRTI B) Undefined

**Figure S13.** Worst case scenario. Receiver operating characteristic curve in the different subcohorts for EWS in the discrimination of inhospital mortality. A) MEWS B) NEWS C) NEWS-2 D) qSOFA.

**Figure S14.** Worst case scenario. Receiver operating characteristic curve in the different subcohorts for EWS in the discrimination of ICU admission. A) MEWS B) NEWS C) NEWS-2 D) qSOFA.

**Figure S15.** Worst case scenario. Receiver operating characteristic curve in the different subcohorts for EWS in the discrimination of 30-day mortality. A) MEWS B) NEWS C) NEWS-2 D) qSOFA.

**Figure S16.** Imputed data. Receiver operating characteristic curve in the different subcohorts for EWS in the discrimination of inhospital mortality. A) MEWS B) NEWS C) NEWS-2 D) qSOFA.

**Figure S17.** Imputed data. Receiver operating characteristic curve in the different subcohorts for EWS in the discrimination of ICU admission. A) MEWS B) NEWS C) NEWS-2 D) qSOFA.

**Figure S18.** Imputed data. Receiver operating characteristic curve in the different subcohorts for EWS in the discrimination of 30-day mortality. A) MEWS B) NEWS C) NEWS-2 D) qSOFA.

**Appendix 1.** Power calculation.

**Appendix 2.** Pseudo-calibration analysis.

**Table S1.** International Classification of Diseases (ICD) 10th Revision codes for the identification of infection

| **ICD-10** |
| --- |
| A01-A02.0, A02.1-A02.9, A03-A09.9, A19-A20.3, A20.7-A20.9, A21-A21.3, A21.7-A21.9, A22-A22.2, A22.7-A22.9, A23-A24.0, A24.1-A24.9, A25-A26.0, A26.7-A26.9, A27-A28.1, A28.2-A28.9, A31-A32.12, A32.7-A32.9, A36-A39, A39.0, A39.1-A39.3, A39.4-A41.9, A42-A42.2, A42.7- A42.9, A43-A46.0, A48-A49.9, A50-A50.9, A54.86, A59-A59.9, A65-A65.0, A69-A69.1, A74, A74.8- A75.9, A77-A81.9, A83-A96.9, A98-B00.59, B00.7-B00.9, B01-B10.89, B25-B27.99, B29.4, B33-B34.9, B37-B37.6, B37.7-B37.9, B38-B50.9, B54-B55, B55.1-B55.9, B58- B60.8, B64, B67-B67.99, B91, B95-B99.9, G00-G08.0, G14-G14.6, H05.01-H05.039, H60.2-H60.23, H70.0-H70.009, I00, I02, I02.9, I26.01- I26.09, I26.90-I26.99, I33-I33.9, I38-I39.9, I40.0-I40.9, I76, I96-I96.9, I98.1, J01-J06.9, J09-J22.9, J36-J36.0, J39.0-J39.1, J85-J86.9, K35- K37.9, K57-K57.93, K61-K61.4, K63.0-K63.1, K65-K65.9, K67.8, K75.0- K75.1, K75.3, K76.3, K77.0, K81.0, K81.2, K83.0, K95.01, K95.81, L02- L08.9, M00-M02.9, M86-M86.9, M89.6-M89.69, N10-N10.9, N15.1-N15.9, N30-N30.91, N39.0, N41.0, N41.2-N41.3, N45-N45.9, N70-N77.8, N98.0, O03.0, O03.38, O03.5, O03.88, O04.5, O04.88, O07.38, O08.0, O08.83, O23-O23.93, O41.1-O41.93, O75.3, O85- O86.89, O88.3-O88.32, O91- O91.23, O98, O98.2-O98.93, P00.2, P22-P23.9, P29.12, P29.81, P35-P37, P37.1-P39.9, R65.2-R65.21, R68.13, R78.81, T80.2-T80.29, T81.4, T82.6-T82.7, T83.5, T83.6, T84.5-T84.7, T85.7, T88.0, U04. |

**Table S2.** Operative characteristics for in-hospital mortality prediction.

|  | **Sensitivity**  **(CI 95%)** | **Specificity**  **(CI 95%)** | **LR +**  **(CI 95%)** | **LR-**  **(CI 95%)** | **PPV**  **(CI 95%)** | **NPV**  **(CI 95%)** | **AUC-ROC**  **(CI 95%)** |
| --- | --- | --- | --- | --- | --- | --- | --- |
| **MEWS** | | | | | | | |
| **LRTI** | 54.72% (50.55% - 58.88%) | 81.41% (78.16% - 84.67%) | 2.94  (2.17- 4.00) | 0.56  (0.41 - 0.75) | 23.97%  (20.39% - 27.54%) | 94.38%  (92.45% - 96.31%) | 0.73  (0.65- 0.81) |
| **UTI** | 20.83% (17.31% - 24.36%) | 88.89% (86.16% 91.62%) | 1.87  (0.83 - 4.25) | 0.89  (0.72 - 1.10) | 8.47%  (6.06%- 10.89%) | 95.79% (94.04% - 97.53%) | 0.64 (0.54- 0.73) |
| **Abdominal** | 50.00% (43.22% 56.78%) | 88.44% (84.11% 92.78%) | 4.33  (2.09 - 8.97) | 0.57  (0.30 - 1.05) | 17.86% (12.66% 23.05%) | 97.24% (95.02% 99.46%) | 0.82 (0.70- 0.94) |
| **SSTI** | 0 | 83.12% (77.26% 88.98%) | 0 | 1.20  (1.12 - 1.29) | 0 | 97.71% (95.37% 100%) | 0.51 (0.24- 0.77) |
| **Others** | 14.29% (8.40%- 20.17%) | 87.60% (82.06%- 93.14%) | 1.15  (0.18- 7.49) | 0.98  (0.72- 1.33) | 5.88% (1.93%- 9.84%) | 94.96% (91.28%- 98.64%) | 0.70 (0.58- 0.83) |
| **Undefined** | 40.00% (30.45% - 49.55%) | 77.91% (69.82%- 86.00%) | 1.81  (0.87- 3.78) | 0.77  (0.50 - 1.18) | 24.00% (15.67%- 32.33%) | 88.16% (81.86%- 94.46%) | 0.64 (0.49 - 0.79) |
| **NEWS** | | | | | | | |
| **LRTI** | 69.81% (65.97% -73.65%) | 72.53% (68.79%-76.26%) | 2.54  (2.02- 3.19) | 0.42  (0.28 - 0.63) | 21.39% (17.95% -24.82%) | 95.73% (94.04% - 97.43%) | 0.79 (0.72 -0.85) |
| **UTI** | 25.00% (21.24% -28.76%) | 86.83% (83.90%-89.77%) | 1.90  (0.92- 3.94) | 0.86  (0.68 - 1.09) | 8.57% (6.14% -11.00%) | 95.91% (94.19%-97.63%) | 0.71  (0.62-0.79) |
| **Abdominal** | 50.00% (43.22% -56.78%) | 87.94% (83.52%-92.35%) | 4.15  (2.01- 8.56) | 0.57  (0.31- 1.06) | 17.24% (12.12% -22.36%) | 97.22% (94.99% -99.45%) | 0.86 (0.76-0.95) |
| **SSTI** | 33.33% (25.96% -40.71%) | 84.42% (78.74%-90.09%) | 2.14  (0.41 - 11.05) | 0.79  (0.35- 1.76) | 4.00% (0.93% -7.07%) | 98.48% (96.57% -100.40%) | 0.77 (0.61-0.92) |
| **Others** | 28.57% (20.98% -36.16%) | 88.37% (82.98%-93.76%) | 2.46  (0.69- 8.70) | 0.81  (0.50- 1.30) | 11.76% (6.35% -17.18%) | 95.80% (92.43%-99.17%) | 0.78 (0.64-0.92) |
| **Undefined** | 46.67% (36.94% -56.40%) | 79.07% (71.14%-87.00%) | 2.23  (1.13 - 4.40) | 0.68  (0.41 - 1.10) | 28.00% (19.24% -36.76%) | 89.47% (83.49% -95.46%) | 0.68 (0.55-0.82) |
| **NEWS-2** | | | | | | | |
| **LRTI** | 69.81% (65.97% -73.65%) | 71.31% (67.53%-75.10%) | 2.43  (1.94- 3.05) | 0.42  (0.28- 0.64) | 20.67% (17.28%-24.06%) | 95.66% (93.96% -97.37%) | 0.78 (0.72- 0.84) |
| **UTI** | 29.17% (25.22% -33.11%) | 85.19% (82.10% -88.27%) | 1.97  (1.02- 3.81) | 0.83  (1.02- 3.81) | 8.86% (6.39%-11.33%) | 96.06% (94.37%-97.74%) | 0.74 (0.65- 0.81) |
| **Abdominal** | 50.00% (43.22% -56.78%) | 87.94% (83.52%-92.35%) | 4.15  (2.01 - 8.56) | 0.57  (0.31 - 1.06) | 17.24% (12.12% -22.36%) | 97.22% (94.99% -99.45%) | 0.86 (0.76 - 0.95) |
| **SSTI** | 33.33% (25.96% -40.71%) | 82.47% (76.52% -88.42%) | 1.90  (0.37- 9.77) | 0.81  (0.36- 1.81) | 3.57% (0.67% - 6.47%) | 98.45% (96.52% 100.38%) | 0.75 (0.59 - 0.91) |
| **Others** | 28.57% (20.98% -36.16%) | 88.37% (82.98%-93.76%) | 2.46  (0.69 - 8.70) | 0.81  (0.50 - 1.30) | 11.76% (6.35% - 17.18%) | 95.80% (92.43% 99.17%) | 0.79  (0.64- 0.94) |
| **Undefined** | 46.67% (36.94% -56.40%) | 72.09% (63.35% -80.84%) | 1.67  (0.88 - 3.17) | 0.74  (0.45 - 1.21) | 22.58% (14.43%-30.73%) | 88.57% (82.37%-94.78%) | 0.65 (0.51 - 0.79) |
| **qSOFA** | | | | | | | |
| **LRTI** | 28.30% (24.53% -32.07%) | 90.51% (88.05%-92.96%) | 2.98  (1.79- 4.95) | 0.79  (0.67- 0.94) | 24.19% (20.61%-27.78%) | 92.18% (89.93% -94.43%) | 0.70 (0.64 -0.76) |
| **UTI** | 16.67% (13.43% -19.90%) | 90.12% (87.53% -92.71%) | 1.69  (0.66 - 4.29) | 0.93  (0.77- 1.11) | 7.69% (5.38% -10.00%) | 95.63% (93.86% -97.41% | 0.72 (0.63 - 0.80) |
| **Abdominal** | 40.00% (33.36% -46.64%) | 92.96% (89.50% -96.43%) | 5.69  (2.28 - 14.15) | 0.65  (0.39 - 1.07) | 22.22% (16.59%-27.86%) | 96.86% (94.49%-99.22%) | 0.64 (0.44 -0.83) |
| **SSTI** | 0 | 90.91% (86.41% -95.41%) | 0 | 1.10  (1.05- 1.16) | 0 | 97.90% (95.66%-100%) | 0.57 (0.27 - 0.87) |
| **Others** | 14.29% (8.40% -20.17%) | 93.80% (89.74% -97.85%) | 2.30  (0.33 - 15.95) | 0.91  (0.67 - 1.24) | 11.11% (5.83%-16.39%) | 95.28% (91.71% -98.84%) | 0.83 (0.76 - 0.89) |
| **Undefined** | 33.33% (24.14% -42.53%) | 87.21% (80.70% -93.72%) | 2.60  (1.06 - 6.43) | 0.76  (0.53 - 1.10) | 31.25% (22.21% - 40.29%) | 88.24% (81.95% -94.52%) | 0.74 (0.63 - 0.85) |

Abbreviations: LRTI: Lower Respiratory Tract Infection, UTI: Urinary Tract Infection, SSTI: Skin and Soft Tissue Infection.

Cutoffs by scores: qSOFA ≥2, MEWS ≥5, NEWS and NEWS-2 ≥7.

**Table S3.** Operative characteristics for ICU admission prediction.

|  | **Sensitivity**  **(CI 95%)** | **Specificity**  **(CI 95%)** | **LR +**  **(CI 95%)** | **LR-**  **(CI 95%)** | **PPV**  **(CI 95%)** | **NPV**  **(CI 95%)** | **AUC-ROC**  **(CI 95%)** |
| --- | --- | --- | --- | --- | --- | --- | --- |
| **MEWS** | | | | | | | |
| **LRTI** | 37.7% (33.7% - 41.8%) | 79.88% 76.52% -83.23%) | 1.87  (1.30 - 2.71) | 0.78  0.64 - 0.95 | 19.01% (15.72% - 22.29%) | 91.10% (88.72% - 93.48%) | 0.67 (0.61 - 0.74) |
| **UTI** | 50.00% (45.66% -54.34%) | 89.52% (86.86% -92.17%) | 4.77  (2.66 - 8.55) | 0.56  (0.33 - 0.94) | 11.86% (9.06% -14.67%) | 98.45% (97.38% -99.52%) | 0.78 (0.65 - 0.91) |
| **Abdominal** | 41.67% (34.98% -48.35%) | 88.32% (83.97% -92.68%) | 3.57  (1.65 - 7.72) | 0.66  (0.41 - 1.07) | 17.86% (12.66%-23.05%) | 96.13% (93.52% -98.75%) | 0.75 (0.61 - 0.89) |
| **SSTI** | 54.55% (46.76% -62.33%) | 86.30% (80.92% -91.68%) | 3.98  (2.03 - 7.83) | 0.53  (0.27 - 1.01) | 23.08% (16.49% -29.67%) | 96.18% (93.19% -99.18%) | 0.84 (0.72 - 0.94) |
| **Others** | 50.00% (41.60% -58.40%) | 89.84% (84.77% -94.92%) | 4.92  (2.08 - 11.68) | 0.56  (0.28 - 1.12) | 23.53% (16.40% - 30.66%) | 96.64% (93.61% -99.67%) | 0.85 (0.74 -0.95) |
| **Undefined** | 50.00% (40.25% -59.75%) | 78.65% (70.66% -86.64%) | 2.34  (1.17 - 4.68) | 0.64  (0.36 - 1.13) | 24.00% (15.67% - 32.33%) | 92.11% (86.85% -97.36%) | 0.72 (0.56 - 0.87) |
| **NEWS** | | | | | | | |
| **LRTI** | 57.4% (53.2% - 61.5%) | 71.66% (67.89% -75.44%) | 2.03  (1.56 - 2.62) | 0.59  (0.44 - 0.80) | 20.23% (16.87% -23.59%) | 93.07% (90.94% - 95.19%) | 0.71 (0.64 -0.78) |
| **UTI** | 57.1% (52.9% - 61.4%) | 87.50% (84.63% -90.37%) | 4.57  (2.75 - 7.61) | 0.49  (0.27 - 0.90) | 11.43% (8.67% -14.19% | 98.64% (97.63% - 99.64%) | 0.80 (0.69 - 0.90) |
| **Abdominal** | 41.7% (34.9% -48.4%) | 87.82% (83.38% -92.25%) | 3.42  (1.59 - 7.37) | 0.66  (0.41- 1.07) | 17.24% (12.12% - 22.36%) | 96.11% (93.49% - 98.73%) | 0.73 (0.60 - 0.89) |
| **SSTI** | 63.6% (56.1% -71.2%) | 87.67% (82.53% -92.81%) | 5.16  (2.77 - 9.61) | 0.42  (0.19 - 0.91) | 28.00% (20.98% -35.02%) | 96.97% (94.29% - 99.65%) | 0.90 (0.82 -0.97) |
| **Others** | 62.5% (54.4% -70.6%) | 90.62% (85.73% -95.52%) | 6.67  (3.12 - 14.26) | 0.41  (0.17 - 1.01) | 29.41% (21.75% - 37.07%) | 97.48% (94.84% - 100%) | 0.91 (0.84 - 0.98) |
| **Undefined** | 50.0% (40.3% -59.8%) | 78.65% (70.66% -86.64%) | 2.34  (1.17 - 4.68) | 0.64  (0.36 - 1.13) | 24.00% (15.67% - 32.33%) | 92.11% (86.85% -97.36%) | 0.78 (0.68-0.88) |
| **NEWS-2** | | | | | | | |
| **LRTI** | 59.02% (54.90% -63.13%) | 70.64% (66.82% -74.45%) | 2.01  (1.56 - 2.58) | 0.58  (0.43 - 0.79) | 20.11% (16.76%-23.47%) | 93.22% (91.12% - 95.33%) | 0.71 (0.64 - 0.77) |
| **UTI** | 57.14% (52.85% -61.44%) | 85.69% (82.65% -88.73%) | 3.99  (2.42 - 6.60) | 0.50  (0.27 - 0.92) | 10.13% (7.51%-12.74%) | 98.61% (97.59%-99.62%) | 0.79 (0.69-0.90) |
| **Abdominal** | 41.67% (34.98% -48.35%) | 87.82% (83.38% -92.25%) | 3.42  (1.59 - 7.37) | 0.66  (0.41 - 1.07) | 17.24% (12.12% -22.36%) | 96.11% (93.49% -98.73%) | 0.73 (0.57 -0.89) |
| **SSTI** | 63.64% (56.11% -71.16%) | 85.62% (80.13%-91.11%) | 4.42  (2.44 - 8.04) | 0.43  (0.19 - 0.93) | 25.00% (18.23%- 31.77%) | 96.90% (94.19%-99.61%) | 0.89 (0.81-0.96) |
| **Others** | 62.50% (54.36% -70.64%) | 90.62% (85.73% -95.52%) | 6.67  (3.12 - 14.26) | 0.41  (0.17 - 1.01) | 29.41% (21.75%-37.07%) | 97.48% (94.84%-100%) | 0.91 (0.84 -0.98) |
| **Undefined** | 50.00% (40.25%-59.75%) | 71.91% (63.15%-80.68%) | 1.78  (0.92 - 3.43) | 0.69  (0.39 - 1.24) | 19.35% (11.65%-27.06%) | 91.43% (85.97%-96.89%) | 0.74 (0.63-0.86) |
| **qSOFA** | | | | | | | |
| **LRTI** | 24.59% (20.98% -28.20%) | 90.35% (87.88% -92.82%) | 2.55  (1.52 - 4.27) | 0.84  (0.72 - 0.97) | 24.19% (20.61% 27.78%) | 90.53% (88.08% -92.99%) | 0.65 (0.58 - 0.71) |
| **UTI** | 50.00% (45.66% -54.34%) | 90.93% (88.43% -93.42%) | 5.51  (3.04 - 9.98) | 0.55  (0.33 - 0.93) | 13.46% (10.50% -16.42%) | 98.47% (97.41% -99.54%) | 0.72 (0.57-0.87) |
| **Abdominal** | 50.00% (43.22% -56.78%) | 93.91% (90.67% -97.15%) | 8.21  (3.73 - 18.05) | 0.53  (0.30 - 0.94) | 33.33% (26.94% -39.72%) | 96.86% (94.49% - 99.22%) | 0.66  (0.47 - 0.85) |
| **SSTI** | 45.45% (37.67% -53.24%) | 93.84% (90.07% - 97.60%) | 7.37  (2.98 - 18.23) | 0.58  (0.34 - 1.00) | 35.71% (28.22% -43.21%) | 95.80% (92.67% - 98.94%) | 0.77  (0.61-0.93) |
| **Others** | 12.50% (6.94% -18.06%) | 93.75% (89.68% -97.82%) | 2.00  (0.28 - 14.09) | 0.93  (0.72 - 1.22) | 11.11% (5.83% -16.39%) | 94.49% (90.65% - 98.32%) | 0.70 (0.53 - 0.88) |
| **Undefined** | 16.67% (9.40% -23.93%) | 84.27% (77.17% -91.37%) | 1.06  (0.27 - 4.10) | 0.99  (0.76 - 1.29) | 12.50% (6.05% -18.95%) | 88.24% (81.95%-94.52%) | 0.66 (0.55 - 0.78) |

Abbreviations: LRTI: Lower Respiratory Tract Infection, UTI: Urinary Tract Infection, SSTI: Skin and Soft Tissue Infection.

Cutoffs by scores: qSOFA ≥2, MEWS ≥5, NEWS and NEWS-2 ≥7.

**Table S4.** Operative characteristics for in 30 day mortality prediction.

|  | **Sensitivity (CI 95%)** | **Specificity**  **(CI 95%)** | **LR +**  **(CI 95%)** | **LR-**  **(CI 95%)** | **PPV**  **(CI 95%)** | **NPV**  **(CI 95%)** | **AUC-ROC**  **(CI 95%)** |
| --- | --- | --- | --- | --- | --- | --- | --- |
| **MEWS** | | | | | | | |
| **LRTI** | 45.45% (41.29% -49.62%) | 81.74% (78.51% -84.98%) | 2.49  (1.83 -3.40) | 0.67  (0.54 - 0.82) | 28.93% (25.13% -32.72%) | 90.16% (87.67% - 92.66%) | 0.66 (0.59 - 0.73) |
| **UTI** | 19.05% (15.64% -22.46%) | 89.10% (86.40% -91.81%) | 1.75  (0.89 - 3.43) | 0.91  (0.78 - 1.06) | 13.56% (10.59% -16.53%) | 92.46% (90.17% -94.75%) | 0.62 (0.53 - 0.69) |
| **Abdominal** | 41.18% (34.50% -47.85%) | 89.06% (84.83% -93.29%) | 3.77  (1.88 - 7.56) | 0.66  (0.44- 0.99) | 25.00% (19.13% - 30.87%) | 94.48% (91.38% - 97.57%) | 0.71 (0.57 -0.85) |
| **SSTI** | 0.00% | 82.89% (77.00% - 88.78% | 0 | 1.21  (1.12 - 1.30) | 0.00% | 96.18% (93.19% - 99.18%) | 0.52 (0.36-0.68) |
| **Others** | 9.09% (4.26% -13.92%) | 87.20% (81.59% -92.81%) | 0.71  (0.10 - 4.86) | 1.04  (0.85 - 1.27) | 5.88% (1.93% - 9.84%) | 91.60% (86.93%-96.26%) | 0.55 (0.37 -0.74) |
| **Undefined** | 37.50% (28.06% -46.94%) | 77.65% (69.52% -85.77%) | 1.68  (0.80 - 3.54) | 0.81  (0.54 - 1.20) | 24.00% (15.67% - 32.33%) | 86.84% (80.25% -93.43%) | 0.62 (0.47 - 0.77) |
| **NEWS** | | | | | | | |
| **LRTI** | 58.44% (54.32% - 62.57%) | 72.82% (69.10% -76.55%) | 2.15  (1.69 - 2.73) | 0.57  (0.44 - 0.75) | 26.01% (22.34% -29.68%) | 91.47% (89.13% -93.81%) | 0.72 (0.67 -0.79) |
| **UTI** | 23.81% (20.11% -27.51%) | 87.18% (84.28% -90.08%) | 1.86  (1.13 - 2.07) | 0.87  (0.66 - 0.98) | 14.29% (11.25% -17.32%) | 92.73% (90.47% - 94.98%) | 0.67 (0.60 - 0.75) |
| **Abdominal** | 41.18% (34.50% -47.85%) | 88.54% (84.22% -92.86%) | 3.59  (1.80 - 7.17) | 0.66  (0.44 - 0.99) | 24.14% (18.34%-29.94%) | 94.44% (91.34%- 97.55%) | 0.72 (0.56 - 0.87) |
| **SSTI** | 20.00% (13.74% -26.26%) | 84.21% (78.51% -89.91%) | 1.27  (0.21 - 7.60) | 0.95  (0.61 -1.48) | 4.00% (0.93% -7.07%) | 96.97% (94.29%-99.65%) | 0.79 (0.69 - 0.89) |
| **Others** | 18.18% (11.70% -24.66%) | 88.00% (82.54% -93.46%) | 1.51  (0.40 - 5.79) | 0.93  (0.70 - 1.24) | 11.76% (6.35%- 17.18%) | 92.44% (87.99% -96.88%) | 0.58  (0.37 - 0.79) |
| **Undefined** | 50.00% (40.25% -59.75%) | 80.00% (72.20% -87.80%) | 2.50  (1.31 - 4.78) | 0.63  (0.38 - 1.03) | 32.00% (22.90% -41.10%) | 89.47% (83.49%-95.46%) | 0.68 (0.54 - 0.82) |
| **NEWS-2** | | | | | | | |
| **LRTI** | 59.74% (55.63% -63.85%) | 71.76% (67.99% -75.53%) | 2.12  (1.68 - 2.67) | 0.56  (0.42 - 0.74) | 25.70% (22.04% -29.36%) | 91.60% (89.28%-93.92%) | 0.72 (0.66 - 0.78) |
| **UTI** | 28.57% (24.65% -32.49%) | 85.68% (82.64% -88.72%) | 1.99  (1.18 - 3.38) | 0.83  (0.69 - 1.01) | 15.19% (12.07%-18.30%) | 93.04% (90.83%-95.25%) | 0.69 (0.61 - 0.77) |
| **Abdominal** | 41.18% (34.50% -47.85%) | 88.54% (84.22% -92.86%) | 3.59  (1.80 - 7.17) | 0.66  (0.44 - 0.99) | 24.14% (18.34% -29.94%) | 94.44% (91.34% - 97.55%) | 0.71 (0.56 -0.87) |
| **SSTI** | 20.00% (13.74% -26.26%) | 82.24% (76.26% -88.22%) | 1.13  (0.19 - 6.72) | 0.97  (0.62 - 1.52) | 3.57% (0.67%-6.47%) | 96.90% (94.19% - 99.61%) | 0.77 (0.67 - 0.87) |
| **Others** | 18.18% (11.70% -24.66%) | 88.00% (82.54% -93.46%) | 1.51  (0.40 - 5.79) | 0.93  (0.70 - 1.24) | 11.76% (6.35% -17.18%) | 92.44% (87.99%- 96.88%) | 0.58 (0.37 - 0.79) |
| **Undefined** | 56.25% (46.58% -65.92%) | 74.12% (65.58% -82.66%) | 2.17  (1.24 - 3.81) | 0.59  (0.33 - 1.04) | 29.03% (20.18%- 37.88%) | 90.00% (84.15% -95.85%) | 0.69 (0.56 - 0.82) |
| **qSOFA** | | | | | | | |
| **LRTI** | 22.08% (18.61% -25.55%) | 90.45% (87.98% -92.91%) | 2.31  (1.40 - 3.82) | 0.86  (0.76 - 0.97) | 27.42% (23.68%-31.15%) | 87.65% (84.90%-90.41%) | 0.68 (0.62 -0.73) |
| **UTI** | 16.67% (13.43% -19.90%) | 90.38% (87.83% -92.94%) | 1.73  (0.83 - 3.60) | 0.92  (0.80 - 1.06) | 13.46% (10.50%-16.42%) | 92.36% (90.05%-94.66%) | 0.65 (0.57 - 0.73) |
| **Abdominal** | 23.53% (17.78% -29.28%) | 92.71% (89.18% -96.23%) | 3.23  (1.19 - 8.72) | 0.83  (0.63 - 1.08) | 22.22% (16.59%- 27.86%) | 93.19% (89.78%-96.61%) | 0.57 (0.43 - 0.71) |
| **SSTI** | 20.00% (13.74% -26.26%) | 91.45% (87.07% -95.82%) | 2.34  (0.38 - 14.56) | 0.88  (0.56 - 1.36) | 7.14% (3.11%- 11.17%) | 97.20% (94.62%-99.78%) | 0.68 (0.46 - 0.91) |
| **Others** | 9.09% (4.26% -13.92%) | 93.60% (89.49% -97.71%) | 1.42  (0.20 - 10.34) | 0.97  (0.80 - 1.18) | 11.11% (5.83%-16.39%) | 92.13% (87.60%-96.65%) | 0.69 (0.54 - 0.83) |
| **Undefined** | 43.75% (34.08% -53.42%) | 89.41% (83.41% - 95.41%) | 4.13  (1.80 - 9.48) | 0.63  (0.41 - 0.98) | 43.75% (34.08% -53.42%) | 89.41% (83.41%-95.41%) | 0.78 (0.67- 0.88) |

Abbreviations: LRTI: Lower Respiratory Tract Infection, UTI: Urinary Tract Infection, SSTI: Skin and Soft Tissue Infection.

Cutoffs by scores: qSOFA ≥2, MEWS ≥5, NEWS and NEWS-2 ≥7.

**Table S5.** Calibration of the models for in-hospital mortality.

|  | **LRTI**  **n= 548** | **UTI**  **n= 510** | **Abdominal**  **n= 209** | **SSTI**  **n= 157** | **Others**  **n= 136** | **Undefined**  **n= 101** |
| --- | --- | --- | --- | --- | --- | --- |
| MEWS |  |  |  |  |  |  |
| Number of groups | 7 | 7 | 7 | 6 | 7 | 7 |
| Hosmer-Lemeshow | 2.45 | 5.61 | 3.16 | 2.89 | 8.17 | 2.77 |
| p-Value | 0.7847 | 0.3459 | 0.6751 | 0.5766 | 0.1469 | 0.7354 |
| NEWS |  |  |  |  |  |  |
| Number of groups | 9 | 8 | 8 | 9 | 7 | 9 |
| Hosmer-Lemeshow | 3.71 | 7.04 | 4.72 | 4.58 | 2.89 | 7.20 |
| p-Value | 0.8128 | 0.3174 | 0.5802 | 0.7109 | 0.7169 | 04084 |
| NEWS-2 |  |  |  |  |  |  |
| Number of groups | 9 | 9 | 8 | 9 | 7 | 9 |
| Hosmer-Lemeshow | 3.01 | 8.92 | 4.67 | 4.83 | 3.01 | 8.51 |
| p-Value | 0.8839 | 0.2583 | 0.5872 | 0.6807 | 0.6983 | 0.2900 |
| qSOFA |  |  |  |  |  |  |
| Number of groups | 4 | 4 | 3 | 3 | 3 | 7 |
| Hosmer-Lemeshow | 2.42 | 9.83 | 2.51 | 0.99 | 7.05 | 5.67 |
| p-Value | 0.2982 | 0.0073 | 0.1131 | 0.3193 | 0.0079 | 0.3393 |

**Table S6.** Calibration of the models for ICU admission.

|  | **LRTI**  **n= 548** | **UTI**  **n= 510** | **Abdominal**  **n= 209** | **SSTI**  **n= 157** | **Others**  **n= 136** | **Undefined**  **n= 101** |
| --- | --- | --- | --- | --- | --- | --- |
| MEWS |  |  |  |  |  |  |
| Number of groups | 7 | 7 | 7 | 7 | 7 | 7 |
| Hosmer-Lemeshow | 5.98 | 2.63 | 2.04 | 6.82 | 1.71 | 4.81 |
| p-Value | 0.3086 | 0.7571 | 0.8434 | 0.2347 | 0.8880 | 0.4397 |
| NEWS |  |  |  |  |  |  |
| Number of groups | 9 | 8 | 8 | 9 | 7 | 9 |
| Hosmer-Lemeshow | 7.36 | 5.65 | 4.92 | 2.02 | 3.23 | 7.55 |
| p-Value | 0.3927 | 0.4641 | 0.5544 | 0.9589 | 0.6645 | 0.3738 |
| NEWS-2 |  |  |  |  |  |  |
| Number of groups | 9 | 8 | 9 | 7 | 9 | 4 |
| Hosmer-Lemeshow | 7.01 | 4.99 | 2.29 | 3.56 | 8.46 | 0.55 |
| p-Value | 0.4283 | 0.5448 | 0.9422 | 0.6139 | 0.2936 | 0.7588 |
| qSOFA |  |  |  |  |  |  |
| Number of groups | 4 | 4 | 3 | 3 | 3 | 4 |
| Hosmer-Lemeshow | 0.55 | 6.33 | 6.36 | 0.39 | 2.06 | 6.22 |
| p-Value | 0.7588 | 0.423 | 0.0117 | 0.5318 | 0.1508 | 0.0447 |

**Table S7.** Calibration of the models for 30-day mortality.

|  | **LRTI**  **n= 548** | **UTI**  **n= 510** | **Abdominal**  **n= 209** | **SSTI**  **n= 157** | **Others**  **n= 136** | **Undefined**  **n= 101** |
| --- | --- | --- | --- | --- | --- | --- |
| MEWS |  |  |  |  |  |  |
| Number of groups | 7 | 7 | 7 | 6 | 7 | 7 |
| Hosmer-Lemeshow | 5.67 | 2.61 | 0.87 | 4.18 | 6.06 | 4.35 |
| p-Value | 0.3393 | 0.7594 | 0.9720 | 0.3818 | 0.3005 | 0.5000 |
| NEWS |  |  |  |  |  |  |
| Number of groups | 9 | 8 | 8 | 9 | 7 | 9 |
| Hosmer-Lemeshow | 4.31 | 9.12 | 5.11 | 14.27 | 5.59 | 9.64 |
| p-Value | 0.7430 | 0.1670 | 0.5293 | 0.0465 | 0.3487 | 0.2101 |
| NEWS-2 |  |  |  |  |  |  |
| Number of groups | 9 | 9 | 8 | 9 | 7 | 9 |
| Hosmer-Lemeshow | 4.47 | 9.20 | 4.94 | 14.99 | 6.12 | 11.13 |
| p-Value | 0.7240 | 0.2387 | 0.5515 | 0.0361 | 0.2943 | 0.1330 |
| qSOFA |  |  |  |  |  |  |
| Number of groups | 4 | 4 | 3 | 3 | 3 | 4 |
| Hosmer-Lemeshow | 5.59 | 4.60 | 1.44 | 0.44 | 3.42 | 3.08 |
| p-Value | 0.0610 | 0.1005 | 0.2295 | 0.5068 | 0.0643 | 0.2142 |

**Calibration curves**

**ICU**

A)

B)

C)

D)

E)

F)

**Figure S1.** Calibration curves for ICU admission-MEWS A) LRTI B) UTI C) Abdominal D) SSTI E) Others F) Undefined.

A)

B)

C)

D)

**Figure S2.** Calibration for ICU admission-NEWS A) LRTI B) SSTI C) Others D) Undefined.

A)

B)

C)

D)

E)

F)

**Figure S3.** Calibration curves for ICU admission-NEWS2 A) LRTI B) UTI C) Abdominal D) SSTI E) Others F) Undefined.

A)

B)

**Figure S4.** Calibration curves for ICU admission-qSOFA A) LRTI B) SSTI.

**In-hospital mortality**

A)

B)

**Figure S5.** Calibration curves for In-hospital mortality-MEWS A) LRTI B) Abdominal

A)

B)

C)

**Figure S6.** Calibration curves for In-hospital mortality-NEWS A) LRTI B) Abdominal C) Undefined

A)

B)

C)

D)

**Figure S7.** Calibration curves for In-hospital mortality-NEWS2 A) LRTI B) Abdominal C) Others D) Undefined

A)

B)

**Figure S8.** Calibration curves for In-hospital mortality-qSOFA A) LRTI B) Undefined

**30-Day mortality**

A)

B)

**Figure S9.** Calibration curves for 30-day mortality-MEWS A) LRTI B) Abdominal

A)

B)

C)

**Figure S10.** Calibration curves for 30-day mortality-NEWS A) LRTI B) Abdominal C) Undefined

A)

B)

C)

D)

**Figure S11.** Calibration curves for 30-day mortality-NEWS2 A) LRTI B) UTI C) Abdominal D) Undefined

A)

B)

**Figure S12.** Calibration curves for 30-day mortality-qSOFA A) LRTI B) Undefined

**Worst case scenario**

**Inhospital mortality**


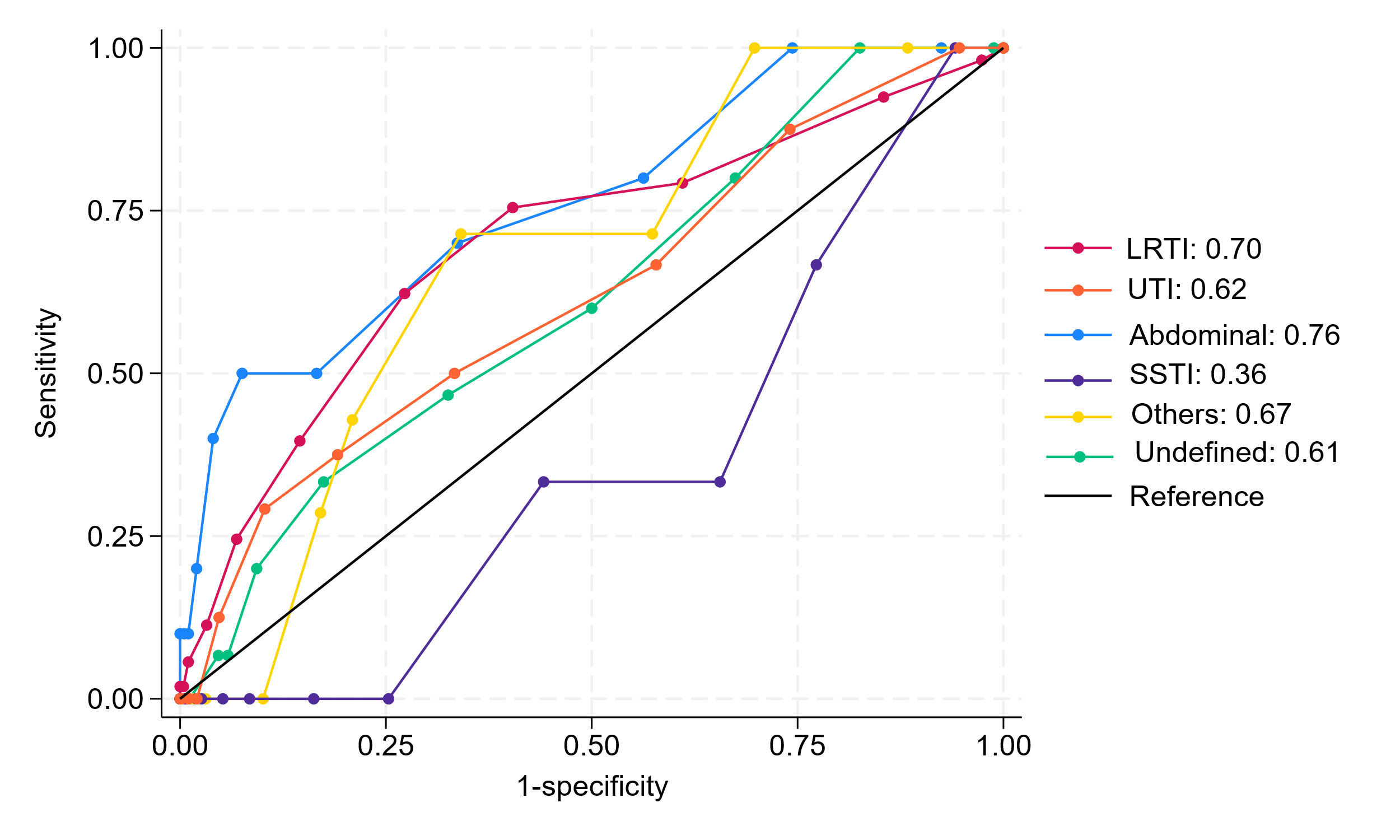

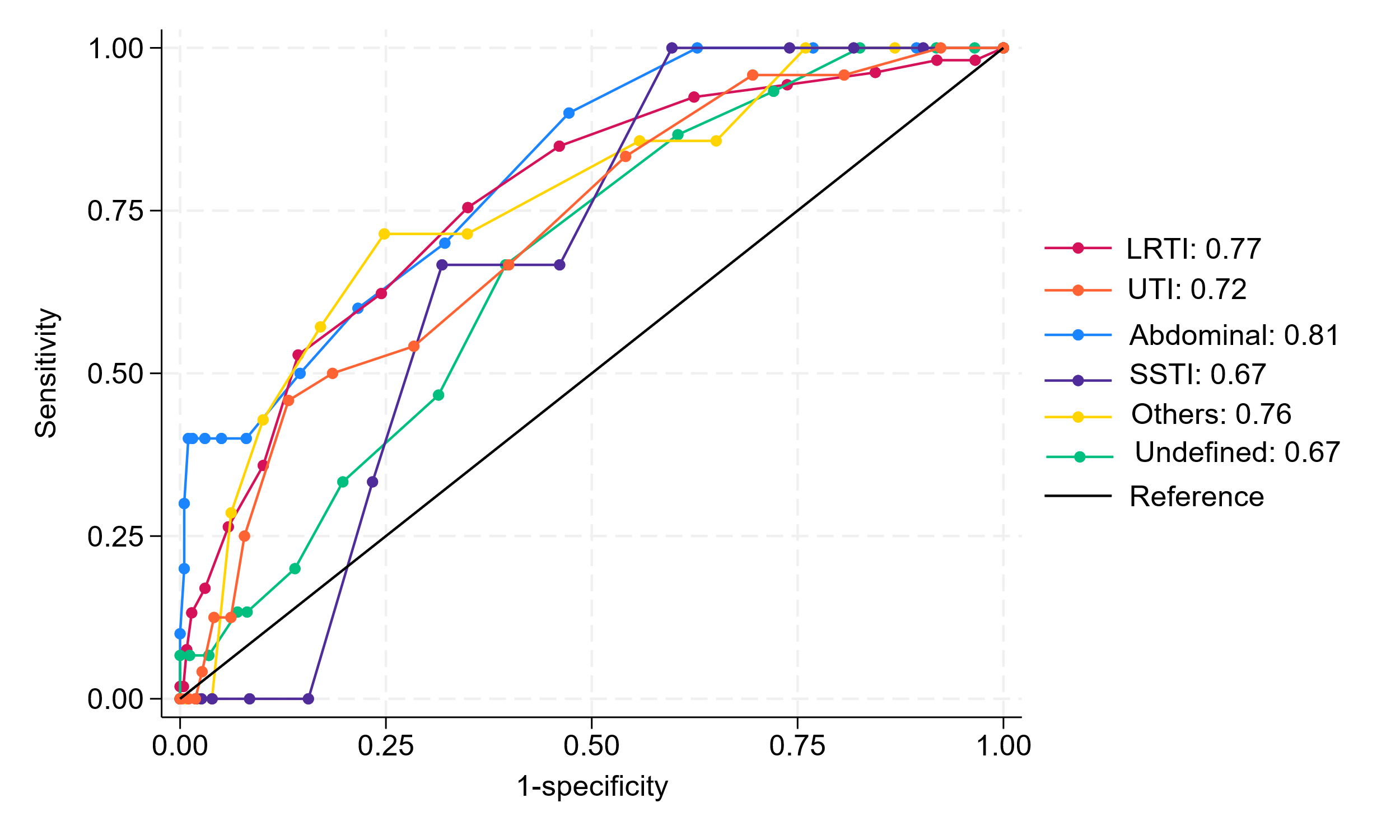

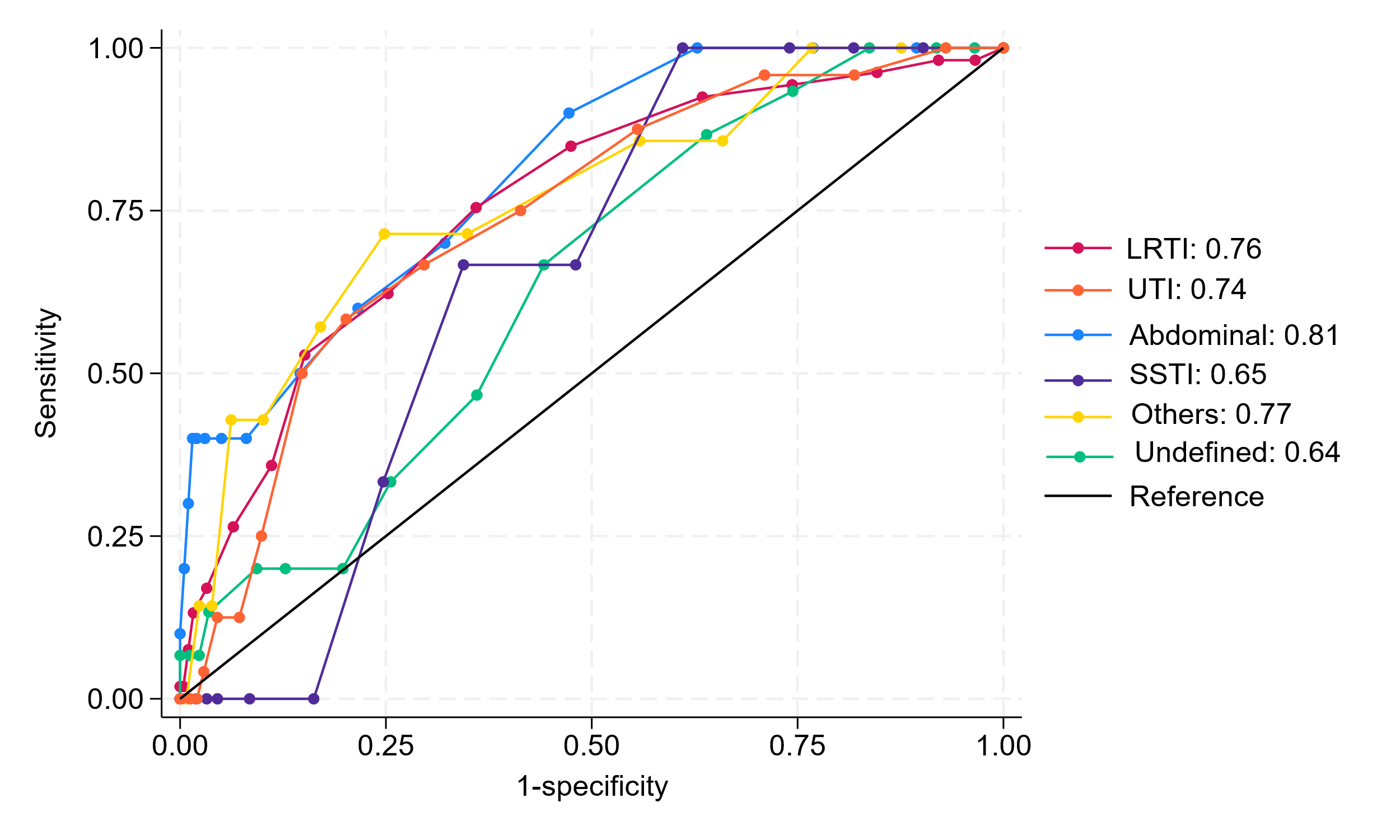

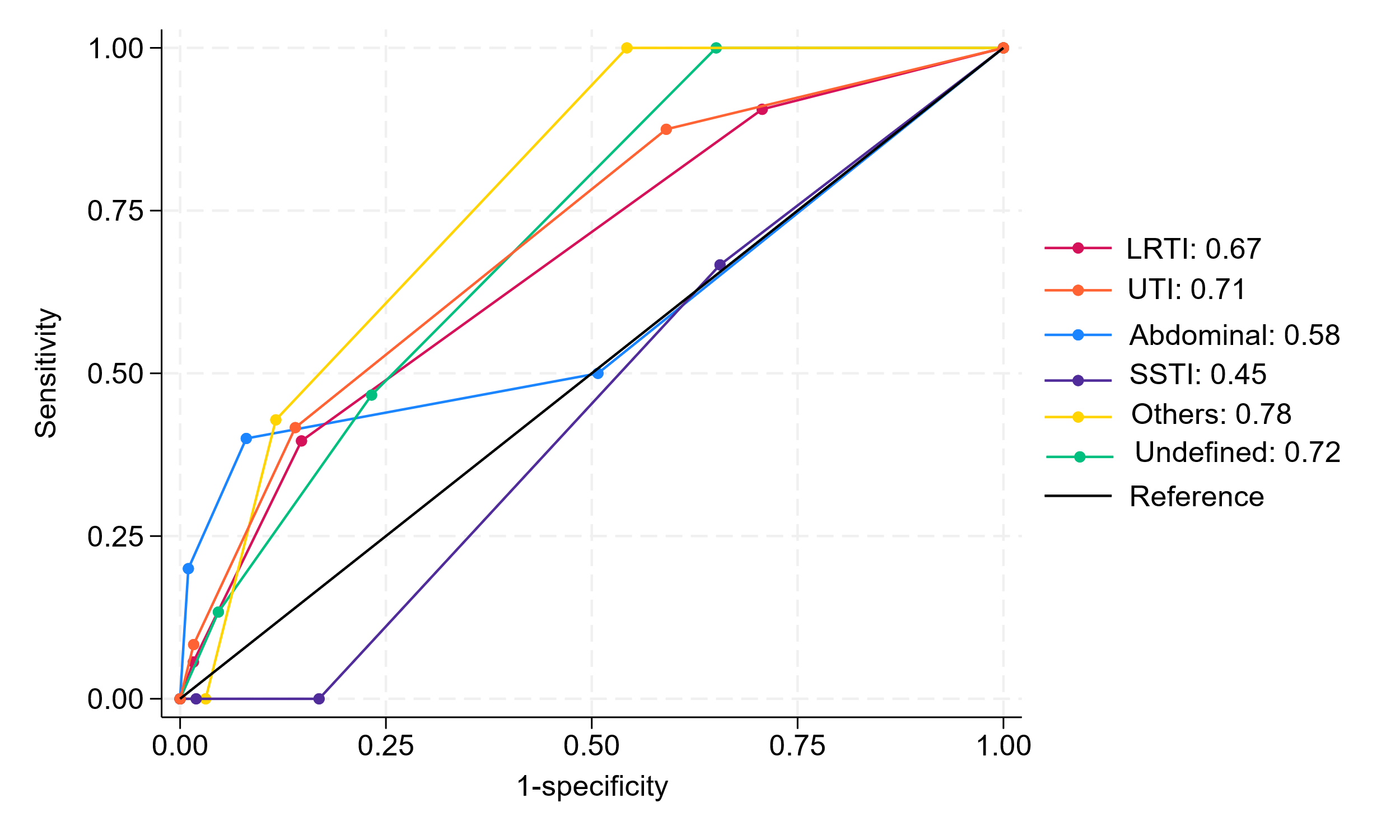


A)

B)

C)

D)

**Figure S13.** Worst case scenario. Receiver operating characteristic curve in the different subcohorts for EWS in the discrimination of inhospital mortality. A) MEWS B) NEWS C) NEWS-2 D) qSOFA.

**ICU**


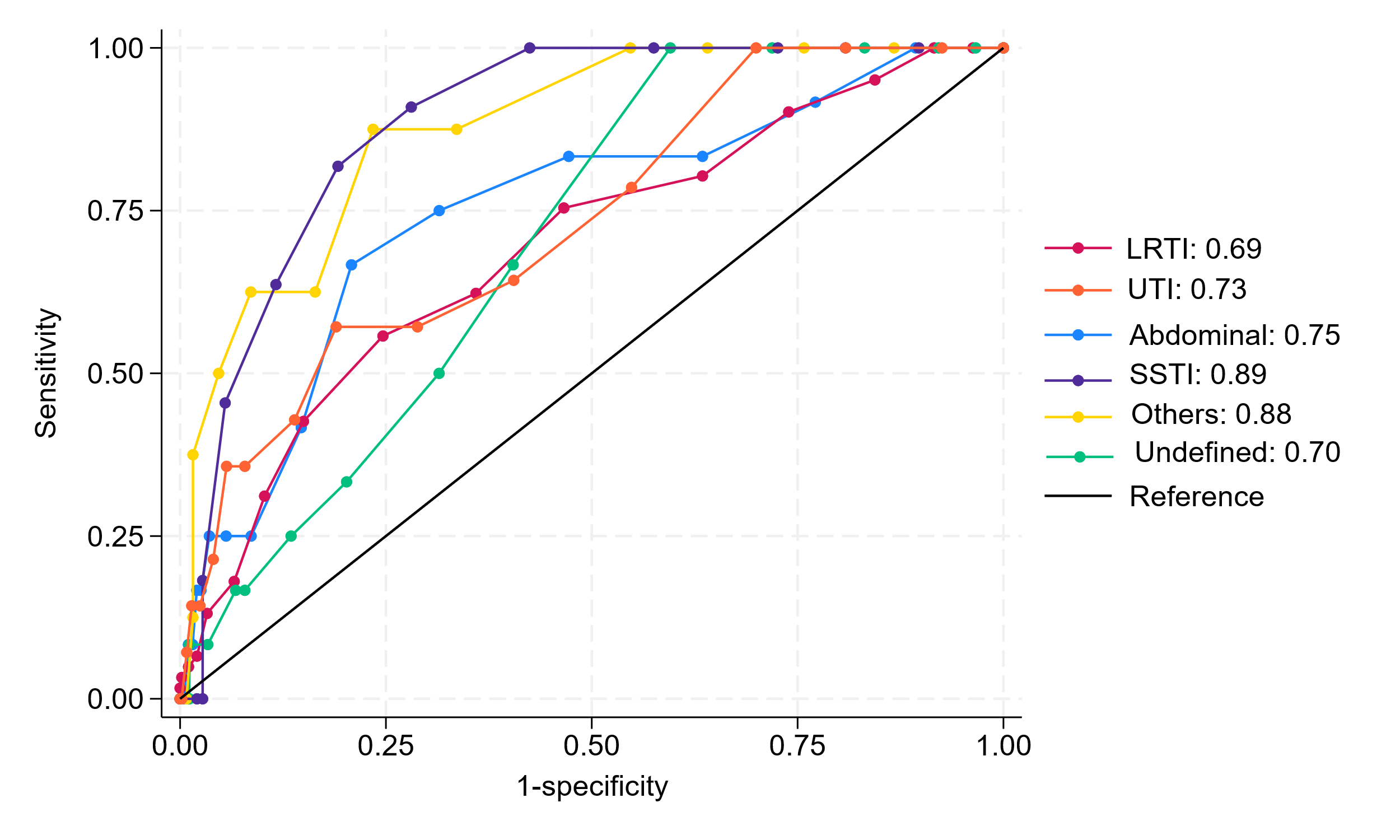

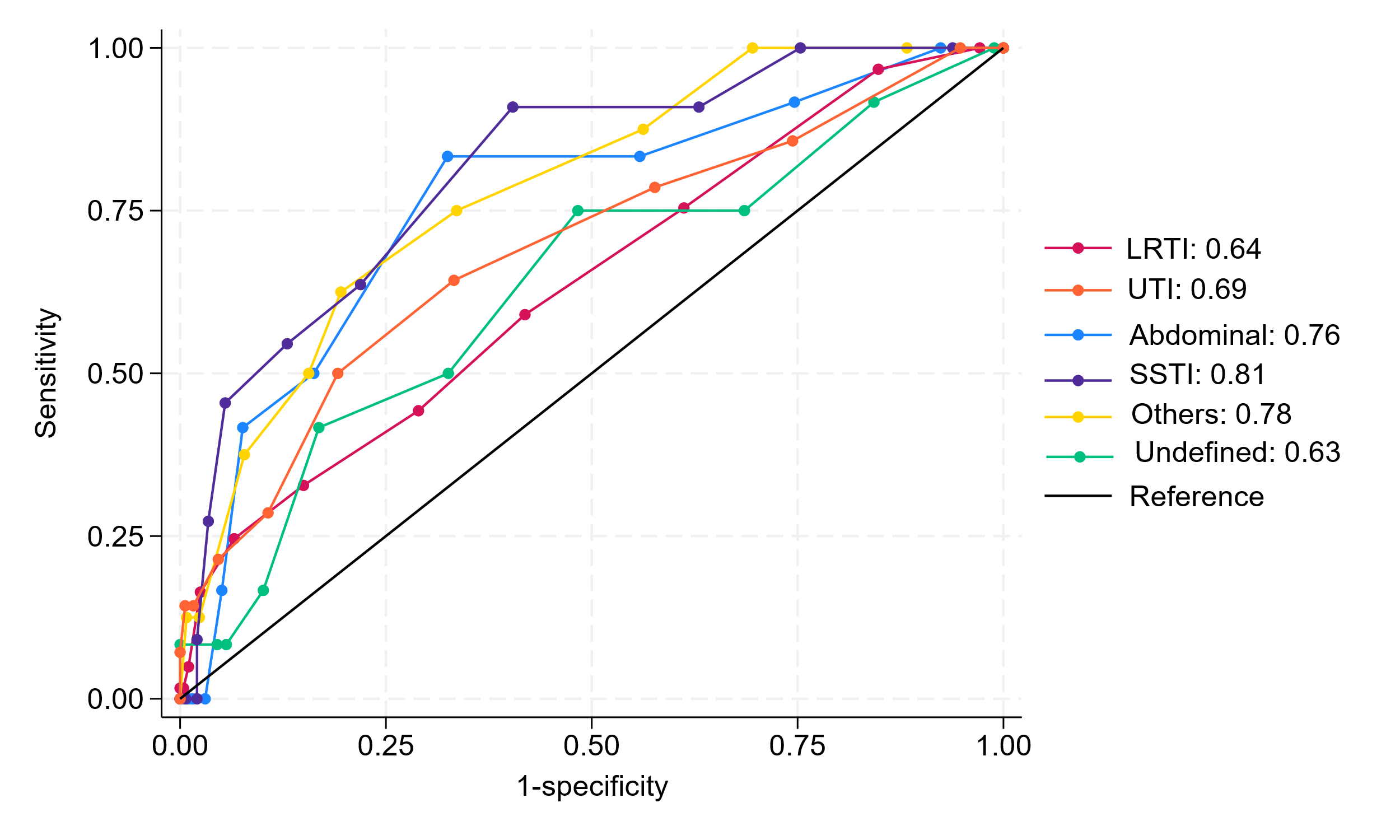

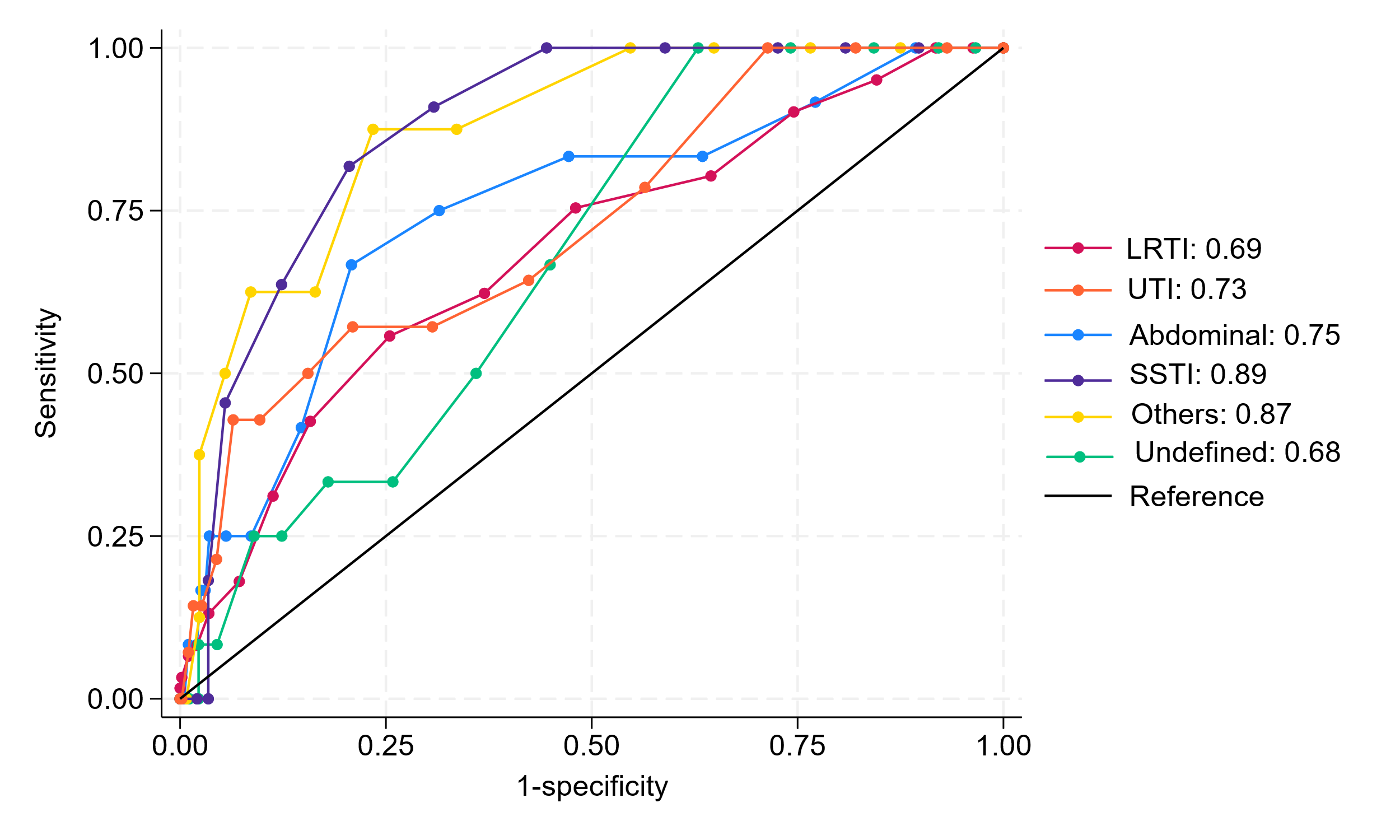

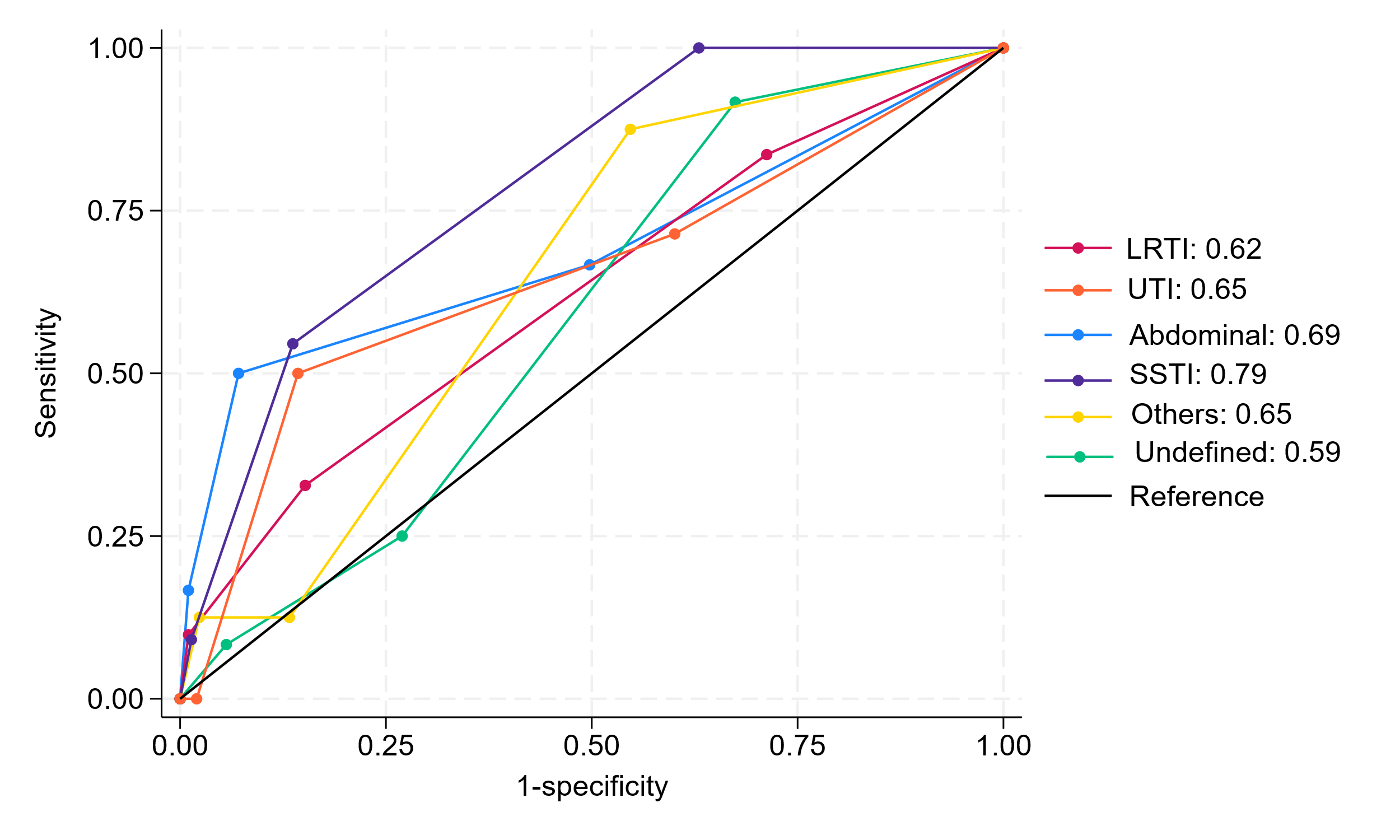


A)

B)

C)

D)

**Figure S14.** Worst case scenario. Receiver operating characteristic curve in the different subcohorts for EWS in the discrimination of ICU admission. A) MEWS B) NEWS C) NEWS-2 D) qSOFA.

**30-Day mort**


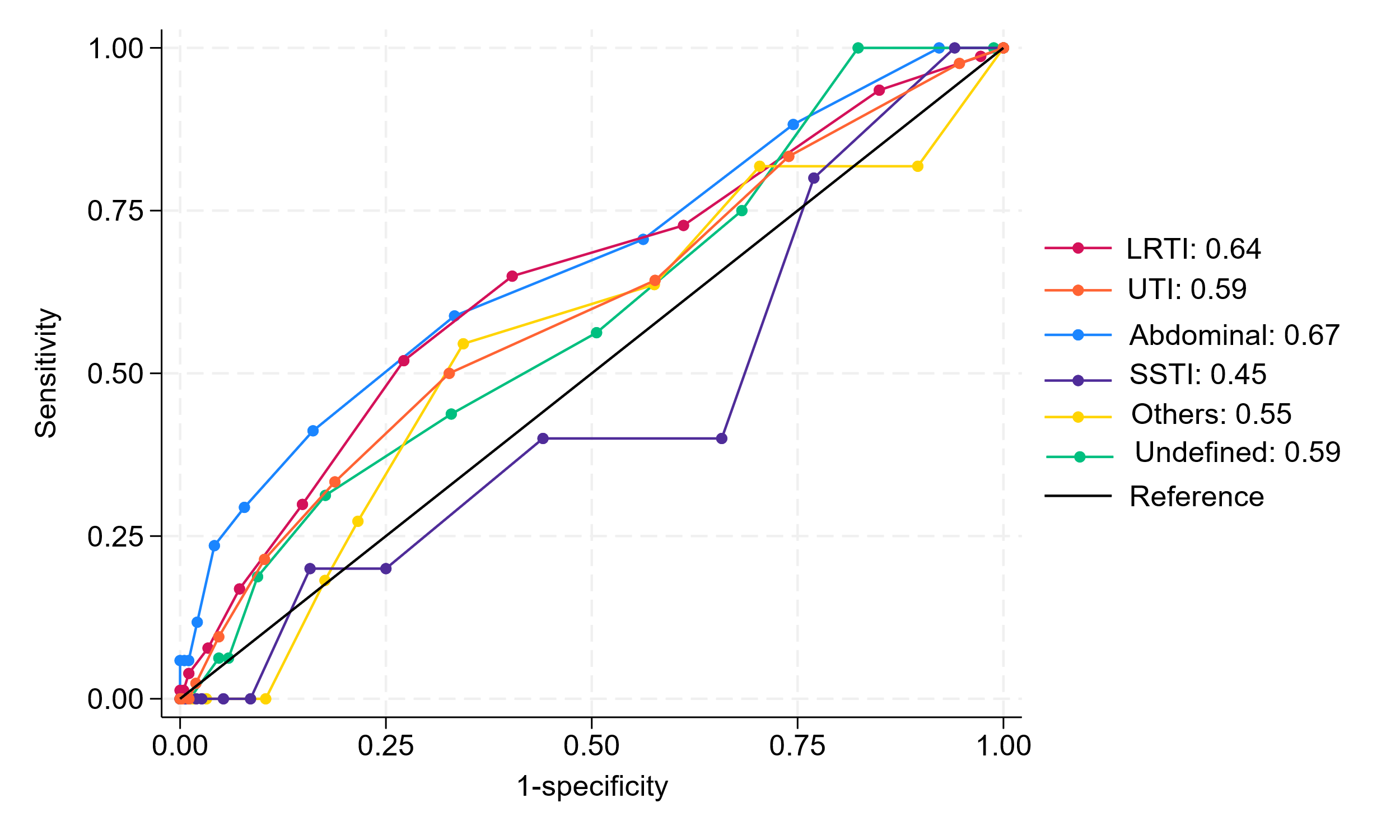

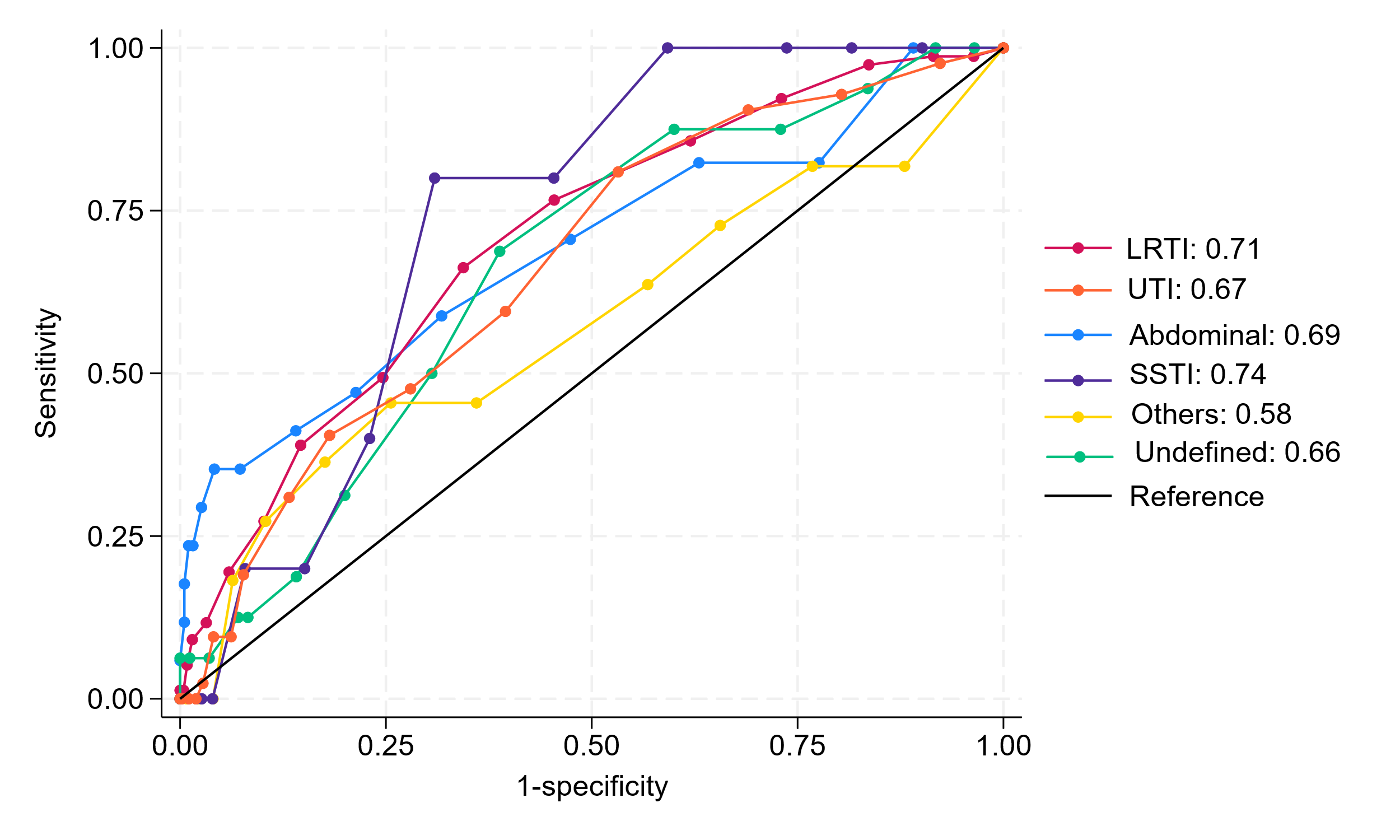

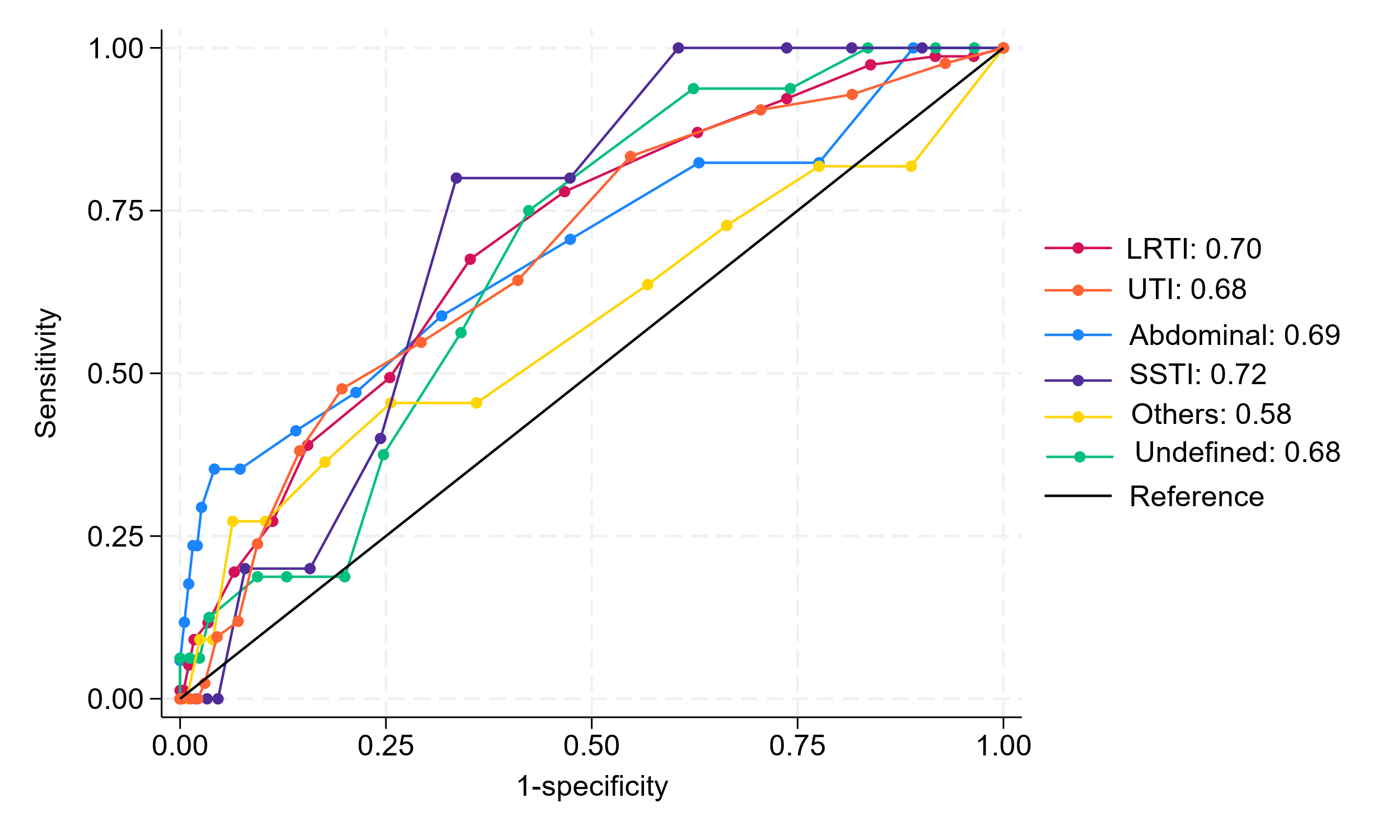

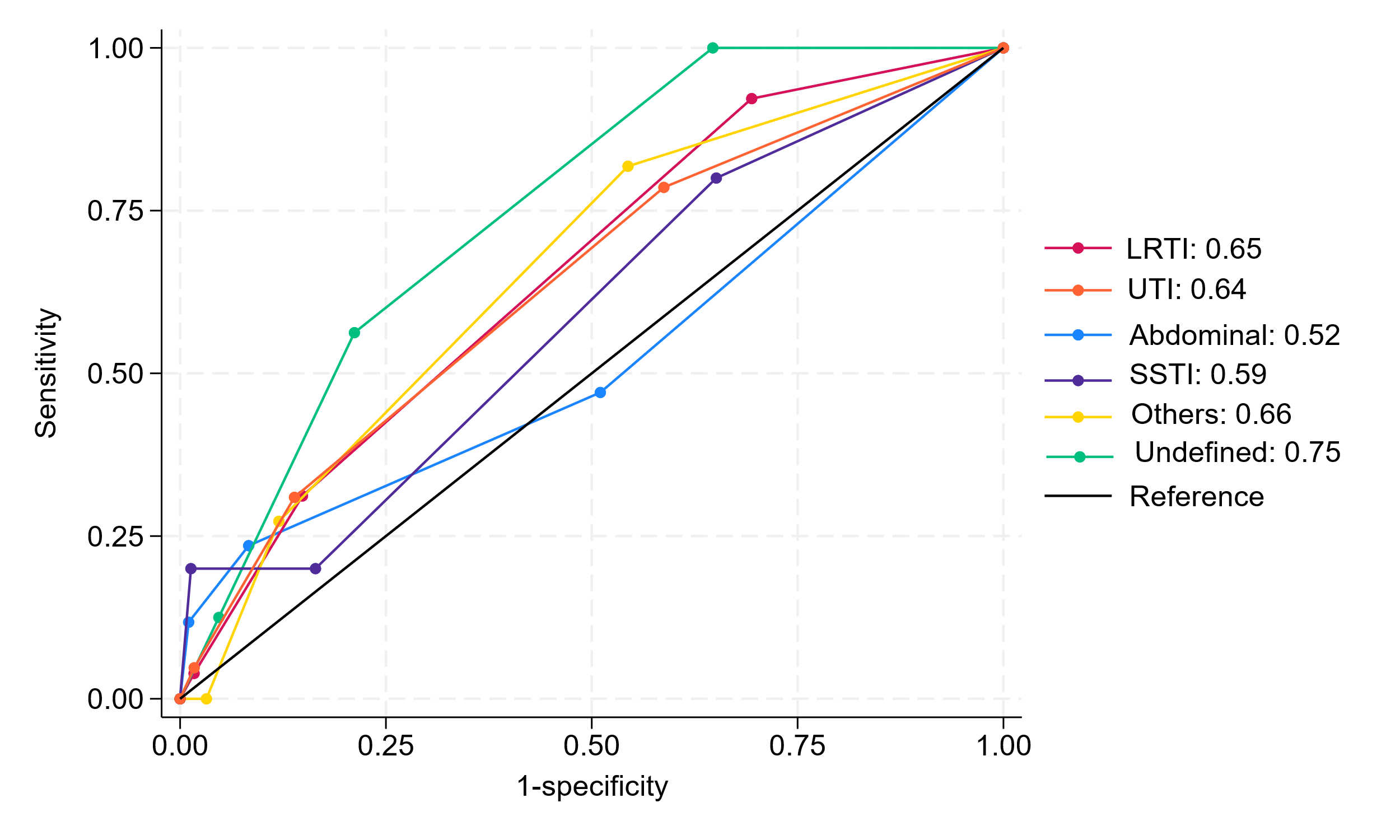


A)

B)

C)

D)

**Figure S15.** Worst case scenario. Receiver operating characteristic curve in the different subcohorts for EWS in the discrimination of 30-day mortality. A) MEWS B) NEWS C) NEWS-2 D) qSOFA.

**Imputed**

**In-hospital mortality**


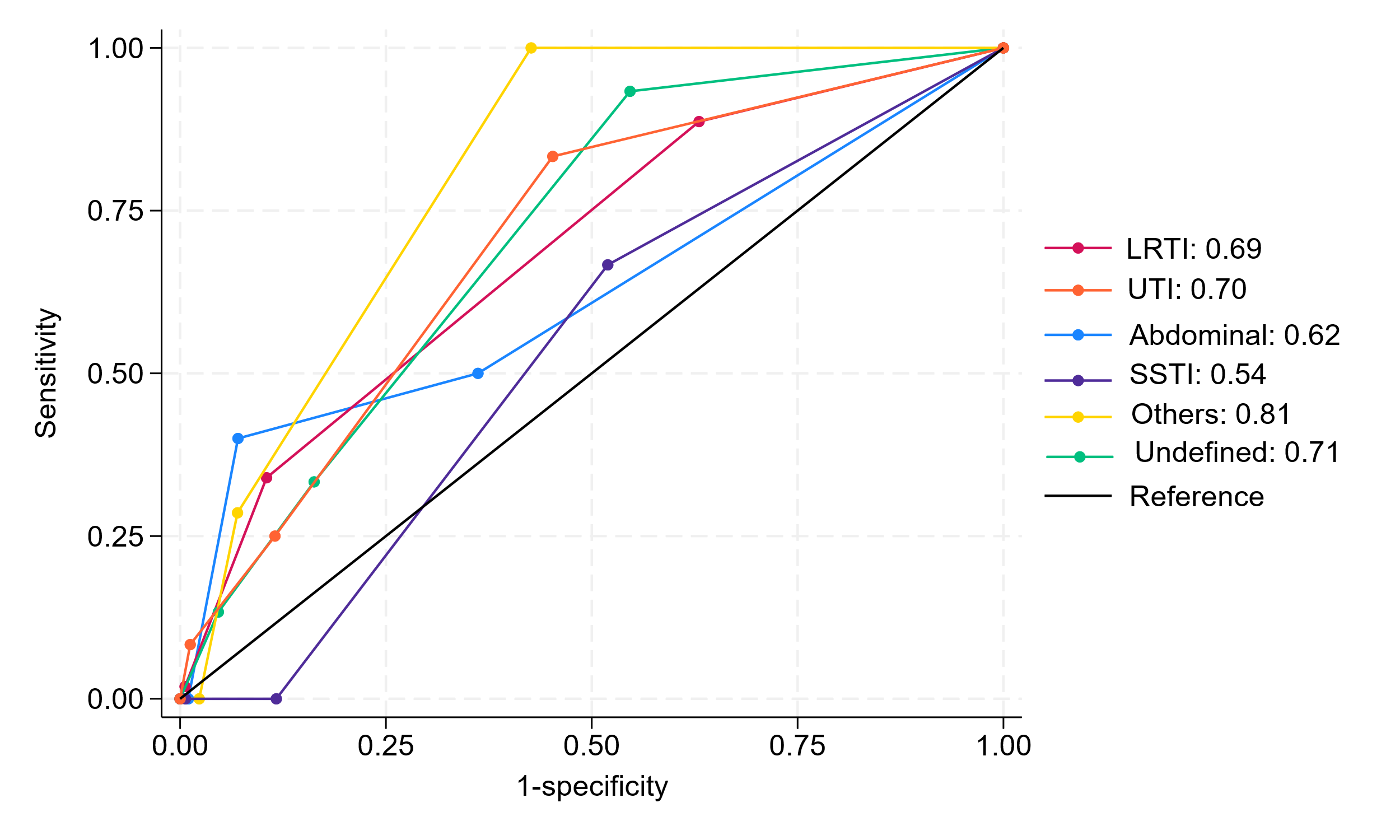

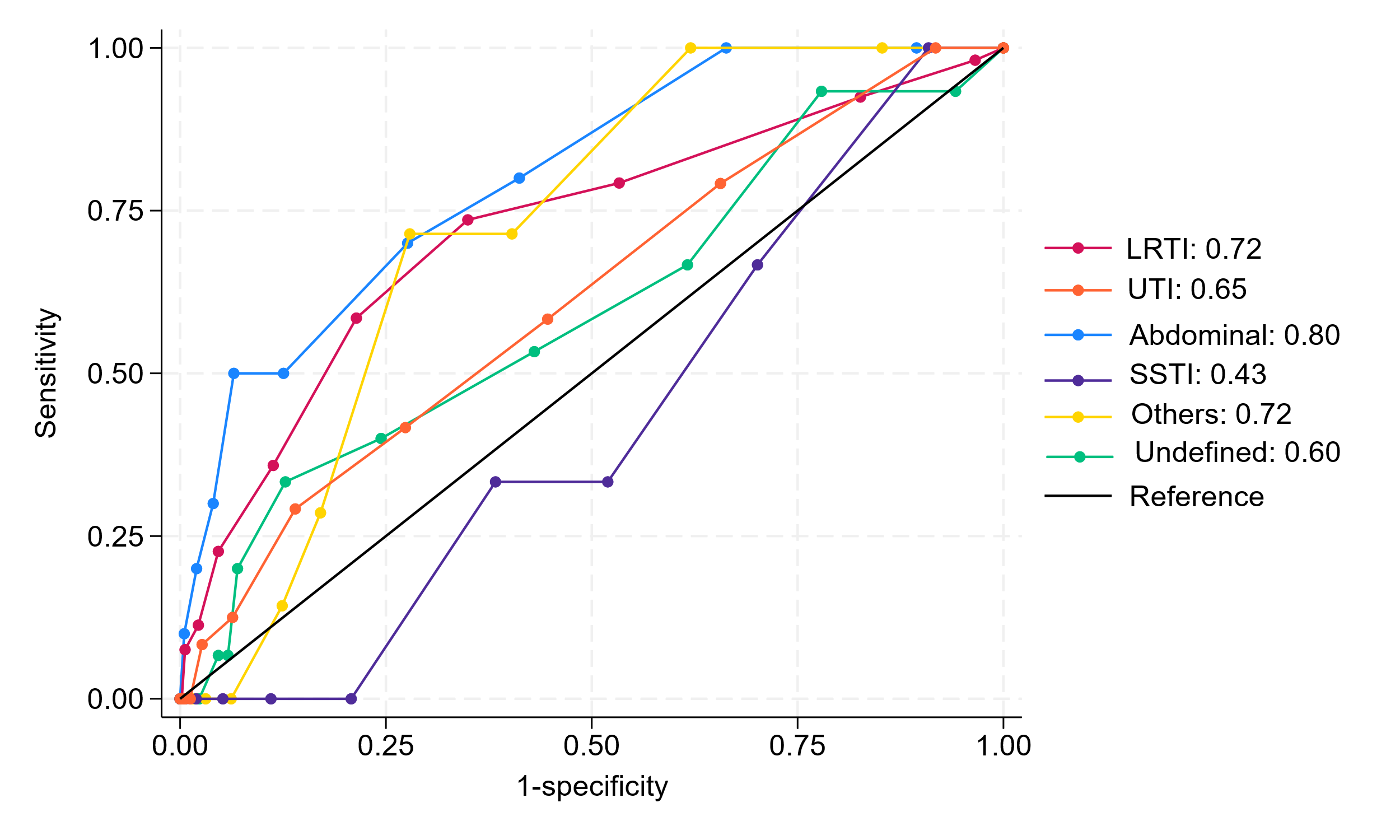

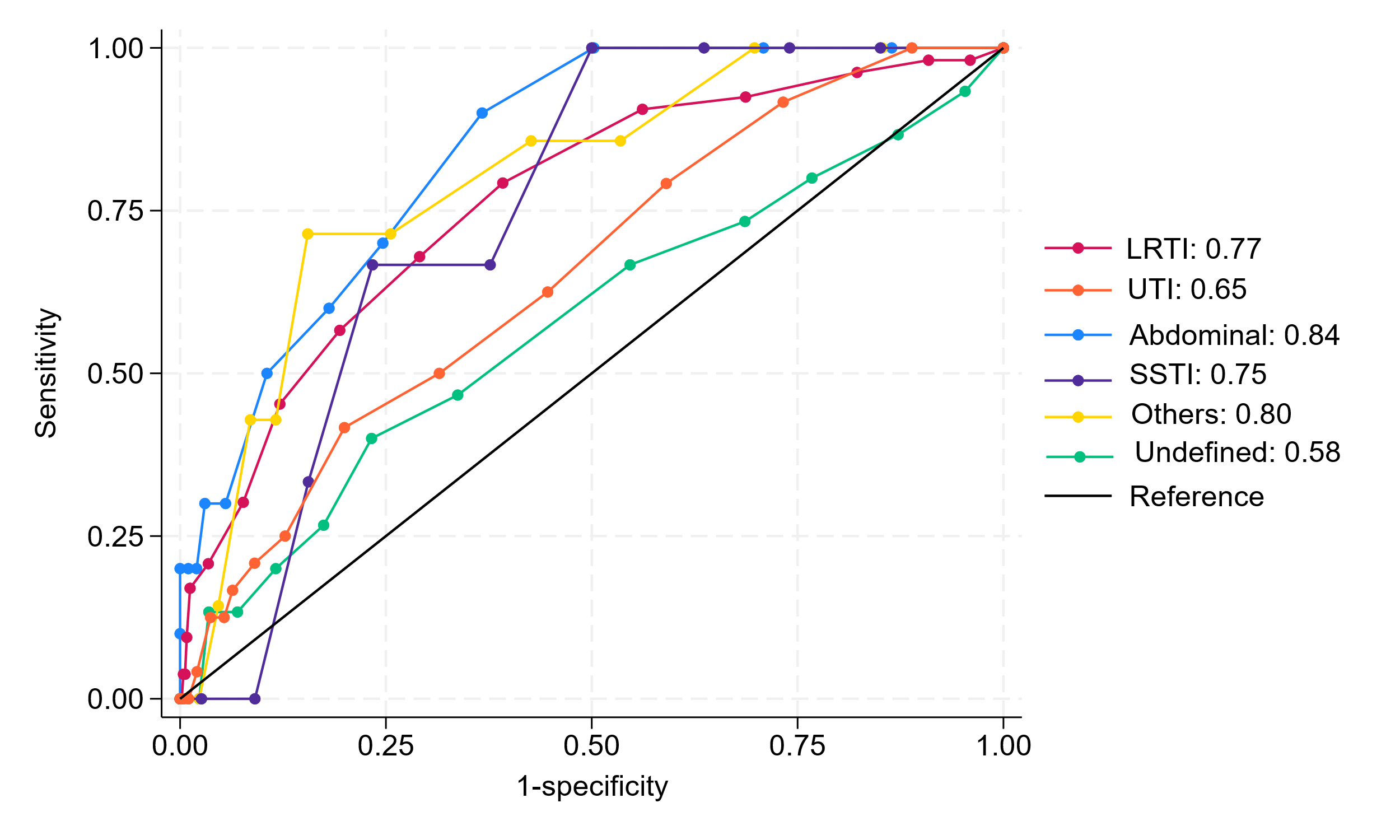

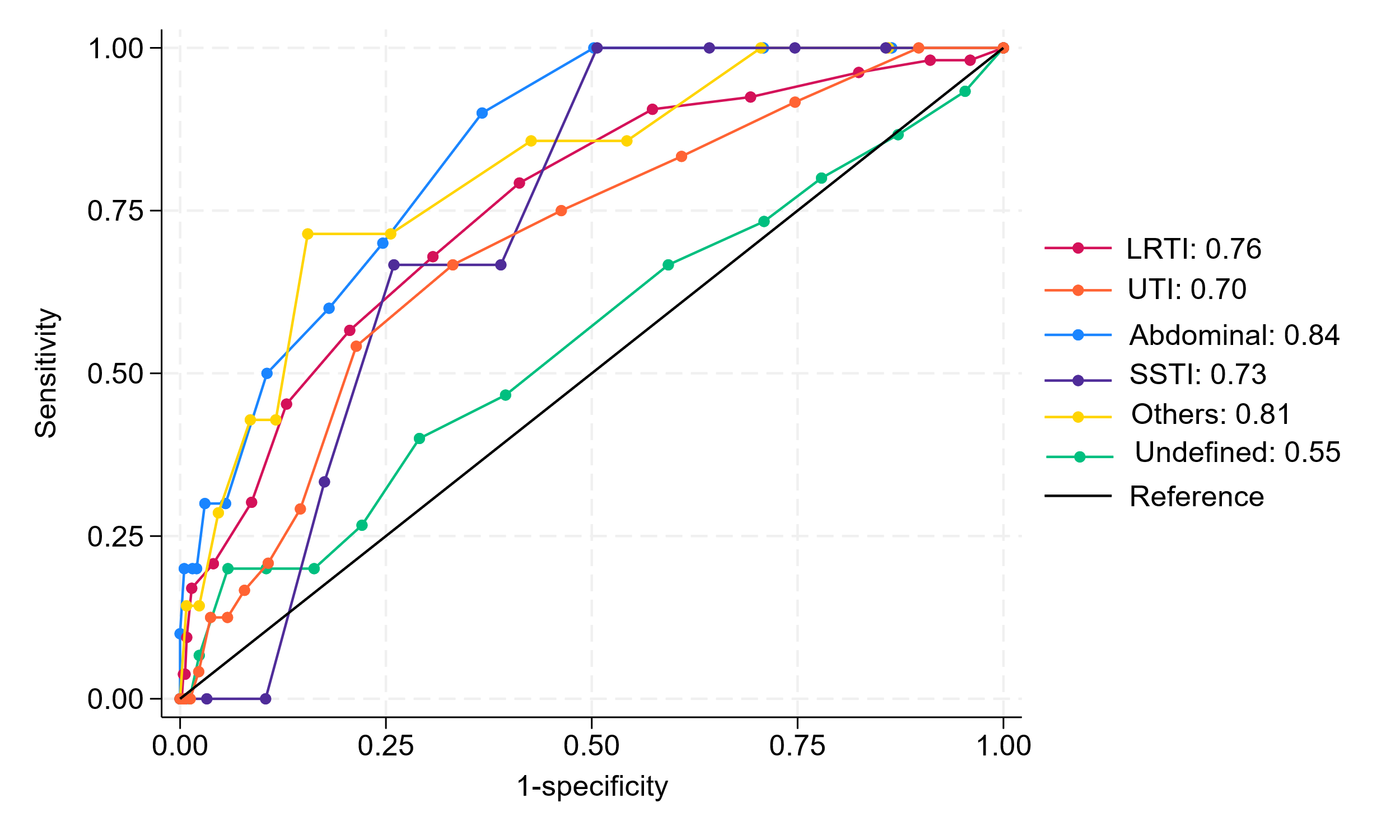


A)

B)

C)

D)

**Figure S16.** Imputed data. Receiver operating characteristic curve in the different subcohorts for EWS in the discrimination of inhospital mortality. A) MEWS B) NEWS C) NEWS-2 D) qSOFA.

**ICU**


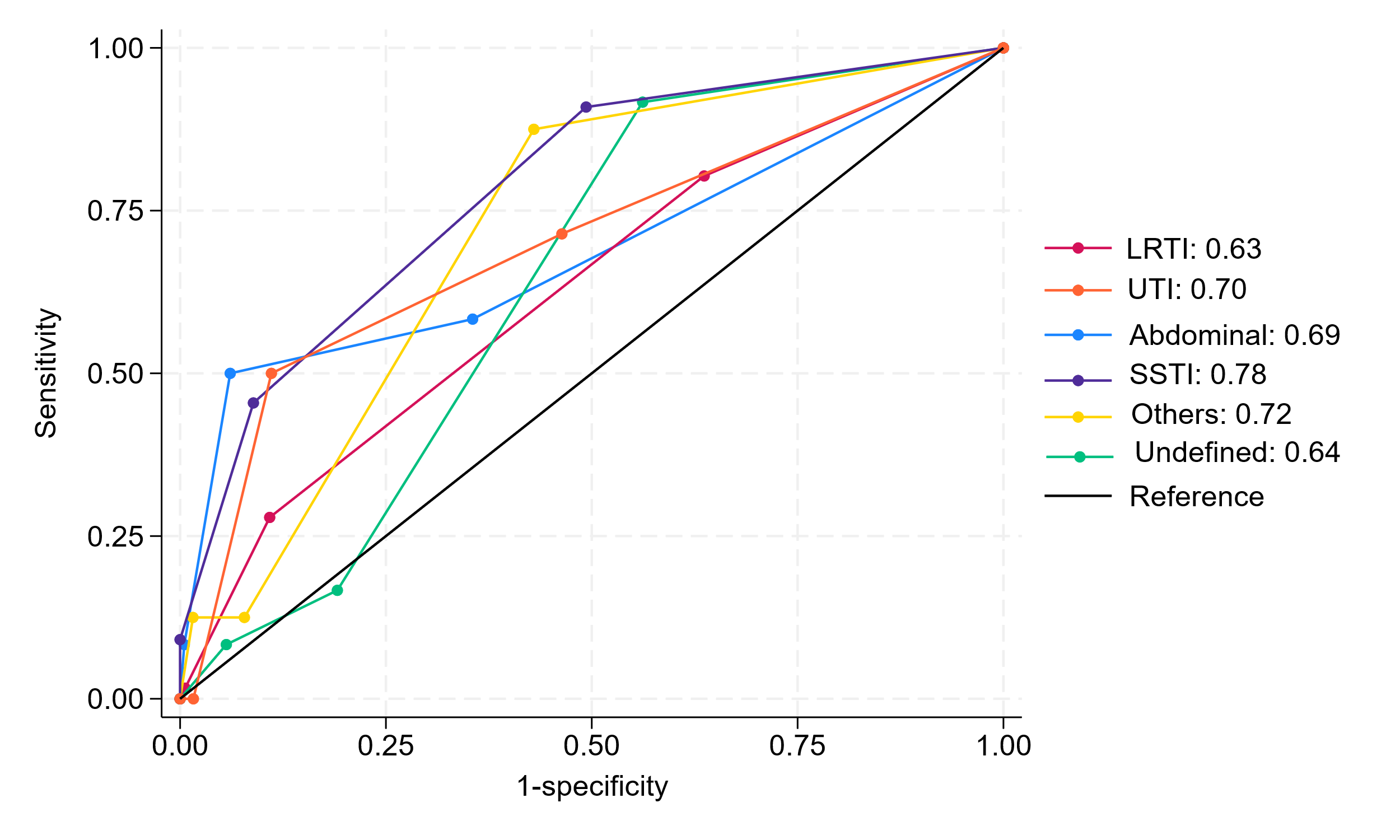

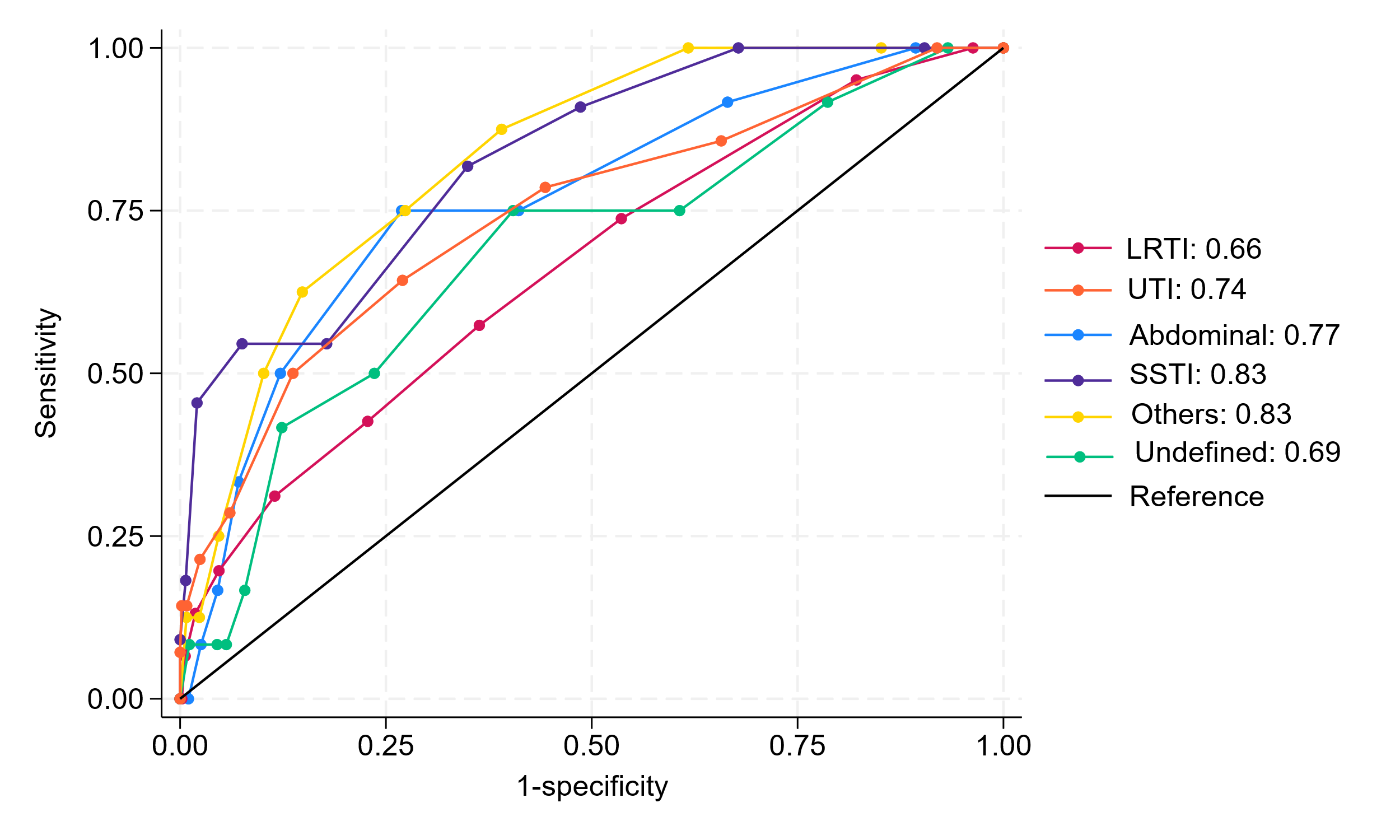

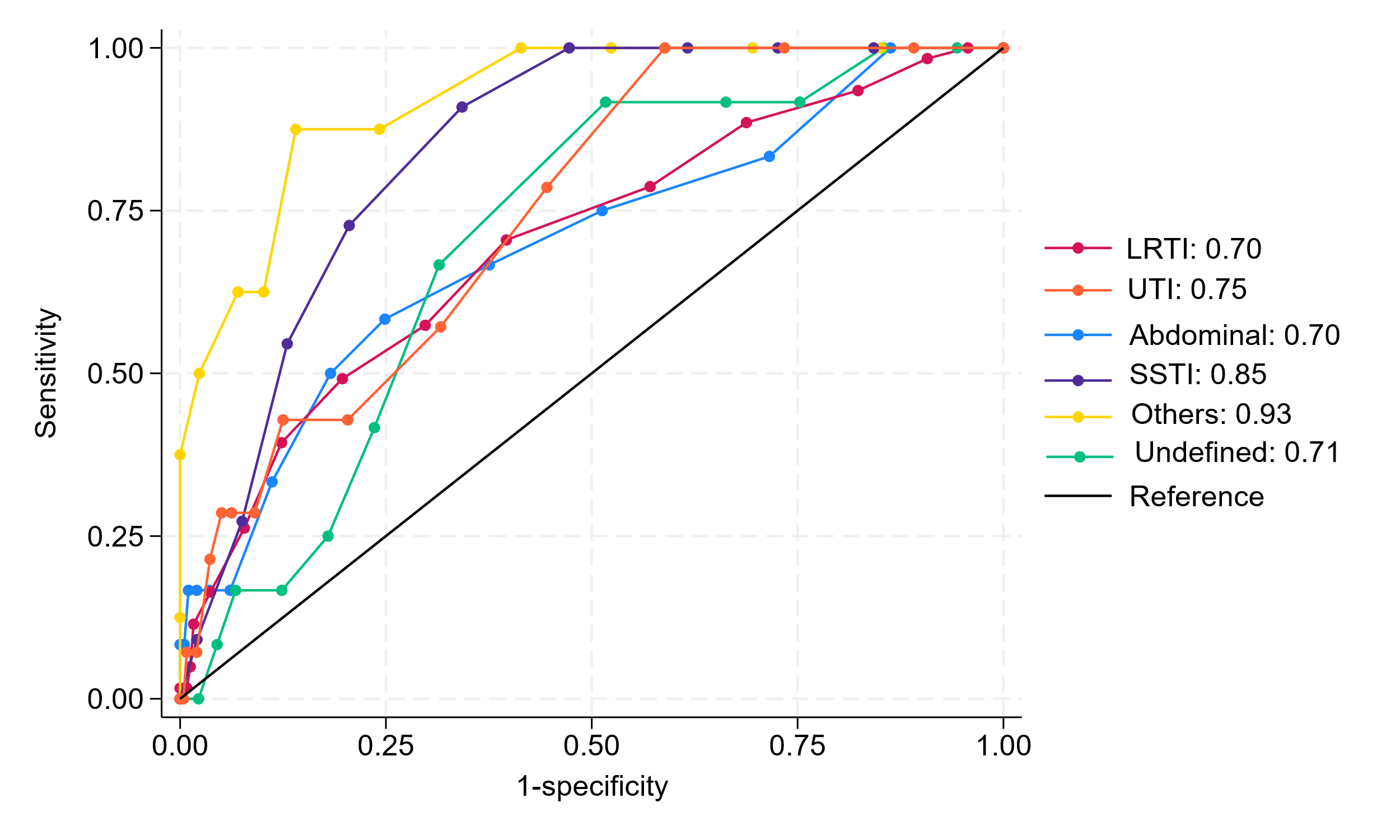

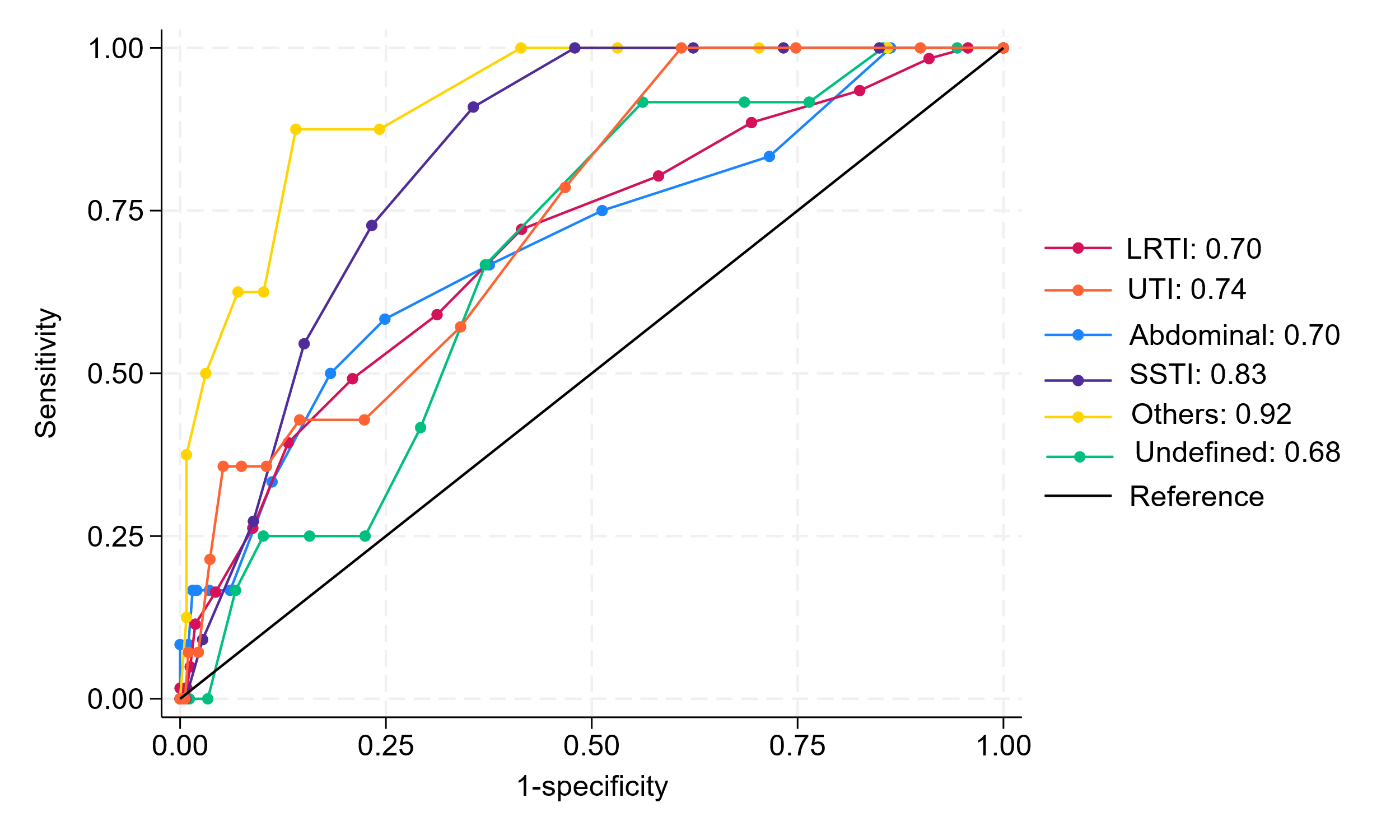


A)

B)

C)

D)

**Figure S17.** Imputed data. Receiver operating characteristic curve in the different subcohorts for EWS in the discrimination of ICU admission. A) MEWS B) NEWS C) NEWS-2 D) qSOFA.

**30-Day mort**


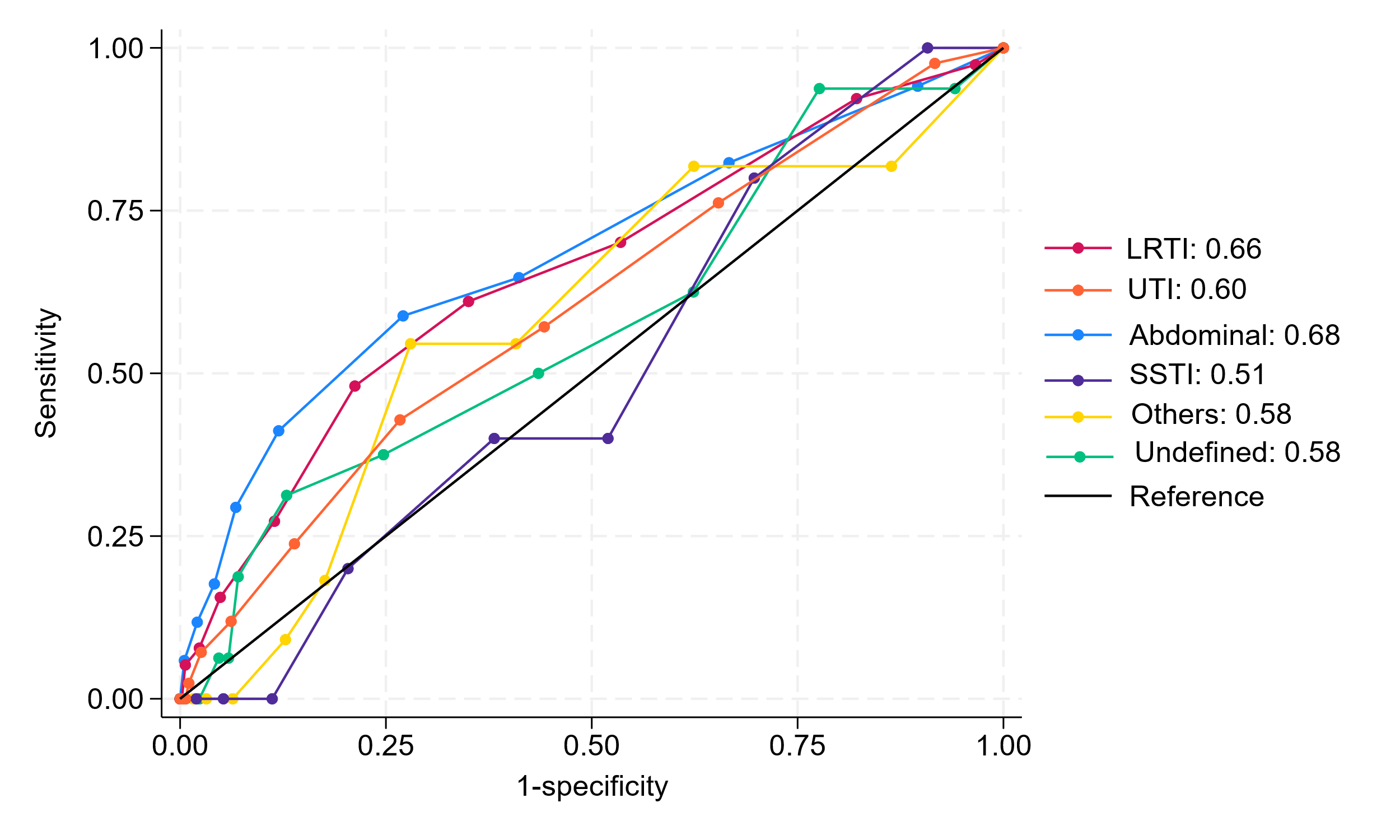

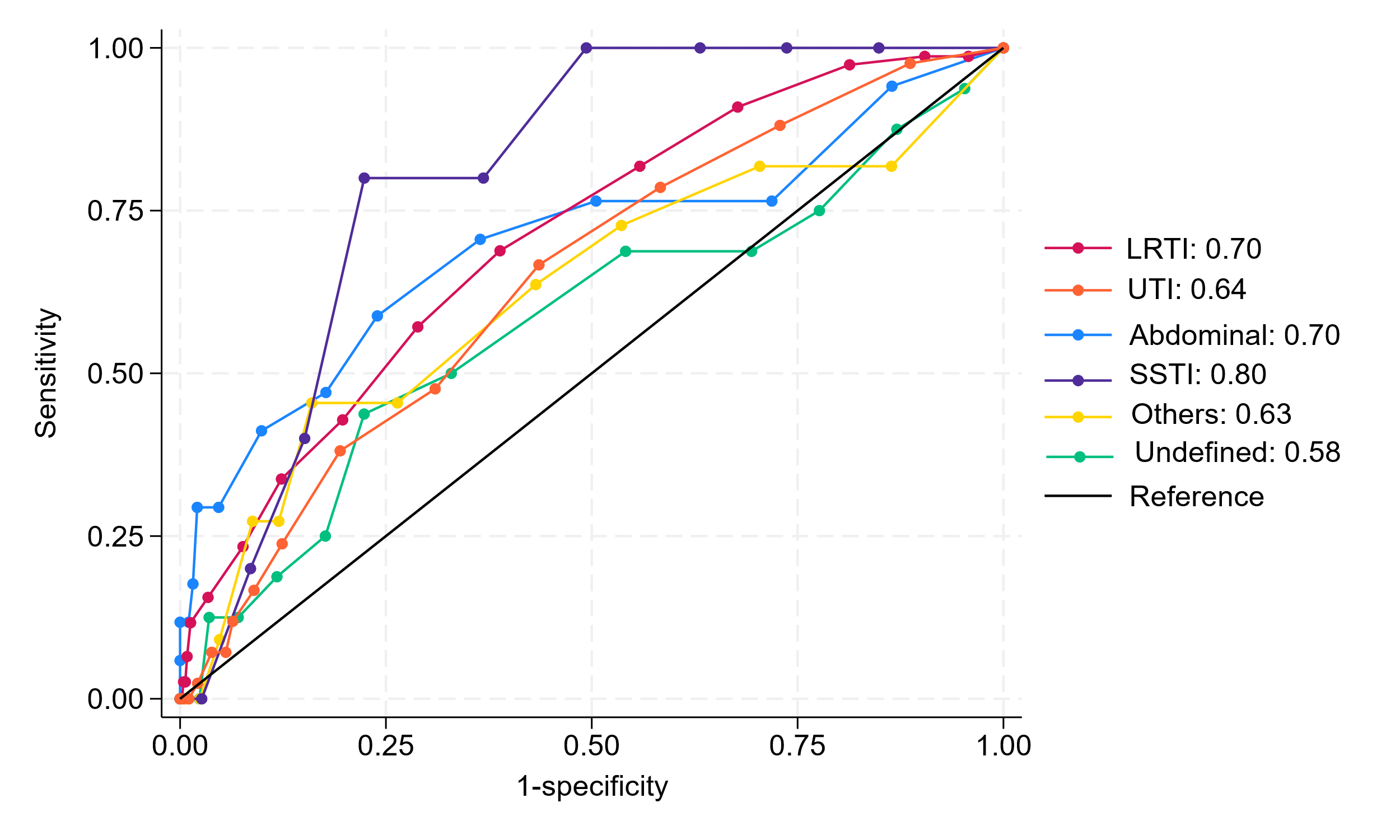

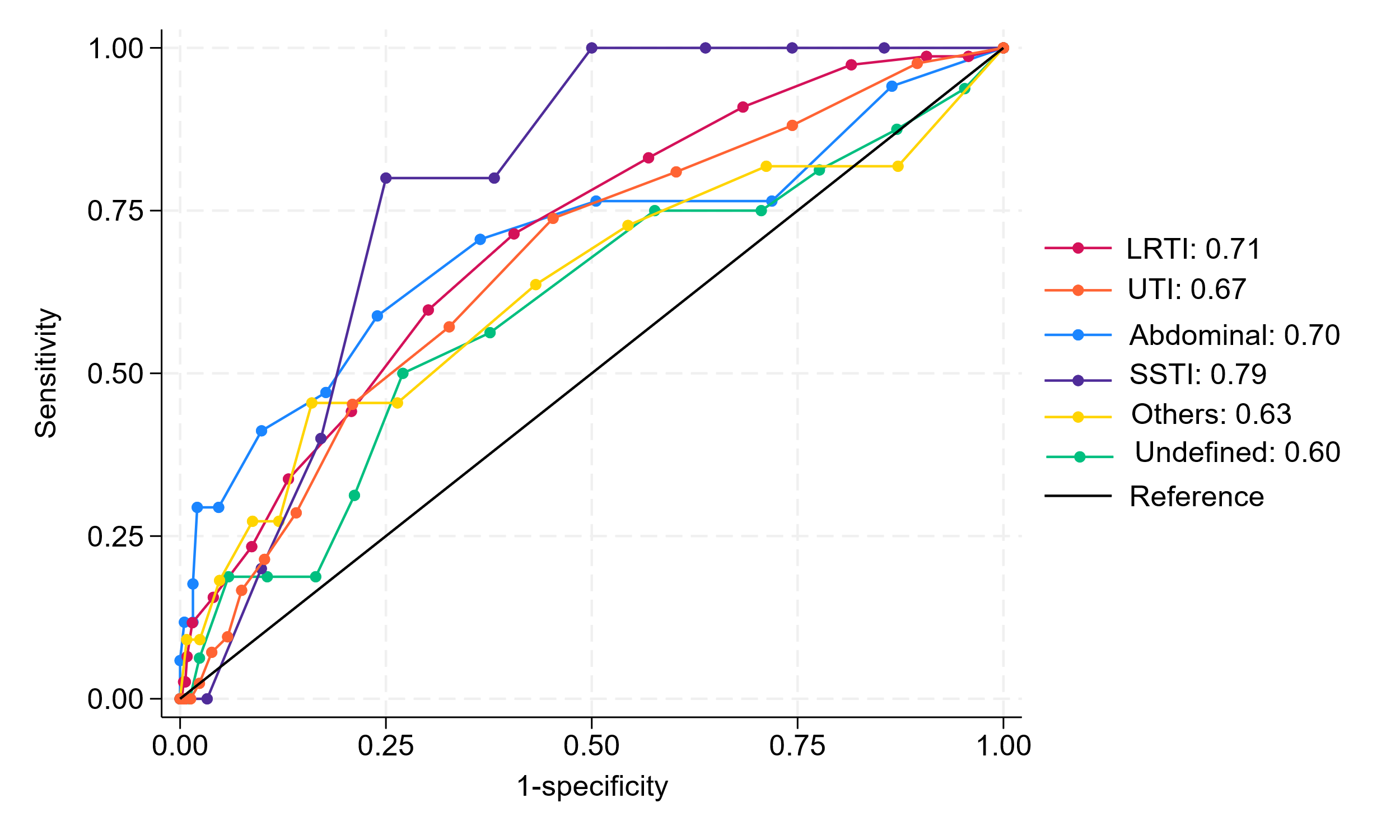

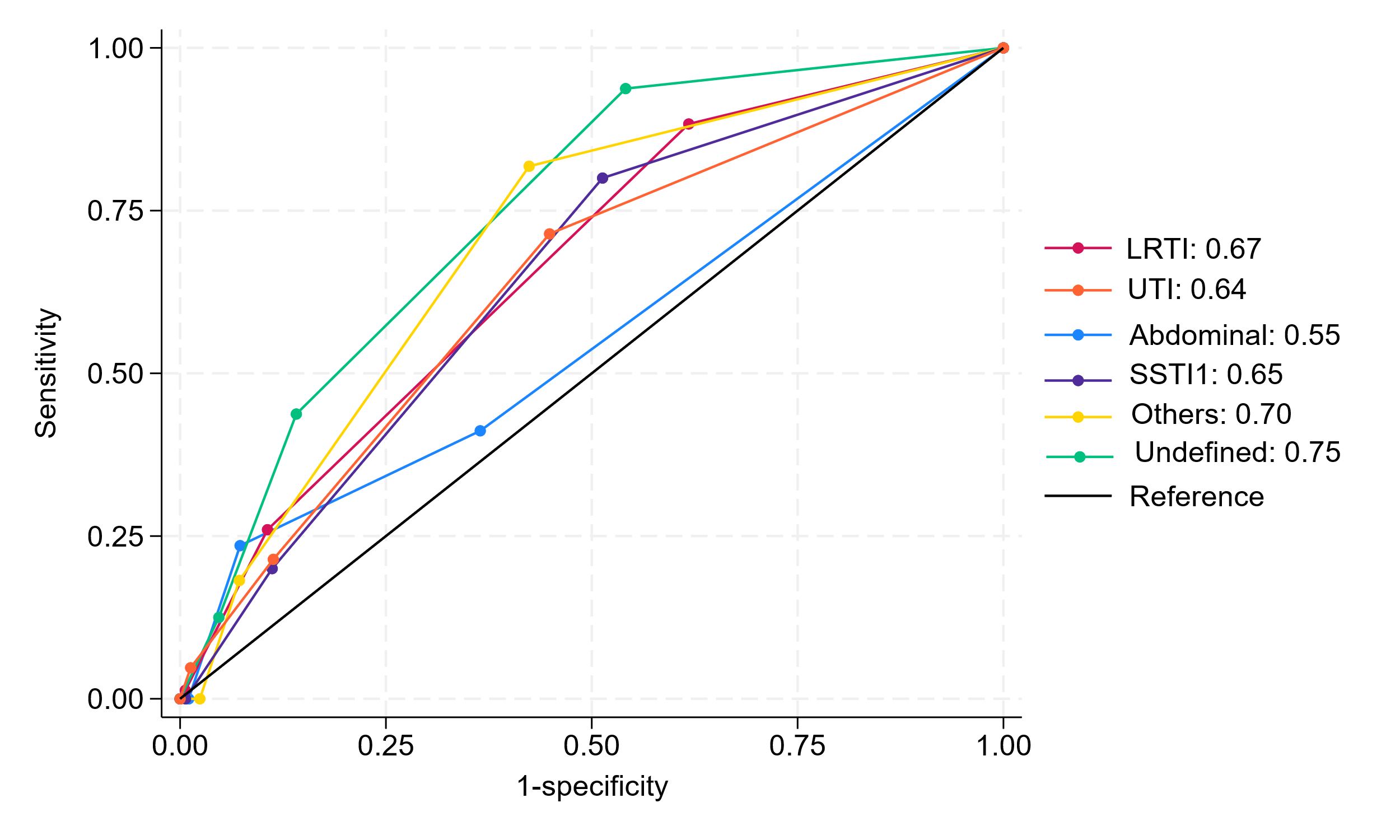


A)

B)

C)

D)

**Figure S18.** Imputed data. Receiver operating characteristic curve in the different subcohorts for EWS in the discrimination of 30-day mortality. A) MEWS B) NEWS C) NEWS-2 D) qSOFA.

**Appendix 1. Power calculation.**

The statistical power for detecting the expected difference in the area under the receiver operating characteristics curve (AUC-ROC) was determined using a fixed number of patients, based on the existing Acutelines cohort, with a type I error fixed at 0.05. The calculation followed the formula described by Hanley and McNeil (1, 2). With the lack of expected AUC values specific to different sources of infections, our targeted AUC values were 0.6 and 0.7, based on the findings reported by Madrazo et al. and Kolditz et al., respectively (3, 4). Given that we took all the available cases and considering the variation in sample size for the different categories in source of infection, a simulation was conducted with increasing sample sizes ranging from 100 to 500 and incrementing gradually by 50. Resulting in a power of: 0.36, 0.48, 0.61, 0.72, 0.81, 0.85, 0.89, 0.94, and 0.95, respectively.

**References**

1. Hanley JA, McNeil BJ. The meaning and use of the area under a receiver operating characteristic (ROC) curve. Radiology. 1982;143(1):29-36.

2. Hanley JA, McNeil BJ. A method of comparing the areas under receiver operating characteristic curves derived from the same cases. Radiology. 1983;148(3):839-43.

3. Kolditz M, Scherag A, Rohde G, Ewig S, Welte T, Pletz M, Group CS. Comparison of the qSOFA and CRB-65 for risk prediction in patients with community-acquired pneumonia. Intensive Care Med. 42. United States2016. p. 2108-10.

4. Madrazo M, Piles L, López-Cruz I, Alberola J, Eiros JM, Zaragoza R, Artero A. Comparison of quick Pitt to quick sofa and sofa scores for scoring of severity for patients with urinary tract infection. Intern Emerg Med. 2022;17(5):1321-6.

**Appendix 2. Pseudo-calibration analysis.**

For this pseudo-calibration analysis, we treated the EWS as binary, using the original score cutoffs: qSOFA ≥2, MEWS ≥5, and NEWS/NEWS-2 ≥7.

**In-hospital mortality prediction.**

**LRTI**

**MEWS** Table collapsed on quantiles of estimated probabilities

+--------------------------------------------------------+

| Group | Prob | Obs_1 | Exp_1 | Obs_0 | Exp_0 | Total |

|-------+--------+-------+-------+-------+-------+-------|

| 7 | 0.0562 | 24 | 24.0 | 403 | 403.0 | 427 |

| 10 | 0.2397 | 29 | 29.0 | 92 | 92.0 | 121 |

+--------------------------------------------------------+

Number of observations = 548

Number of groups = 2

Hosmer–Lemeshow chi2(0) = 0.00

Prob > chi2 = .

Warning: There are only 2 distinct quantiles because of ties.

**NEWS** Table collapsed on quantiles of estimated probabilities

+--------------------------------------------------------+

| Group | Prob | Obs_1 | Exp_1 | Obs_0 | Exp_0 | Total |

|-------+--------+-------+-------+-------+-------+-------|

| 6 | 0.0427 | 16 | 16.0 | 359 | 359.0 | 375 |

| 10 | 0.2139 | 37 | 37.0 | 136 | 136.0 | 173 |

+--------------------------------------------------------+

Number of observations = 548

Number of groups = 2

Hosmer–Lemeshow chi2(0) = 0.00

Prob > chi2 = .

Warning: There are only 2 distinct quantiles because of ties.

**NEWS-2** Table collapsed on quantiles of estimated probabilities

+--------------------------------------------------------+

| Group | Prob | Obs_1 | Exp_1 | Obs_0 | Exp_0 | Total |

|-------+--------+-------+-------+-------+-------+-------|

| 6 | 0.0434 | 16 | 16.0 | 353 | 353.0 | 369 |

| 10 | 0.2067 | 37 | 37.0 | 142 | 142.0 | 179 |

+--------------------------------------------------------+

Number of observations = 548

Number of groups = 2

Hosmer–Lemeshow chi2(0) = 0.00

Prob > chi2 = .

Warning: There are only 2 distinct quantiles because of ties.

**qSOFA**
 Table collapsed on quantiles of estimated probabilities

+--------------------------------------------------------+

| Group | Prob | Obs_1 | Exp_1 | Obs_0 | Exp_0 | Total |

|-------+--------+-------+-------+-------+-------+-------|

| 8 | 0.0782 | 38 | 38.0 | 448 | 448.0 | 486 |

| 10 | 0.2419 | 15 | 15.0 | 47 | 47.0 | 62 |

+--------------------------------------------------------+

Number of observations = 548

Number of groups = 2

Hosmer–Lemeshow chi2(0) = 0.00

Prob > chi2 = .

Warning: There are only 2 distinct quantiles because of ties.

**UTI**

**MEWS** Table collapsed on quantiles of estimated probabilities

+--------------------------------------------------------+

| Group | Prob | Obs_1 | Exp_1 | Obs_0 | Exp_0 | Total |

|-------+--------+-------+-------+-------+-------+-------|

| 8 | 0.0421 | 19 | 19.0 | 432 | 432.0 | 451 |

| 10 | 0.0847 | 5 | 5.0 | 54 | 54.0 | 59 |

+--------------------------------------------------------+

Number of observations = 510

Number of groups = 2

Hosmer–Lemeshow chi2(0) = 0.00

Prob > chi2 = .

Warning: There are only 2 distinct quantiles because of ties.

**NEWS** Table collapsed on quantiles of estimated probabilities

+--------------------------------------------------------+

| Group | Prob | Obs_1 | Exp_1 | Obs_0 | Exp_0 | Total |

|-------+--------+-------+-------+-------+-------+-------|

| 8 | 0.0409 | 18 | 18.0 | 422 | 422.0 | 440 |

| 10 | 0.0857 | 6 | 6.0 | 64 | 64.0 | 70 |

+--------------------------------------------------------+

Number of observations = 510

Number of groups = 2

Hosmer–Lemeshow chi2(0) = 0.00

Prob > chi2 = .

Warning: There are only 2 distinct quantiles because of ties.

**NEWS-2** Table collapsed on quantiles of estimated probabilities

+--------------------------------------------------------+

| Group | Prob | Obs_1 | Exp_1 | Obs_0 | Exp_0 | Total |

|-------+--------+-------+-------+-------+-------+-------|

| 8 | 0.0394 | 17 | 17.0 | 414 | 414.0 | 431 |

| 10 | 0.0886 | 7 | 7.0 | 72 | 72.0 | 79 |

+--------------------------------------------------------+

Number of observations = 510

Number of groups = 2

Hosmer–Lemeshow chi2(0) = 0.00

Prob > chi2 = .

Warning: There are only 2 distinct quantiles because of ties.

**qSOFA** Table collapsed on quantiles of estimated probabilities

+--------------------------------------------------------+

| Group | Prob | Obs_1 | Exp_1 | Obs_0 | Exp_0 | Total |

|-------+--------+-------+-------+-------+-------+-------|

| 8 | 0.0437 | 20 | 20.0 | 438 | 438.0 | 458 |

| 10 | 0.0769 | 4 | 4.0 | 48 | 48.0 | 52 |

+--------------------------------------------------------+

Number of observations = 510

Number of groups = 2

Hosmer–Lemeshow chi2(0) = 0.00

Prob > chi2 = .

Warning: There are only 2 distinct quantiles because of ties.

**Abdominal**

**MEWS** Table collapsed on quantiles of estimated probabilities

+--------------------------------------------------------+

| Group | Prob | Obs_1 | Exp_1 | Obs_0 | Exp_0 | Total |

|-------+--------+-------+-------+-------+-------+-------|

| 8 | 0.0276 | 5 | 5.0 | 176 | 176.0 | 181 |

| 10 | 0.1786 | 5 | 5.0 | 23 | 23.0 | 28 |

+--------------------------------------------------------+

Number of observations = 209

Number of groups = 2

Hosmer–Lemeshow chi2(0) = 0.00

Prob > chi2 = .

Warning: There are only 2 distinct quantiles because of ties.

**NEWS** Table collapsed on quantiles of estimated probabilities

+--------------------------------------------------------+

| Group | Prob | Obs_1 | Exp_1 | Obs_0 | Exp_0 | Total |

|-------+--------+-------+-------+-------+-------+-------|

| 8 | 0.0278 | 5 | 5.0 | 175 | 175.0 | 180 |

| 10 | 0.1724 | 5 | 5.0 | 24 | 24.0 | 29 |

+--------------------------------------------------------+

Number of observations = 209

Number of groups = 2

Hosmer–Lemeshow chi2(0) = 0.00

Prob > chi2 = .

Warning: There are only 2 distinct quantiles because of ties.

**NEWS-2** Table collapsed on quantiles of estimated probabilities

+--------------------------------------------------------+

| Group | Prob | Obs_1 | Exp_1 | Obs_0 | Exp_0 | Total |

|-------+--------+-------+-------+-------+-------+-------|

| 8 | 0.0278 | 5 | 5.0 | 175 | 175.0 | 180 |

| 10 | 0.1724 | 5 | 5.0 | 24 | 24.0 | 29 |

+--------------------------------------------------------+

Number of observations = 209

Number of groups = 2

Hosmer–Lemeshow chi2(0) = 0.00

Prob > chi2 = .

Warning: There are only 2 distinct quantiles because of ties.

**qSOFA**

Table collapsed on quantiles of estimated probabilities

+--------------------------------------------------------+

| Group | Prob | Obs_1 | Exp_1 | Obs_0 | Exp_0 | Total |

|-------+--------+-------+-------+-------+-------+-------|

| 9 | 0.0314 | 6 | 6.0 | 185 | 185.0 | 191 |

| 10 | 0.2222 | 4 | 4.0 | 14 | 14.0 | 18 |

+--------------------------------------------------------+

Number of observations = 209

Number of groups = 2

Hosmer–Lemeshow chi2(0) = 0.00

Prob > chi2 = .

Warning: There are only 2 distinct quantiles because of ties.

**SSTI**

**MEWS**

not enough degrees of freedom to perform the test

**NEWS**

not enough degrees of freedom to perform the test

**NEWS-2**

Table collapsed on quantiles of estimated probabilities

+--------------------------------------------------------+

| Group | Prob | Obs_1 | Exp_1 | Obs_0 | Exp_0 | Total |

|-------+--------+-------+-------+-------+-------+-------|

| 8 | 0.0152 | 2 | 2.0 | 130 | 130.0 | 132 |

| 10 | 0.0400 | 1 | 1.0 | 24 | 24.0 | 25 |

+--------------------------------------------------------+

Number of observations = 157

Number of groups = 2

Hosmer–Lemeshow chi2(0) = 0.00

Prob > chi2 = .

Warning: There are only 2 distinct quantiles because of ties.

**qSOFA**

not enough degrees of freedom to perform the test

**Others**

**MEWS**

Table collapsed on quantiles of estimated probabilities

+--------------------------------------------------------+

| Group | Prob | Obs_1 | Exp_1 | Obs_0 | Exp_0 | Total |

|-------+--------+-------+-------+-------+-------+-------|

| 8 | 0.0504 | 6 | 6.0 | 113 | 113.0 | 119 |

| 10 | 0.0588 | 1 | 1.0 | 16 | 16.0 | 17 |

+--------------------------------------------------------+

Number of observations = 136

Number of groups = 2

Hosmer–Lemeshow chi2(0) = 0.00

Prob > chi2 = .

Warning: There are only 2 distinct quantiles because of ties.

**NEWS**

Table collapsed on quantiles of estimated probabilities

+--------------------------------------------------------+

| Group | Prob | Obs_1 | Exp_1 | Obs_0 | Exp_0 | Total |

|-------+--------+-------+-------+-------+-------+-------|

| 8 | 0.0420 | 5 | 5.0 | 114 | 114.0 | 119 |

| 10 | 0.1176 | 2 | 2.0 | 15 | 15.0 | 17 |

+--------------------------------------------------------+

Number of observations = 136

Number of groups = 2

Hosmer–Lemeshow chi2(0) = 0.00

Prob > chi2 = .

Warning: There are only 2 distinct quantiles because of ties.

**NEWS-2**

Table collapsed on quantiles of estimated probabilities

+--------------------------------------------------------+

| Group | Prob | Obs_1 | Exp_1 | Obs_0 | Exp_0 | Total |

|-------+--------+-------+-------+-------+-------+-------|

| 8 | 0.0420 | 5 | 5.0 | 114 | 114.0 | 119 |

| 10 | 0.1176 | 2 | 2.0 | 15 | 15.0 | 17 |

+--------------------------------------------------------+

Number of observations = 136

Number of groups = 2

Hosmer–Lemeshow chi2(0) = 0.00

Prob > chi2 = .

Warning: There are only 2 distinct quantiles because of ties.

**qSOFA**

Table collapsed on quantiles of estimated probabilities

+--------------------------------------------------------+

| Group | Prob | Obs_1 | Exp_1 | Obs_0 | Exp_0 | Total |

|-------+--------+-------+-------+-------+-------+-------|

| 9 | 0.0472 | 6 | 6.0 | 121 | 121.0 | 127 |

| 10 | 0.1111 | 1 | 1.0 | 8 | 8.0 | 9 |

+--------------------------------------------------------+

Number of observations = 136

Number of groups = 2

Hosmer–Lemeshow chi2(0) = 0.00

Prob > chi2 = .

Warning: There are only 2 distinct quantiles because of ties.

**Undefined**

**MEWS**

Table collapsed on quantiles of estimated probabilities

+--------------------------------------------------------+

| Group | Prob | Obs_1 | Exp_1 | Obs_0 | Exp_0 | Total |

|-------+--------+-------+-------+-------+-------+-------|

| 7 | 0.1184 | 9 | 9.0 | 67 | 67.0 | 76 |

| 10 | 0.2400 | 6 | 6.0 | 19 | 19.0 | 25 |

+--------------------------------------------------------+

Number of observations = 101

Number of groups = 2

Hosmer–Lemeshow chi2(0) = 0.00

Prob > chi2 = .

Warning: There are only 2 distinct quantiles because of ties.

**NEWS** Table collapsed on quantiles of estimated probabilities

+--------------------------------------------------------+

| Group | Prob | Obs_1 | Exp_1 | Obs_0 | Exp_0 | Total |

|-------+--------+-------+-------+-------+-------+-------|

| 7 | 0.1053 | 8 | 8.0 | 68 | 68.0 | 76 |

| 10 | 0.2800 | 7 | 7.0 | 18 | 18.0 | 25 |

+--------------------------------------------------------+

Number of observations = 101

Number of groups = 2

Hosmer–Lemeshow chi2(0) = 0.00

Prob > chi2 = .

Warning: There are only 2 distinct quantiles because of ties.

**NEWS-2**

Table collapsed on quantiles of estimated probabilities

+--------------------------------------------------------+

| Group | Prob | Obs_1 | Exp_1 | Obs_0 | Exp_0 | Total |

|-------+--------+-------+-------+-------+-------+-------|

| 6 | 0.1143 | 8 | 8.0 | 62 | 62.0 | 70 |

| 10 | 0.2258 | 7 | 7.0 | 24 | 24.0 | 31 |

+--------------------------------------------------------+

Number of observations = 101

Number of groups = 2

Hosmer–Lemeshow chi2(0) = 0.00

Prob > chi2 = .

Warning: There are only 2 distinct quantiles because of ties.

**qSOFA**

Table collapsed on quantiles of estimated probabilities

+--------------------------------------------------------+

| Group | Prob | Obs_1 | Exp_1 | Obs_0 | Exp_0 | Total |

|-------+--------+-------+-------+-------+-------+-------|

| 8 | 0.1176 | 10 | 10.0 | 75 | 75.0 | 85 |

| 10 | 0.3125 | 5 | 5.0 | 11 | 11.0 | 16 |

+--------------------------------------------------------+

Number of observations = 101

Number of groups = 2

Hosmer–Lemeshow chi2(0) = 0.00

Prob > chi2 = .

Warning: There are only 2 distinct quantiles because of ties.

**ICU admission prediction.**

**LRTI**

**MEWS** Table collapsed on quantiles of estimated probabilities

+--------------------------------------------------------+

| Group | Prob | Obs_1 | Exp_1 | Obs_0 | Exp_0 | Total |

|-------+--------+-------+-------+-------+-------+-------|

| 7 | 0.0890 | 38 | 38.0 | 389 | 389.0 | 427 |

| 10 | 0.1901 | 23 | 23.0 | 98 | 98.0 | 121 |

+--------------------------------------------------------+

Number of observations = 548

Number of groups = 2

Hosmer–Lemeshow chi2(0) = 0.00

Prob > chi2 = .

Warning: There are only 2 distinct quantiles because of ties.

**NEWS** Table collapsed on quantiles of estimated probabilities

+--------------------------------------------------------+

| Group | Prob | Obs_1 | Exp_1 | Obs_0 | Exp_0 | Total |

|-------+--------+-------+-------+-------+-------+-------|

| 6 | 0.0693 | 26 | 26.0 | 349 | 349.0 | 375 |

| 10 | 0.2023 | 35 | 35.0 | 138 | 138.0 | 173 |

+--------------------------------------------------------+

Number of observations = 548

Number of groups = 2

Hosmer–Lemeshow chi2(0) = 0.00

Prob > chi2 = .

Warning: There are only 2 distinct quantiles because of ties.

**NEWS-2** Table collapsed on quantiles of estimated probabilities

+--------------------------------------------------------+

| Group | Prob | Obs_1 | Exp_1 | Obs_0 | Exp_0 | Total |

|-------+--------+-------+-------+-------+-------+-------|

| 6 | 0.0678 | 25 | 25.0 | 344 | 344.0 | 369 |

| 10 | 0.2011 | 36 | 36.0 | 143 | 143.0 | 179 |

+--------------------------------------------------------+

Number of observations = 548

Number of groups = 2

Hosmer–Lemeshow chi2(0) = 0.00

Prob > chi2 = .

Warning: There are only 2 distinct quantiles because of ties.

**qSOFA** Table collapsed on quantiles of estimated probabilities

+--------------------------------------------------------+

| Group | Prob | Obs_1 | Exp_1 | Obs_0 | Exp_0 | Total |

|-------+--------+-------+-------+-------+-------+-------|

| 8 | 0.0947 | 46 | 46.0 | 440 | 440.0 | 486 |

| 10 | 0.2419 | 15 | 15.0 | 47 | 47.0 | 62 |

+--------------------------------------------------------+

Number of observations = 548

Number of groups = 2

Hosmer–Lemeshow chi2(0) = 0.00

Prob > chi2 = .

Warning: There are only 2 distinct quantiles because of ties.

**UTI**

**MEWS**

Table collapsed on quantiles of estimated probabilities

+--------------------------------------------------------+

| Group | Prob | Obs_1 | Exp_1 | Obs_0 | Exp_0 | Total |

|-------+--------+-------+-------+-------+-------+-------|

| 8 | 0.0155 | 7 | 7.0 | 444 | 444.0 | 451 |

| 10 | 0.1186 | 7 | 7.0 | 52 | 52.0 | 59 |

+--------------------------------------------------------+

Number of observations = 510

Number of groups = 2

Hosmer–Lemeshow chi2(0) = 0.00

Prob > chi2 = .

Warning: There are only 2 distinct quantiles because of ties.

**NEWS** Table collapsed on quantiles of estimated probabilities

+--------------------------------------------------------+

| Group | Prob | Obs_1 | Exp_1 | Obs_0 | Exp_0 | Total |

|-------+--------+-------+-------+-------+-------+-------|

| 8 | 0.0136 | 6 | 6.0 | 434 | 434.0 | 440 |

| 10 | 0.1143 | 8 | 8.0 | 62 | 62.0 | 70 |

+--------------------------------------------------------+

Number of observations = 510

Number of groups = 2

Hosmer–Lemeshow chi2(0) = 0.00

Prob > chi2 = .

Warning: There are only 2 distinct quantiles because of ties.

**NEWS-2**

Table collapsed on quantiles of estimated probabilities

+--------------------------------------------------------+

| Group | Prob | Obs_1 | Exp_1 | Obs_0 | Exp_0 | Total |

|-------+--------+-------+-------+-------+-------+-------|

| 8 | 0.0139 | 6 | 6.0 | 425 | 425.0 | 431 |

| 10 | 0.1013 | 8 | 8.0 | 71 | 71.0 | 79 |

+--------------------------------------------------------+

Number of observations = 510

Number of groups = 2

Hosmer–Lemeshow chi2(0) = 0.00

Prob > chi2 = .

Warning: There are only 2 distinct quantiles because of ties.

**qSOFA**

Table collapsed on quantiles of estimated probabilities

+--------------------------------------------------------+

| Group | Prob | Obs_1 | Exp_1 | Obs_0 | Exp_0 | Total |

|-------+--------+-------+-------+-------+-------+-------|

| 8 | 0.0153 | 7 | 7.0 | 451 | 451.0 | 458 |

| 10 | 0.1346 | 7 | 7.0 | 45 | 45.0 | 52 |

+--------------------------------------------------------+

Number of observations = 510

Number of groups = 2

Hosmer–Lemeshow chi2(0) = 0.00

Prob > chi2 = .

Warning: There are only 2 distinct quantiles because of ties.

**Abdominal**

**MEWS**
 Table collapsed on quantiles of estimated probabilities

+--------------------------------------------------------+

| Group | Prob | Obs_1 | Exp_1 | Obs_0 | Exp_0 | Total |

|-------+--------+-------+-------+-------+-------+-------|

| 8 | 0.0387 | 7 | 7.0 | 174 | 174.0 | 181 |

| 10 | 0.1786 | 5 | 5.0 | 23 | 23.0 | 28 |

+--------------------------------------------------------+

Number of observations = 209

Number of groups = 2

Hosmer–Lemeshow chi2(0) = 0.00

Prob > chi2 = .

Warning: There are only 2 distinct quantiles because of ties.

**NEWS** Table collapsed on quantiles of estimated probabilities

+--------------------------------------------------------+

| Group | Prob | Obs_1 | Exp_1 | Obs_0 | Exp_0 | Total |

|-------+--------+-------+-------+-------+-------+-------|

| 8 | 0.0389 | 7 | 7.0 | 173 | 173.0 | 180 |

| 10 | 0.1724 | 5 | 5.0 | 24 | 24.0 | 29 |

+--------------------------------------------------------+

Number of observations = 209

Number of groups = 2

Hosmer–Lemeshow chi2(0) = 0.00

Prob > chi2 = .

Warning: There are only 2 distinct quantiles because of ties.

**NEWS-2** Table collapsed on quantiles of estimated probabilities

+--------------------------------------------------------+

| Group | Prob | Obs_1 | Exp_1 | Obs_0 | Exp_0 | Total |

|-------+--------+-------+-------+-------+-------+-------|

| 8 | 0.0389 | 7 | 7.0 | 173 | 173.0 | 180 |

| 10 | 0.1724 | 5 | 5.0 | 24 | 24.0 | 29 |

+--------------------------------------------------------+

Number of observations = 209

Number of groups = 2

Hosmer–Lemeshow chi2(0) = 0.00

Prob > chi2 = .

Warning: There are only 2 distinct quantiles because of ties.

**qSOFA** Table collapsed on quantiles of estimated probabilities

+--------------------------------------------------------+

| Group | Prob | Obs_1 | Exp_1 | Obs_0 | Exp_0 | Total |

|-------+--------+-------+-------+-------+-------+-------|

| 9 | 0.0314 | 6 | 6.0 | 185 | 185.0 | 191 |

| 10 | 0.3333 | 6 | 6.0 | 12 | 12.0 | 18 |

+--------------------------------------------------------+

Number of observations = 209

Number of groups = 2

Hosmer–Lemeshow chi2(0) = 0.00

Prob > chi2 = .

Warning: There are only 2 distinct quantiles because of ties.

**SSTI**

**MEWS** Table collapsed on quantiles of estimated probabilities

+--------------------------------------------------------+

| Group | Prob | Obs_1 | Exp_1 | Obs_0 | Exp_0 | Total |

|-------+--------+-------+-------+-------+-------+-------|

| 8 | 0.0382 | 5 | 5.0 | 126 | 126.0 | 131 |

| 10 | 0.2308 | 6 | 6.0 | 20 | 20.0 | 26 |

+--------------------------------------------------------+

Number of observations = 157

Number of groups = 2

Hosmer–Lemeshow chi2(0) = 0.00

Prob > chi2 = .

Warning: There are only 2 distinct quantiles because of ties.

**NEWS** Table collapsed on quantiles of estimated probabilities

+--------------------------------------------------------+

| Group | Prob | Obs_1 | Exp_1 | Obs_0 | Exp_0 | Total |

|-------+--------+-------+-------+-------+-------+-------|

| 8 | 0.0303 | 4 | 4.0 | 128 | 128.0 | 132 |

| 10 | 0.2800 | 7 | 7.0 | 18 | 18.0 | 25 |

+--------------------------------------------------------+

Number of observations = 157

Number of groups = 2

Hosmer–Lemeshow chi2(0) = 0.00

Prob > chi2 = .

Warning: There are only 2 distinct quantiles because of ties.

**NEWS-2**

Table collapsed on quantiles of estimated probabilities

+--------------------------------------------------------+

| Group | Prob | Obs_1 | Exp_1 | Obs_0 | Exp_0 | Total |

|-------+--------+-------+-------+-------+-------+-------|

| 8 | 0.0310 | 4 | 4.0 | 125 | 125.0 | 129 |

| 10 | 0.2500 | 7 | 7.0 | 21 | 21.0 | 28 |

+--------------------------------------------------------+

Number of observations = 157

Number of groups = 2

Hosmer–Lemeshow chi2(0) = 0.00

Prob > chi2 = .

Warning: There are only 2 distinct quantiles because of ties.

**qSOFA**

Table collapsed on quantiles of estimated probabilities

+--------------------------------------------------------+

| Group | Prob | Obs_1 | Exp_1 | Obs_0 | Exp_0 | Total |

|-------+--------+-------+-------+-------+-------+-------|

| 9 | 0.0420 | 6 | 6.0 | 137 | 137.0 | 143 |

| 10 | 0.3571 | 5 | 5.0 | 9 | 9.0 | 14 |

+--------------------------------------------------------+

Number of observations = 157

Number of groups = 2

Hosmer–Lemeshow chi2(0) = 0.00

Prob > chi2 = .

Warning: There are only 2 distinct quantiles because of ties.

**Others**

**MEWS** Table collapsed on quantiles of estimated probabilities

+--------------------------------------------------------+

| Group | Prob | Obs_1 | Exp_1 | Obs_0 | Exp_0 | Total |

|-------+--------+-------+-------+-------+-------+-------|

| 8 | 0.0336 | 4 | 4.0 | 115 | 115.0 | 119 |

| 10 | 0.2353 | 4 | 4.0 | 13 | 13.0 | 17 |

+--------------------------------------------------------+

Number of observations = 136

Number of groups = 2

Hosmer–Lemeshow chi2(0) = 0.00

Prob > chi2 = .

Warning: There are only 2 distinct quantiles because of ties.

**NEWS** Table collapsed on quantiles of estimated probabilities

+--------------------------------------------------------+

| Group | Prob | Obs_1 | Exp_1 | Obs_0 | Exp_0 | Total |

|-------+--------+-------+-------+-------+-------+-------|

| 8 | 0.0252 | 3 | 3.0 | 116 | 116.0 | 119 |

| 10 | 0.2941 | 5 | 5.0 | 12 | 12.0 | 17 |

+--------------------------------------------------------+

Number of observations = 136

Number of groups = 2

Hosmer–Lemeshow chi2(0) = 0.00

Prob > chi2 = .

Warning: There are only 2 distinct quantiles because of ties.

**NEWS-2** Table collapsed on quantiles of estimated probabilities

+--------------------------------------------------------+

| Group | Prob | Obs_1 | Exp_1 | Obs_0 | Exp_0 | Total |

|-------+--------+-------+-------+-------+-------+-------|

| 8 | 0.0252 | 3 | 3.0 | 116 | 116.0 | 119 |

| 10 | 0.2941 | 5 | 5.0 | 12 | 12.0 | 17 |

+--------------------------------------------------------+

Number of observations = 136

Number of groups = 2

Hosmer–Lemeshow chi2(0) = 0.00

Prob > chi2 = .

Warning: There are only 2 distinct quantiles because of ties.

**qSOFA**

Table collapsed on quantiles of estimated probabilities

+--------------------------------------------------------+

| Group | Prob | Obs_1 | Exp_1 | Obs_0 | Exp_0 | Total |

|-------+--------+-------+-------+-------+-------+-------|

| 9 | 0.0551 | 7 | 7.0 | 120 | 120.0 | 127 |

| 10 | 0.1111 | 1 | 1.0 | 8 | 8.0 | 9 |

+--------------------------------------------------------+

Number of observations = 136

Number of groups = 2

Hosmer–Lemeshow chi2(0) = 0.00

Prob > chi2 = .

Warning: There are only 2 distinct quantiles because of ties.

**Undefined**

**MEWS** Table collapsed on quantiles of estimated probabilities

+--------------------------------------------------------+

| Group | Prob | Obs_1 | Exp_1 | Obs_0 | Exp_0 | Total |

|-------+--------+-------+-------+-------+-------+-------|

| 7 | 0.0789 | 6 | 6.0 | 70 | 70.0 | 76 |

| 10 | 0.2400 | 6 | 6.0 | 19 | 19.0 | 25 |

+--------------------------------------------------------+

Number of observations = 101

Number of groups = 2

Hosmer–Lemeshow chi2(0) = 0.00

Prob > chi2 = .

Warning: There are only 2 distinct quantiles because of ties.

**NEWS**

Table collapsed on quantiles of estimated probabilities

+--------------------------------------------------------+

| Group | Prob | Obs_1 | Exp_1 | Obs_0 | Exp_0 | Total |

|-------+--------+-------+-------+-------+-------+-------|

| 7 | 0.0789 | 6 | 6.0 | 70 | 70.0 | 76 |

| 10 | 0.2400 | 6 | 6.0 | 19 | 19.0 | 25 |

+--------------------------------------------------------+

Number of observations = 101

Number of groups = 2

Hosmer–Lemeshow chi2(0) = 0.00

Prob > chi2 = .

Warning: There are only 2 distinct quantiles because of ties.

**NEWS-2**

Table collapsed on quantiles of estimated probabilities

+--------------------------------------------------------+

| Group | Prob | Obs_1 | Exp_1 | Obs_0 | Exp_0 | Total |

|-------+--------+-------+-------+-------+-------+-------|

| 6 | 0.0857 | 6 | 6.0 | 64 | 64.0 | 70 |

| 10 | 0.1935 | 6 | 6.0 | 25 | 25.0 | 31 |

+--------------------------------------------------------+

Number of observations = 101

Number of groups = 2

Hosmer–Lemeshow chi2(0) = 0.00

Prob > chi2 = .

Warning: There are only 2 distinct quantiles because of ties.

**qSOFA**

Table collapsed on quantiles of estimated probabilities

+--------------------------------------------------------+

| Group | Prob | Obs_1 | Exp_1 | Obs_0 | Exp_0 | Total |

|-------+--------+-------+-------+-------+-------+-------|

| 8 | 0.1176 | 10 | 10.0 | 75 | 75.0 | 85 |

| 10 | 0.1250 | 2 | 2.0 | 14 | 14.0 | 16 |

+--------------------------------------------------------+

Number of observations = 101

Number of groups = 2

Hosmer–Lemeshow chi2(0) = 0.00

Prob > chi2 = .

Warning: There are only 2 distinct quantiles because of ties.

**30 day mortality prediction.**

**LRTI**

**MEWS** Table collapsed on quantiles of estimated probabilities

+--------------------------------------------------------+

| Group | Prob | Obs_1 | Exp_1 | Obs_0 | Exp_0 | Total |

|-------+--------+-------+-------+-------+-------+-------|

| 7 | 0.0984 | 42 | 42.0 | 385 | 385.0 | 427 |

| 10 | 0.2893 | 35 | 35.0 | 86 | 86.0 | 121 |

+--------------------------------------------------------+

Number of observations = 548

Number of groups = 2

Hosmer–Lemeshow chi2(0) = 0.00

Prob > chi2 = .

Warning: There are only 2 distinct quantiles because of ties.

**NEWS**

Table collapsed on quantiles of estimated probabilities

+--------------------------------------------------------+

| Group | Prob | Obs_1 | Exp_1 | Obs_0 | Exp_0 | Total |

|-------+--------+-------+-------+-------+-------+-------|

| 6 | 0.0853 | 32 | 32.0 | 343 | 343.0 | 375 |

| 10 | 0.2601 | 45 | 45.0 | 128 | 128.0 | 173 |

+--------------------------------------------------------+

Number of observations = 548

Number of groups = 2

Hosmer–Lemeshow chi2(0) = 0.00

Prob > chi2 = .

Warning: There are only 2 distinct quantiles because of ties.

**NEWS-2**

Table collapsed on quantiles of estimated probabilities

+--------------------------------------------------------+

| Group | Prob | Obs_1 | Exp_1 | Obs_0 | Exp_0 | Total |

|-------+--------+-------+-------+-------+-------+-------|

| 6 | 0.0840 | 31 | 31.0 | 338 | 338.0 | 369 |

| 10 | 0.2570 | 46 | 46.0 | 133 | 133.0 | 179 |

+--------------------------------------------------------+

Number of observations = 548

Number of groups = 2

Hosmer–Lemeshow chi2(0) = 0.00

Prob > chi2 = .

Warning: There are only 2 distinct quantiles because of ties.

**qSOFA** Table collapsed on quantiles of estimated probabilities

+--------------------------------------------------------+

| Group | Prob | Obs_1 | Exp_1 | Obs_0 | Exp_0 | Total |

|-------+--------+-------+-------+-------+-------+-------|

| 8 | 0.1235 | 60 | 60.0 | 426 | 426.0 | 486 |

| 10 | 0.2742 | 17 | 17.0 | 45 | 45.0 | 62 |

+--------------------------------------------------------+

Number of observations = 548

Number of groups = 2

Hosmer–Lemeshow chi2(0) = 0.00

Prob > chi2 = .

Warning: There are only 2 distinct quantiles because of ties.

**UTI**

**MEWS** Table collapsed on quantiles of estimated probabilities

+--------------------------------------------------------+

| Group | Prob | Obs_1 | Exp_1 | Obs_0 | Exp_0 | Total |

|-------+--------+-------+-------+-------+-------+-------|

| 8 | 0.0754 | 34 | 34.0 | 417 | 417.0 | 451 |

| 10 | 0.1356 | 8 | 8.0 | 51 | 51.0 | 59 |

+--------------------------------------------------------+

Number of observations = 510

Number of groups = 2

Hosmer–Lemeshow chi2(0) = 0.00

Prob > chi2 = .

Warning: There are only 2 distinct quantiles because of ties.

**NEWS** Table collapsed on quantiles of estimated probabilities

+--------------------------------------------------------+

| Group | Prob | Obs_1 | Exp_1 | Obs_0 | Exp_0 | Total |

|-------+--------+-------+-------+-------+-------+-------|

| 8 | 0.0727 | 32 | 32.0 | 408 | 408.0 | 440 |

| 10 | 0.1429 | 10 | 10.0 | 60 | 60.0 | 70 |

+--------------------------------------------------------+

Number of observations = 510

Number of groups = 2

Hosmer–Lemeshow chi2(0) = 0.00

Prob > chi2 = .

Warning: There are only 2 distinct quantiles because of ties.

**NEWS-2** Table collapsed on quantiles of estimated probabilities

+--------------------------------------------------------+

| Group | Prob | Obs_1 | Exp_1 | Obs_0 | Exp_0 | Total |

|-------+--------+-------+-------+-------+-------+-------|

| 8 | 0.0696 | 30 | 30.0 | 401 | 401.0 | 431 |

| 10 | 0.1519 | 12 | 12.0 | 67 | 67.0 | 79 |

+--------------------------------------------------------+

Number of observations = 510

Number of groups = 2

Hosmer–Lemeshow chi2(0) = 0.00

Prob > chi2 = .

Warning: There are only 2 distinct quantiles because of ties.

**qSOFA** Table collapsed on quantiles of estimated probabilities

+--------------------------------------------------------+

| Group | Prob | Obs_1 | Exp_1 | Obs_0 | Exp_0 | Total |

|-------+--------+-------+-------+-------+-------+-------|

| 8 | 0.0764 | 35 | 35.0 | 423 | 423.0 | 458 |

| 10 | 0.1346 | 7 | 7.0 | 45 | 45.0 | 52 |

+--------------------------------------------------------+

Number of observations = 510

Number of groups = 2

Hosmer–Lemeshow chi2(0) = 0.00

Prob > chi2 = .

Warning: There are only 2 distinct quantiles because of ties.

**Abdominal**

**MEWS** Table collapsed on quantiles of estimated probabilities

+--------------------------------------------------------+

| Group | Prob | Obs_1 | Exp_1 | Obs_0 | Exp_0 | Total |

|-------+--------+-------+-------+-------+-------+-------|

| 8 | 0.0552 | 10 | 10.0 | 171 | 171.0 | 181 |

| 10 | 0.2500 | 7 | 7.0 | 21 | 21.0 | 28 |

+--------------------------------------------------------+

Number of observations = 209

Number of groups = 2

Hosmer–Lemeshow chi2(0) = 0.00

Prob > chi2 = .

Warning: There are only 2 distinct quantiles because of ties.

**NEWS** Table collapsed on quantiles of estimated probabilities

+--------------------------------------------------------+

| Group | Prob | Obs_1 | Exp_1 | Obs_0 | Exp_0 | Total |

|-------+--------+-------+-------+-------+-------+-------|

| 8 | 0.0556 | 10 | 10.0 | 170 | 170.0 | 180 |

| 10 | 0.2414 | 7 | 7.0 | 22 | 22.0 | 29 |

+--------------------------------------------------------+

Number of observations = 209

Number of groups = 2

Hosmer–Lemeshow chi2(0) = 0.00

Prob > chi2 = .

Warning: There are only 2 distinct quantiles because of ties.

**NEWS-2** Table collapsed on quantiles of estimated probabilities

+--------------------------------------------------------+

| Group | Prob | Obs_1 | Exp_1 | Obs_0 | Exp_0 | Total |

|-------+--------+-------+-------+-------+-------+-------|

| 8 | 0.0556 | 10 | 10.0 | 170 | 170.0 | 180 |

| 10 | 0.2414 | 7 | 7.0 | 22 | 22.0 | 29 |

+--------------------------------------------------------+

Number of observations = 209

Number of groups = 2

Hosmer–Lemeshow chi2(0) = 0.00

Prob > chi2 = .

Warning: There are only 2 distinct quantiles because of ties.

**qSOFA** Table collapsed on quantiles of estimated probabilities

+--------------------------------------------------------+

| Group | Prob | Obs_1 | Exp_1 | Obs_0 | Exp_0 | Total |

|-------+--------+-------+-------+-------+-------+-------|

| 9 | 0.0681 | 13 | 13.0 | 178 | 178.0 | 191 |

| 10 | 0.2222 | 4 | 4.0 | 14 | 14.0 | 18 |

+--------------------------------------------------------+

Number of observations = 209

Number of groups = 2

Hosmer–Lemeshow chi2(0) = 0.00

Prob > chi2 = .

Warning: There are only 2 distinct quantiles because of ties.

**SSTI**

**MEWS**

not enough degrees of freedom to perform the test

**NEWS** Table collapsed on quantiles of estimated probabilities

+--------------------------------------------------------+

| Group | Prob | Obs_1 | Exp_1 | Obs_0 | Exp_0 | Total |

|-------+--------+-------+-------+-------+-------+-------|

| 8 | 0.0303 | 4 | 4.0 | 128 | 128.0 | 132 |

| 10 | 0.0400 | 1 | 1.0 | 24 | 24.0 | 25 |

+--------------------------------------------------------+

Number of observations = 157

Number of groups = 2

Hosmer–Lemeshow chi2(0) = 0.00

Prob > chi2 = .

Warning: There are only 2 distinct quantiles because of ties.

**NEWS-2** Table collapsed on quantiles of estimated probabilities

+--------------------------------------------------------+

| Group | Prob | Obs_1 | Exp_1 | Obs_0 | Exp_0 | Total |

|-------+--------+-------+-------+-------+-------+-------|

| 8 | 0.0310 | 4 | 4.0 | 125 | 125.0 | 129 |

| 10 | 0.0357 | 1 | 1.0 | 27 | 27.0 | 28 |

+--------------------------------------------------------+

Number of observations = 157

Number of groups = 2

Hosmer–Lemeshow chi2(0) = 0.00

Prob > chi2 = .

Warning: There are only 2 distinct quantiles because of ties.

**qSOFA** Table collapsed on quantiles of estimated probabilities

+--------------------------------------------------------+

| Group | Prob | Obs_1 | Exp_1 | Obs_0 | Exp_0 | Total |

|-------+--------+-------+-------+-------+-------+-------|

| 9 | 0.0280 | 4 | 4.0 | 139 | 139.0 | 143 |

| 10 | 0.0714 | 1 | 1.0 | 13 | 13.0 | 14 |

+--------------------------------------------------------+

Number of observations = 157

Number of groups = 2

Hosmer–Lemeshow chi2(0) = 0.00

Prob > chi2 = .

Warning: There are only 2 distinct quantiles because of ties.

**Others**

**MEWS** Table collapsed on quantiles of estimated probabilities

+--------------------------------------------------------+

| Group | Prob | Obs_1 | Exp_1 | Obs_0 | Exp_0 | Total |

|-------+--------+-------+-------+-------+-------+-------|

| 1 | 0.0588 | 1 | 1.0 | 16 | 16.0 | 17 |

| 10 | 0.0840 | 10 | 10.0 | 109 | 109.0 | 119 |

+--------------------------------------------------------+

Number of observations = 136

Number of groups = 2

Hosmer–Lemeshow chi2(0) = 0.00

Prob > chi2 = .

Warning: There are only 2 distinct quantiles because of ties.

**NEWS** Table collapsed on quantiles of estimated probabilities

+--------------------------------------------------------+

| Group | Prob | Obs_1 | Exp_1 | Obs_0 | Exp_0 | Total |

|-------+--------+-------+-------+-------+-------+-------|

| 8 | 0.0756 | 9 | 9.0 | 110 | 110.0 | 119 |

| 10 | 0.1176 | 2 | 2.0 | 15 | 15.0 | 17 |

+--------------------------------------------------------+

Number of observations = 136

Number of groups = 2

Hosmer–Lemeshow chi2(0) = 0.00

Prob > chi2 = .

Warning: There are only 2 distinct quantiles because of ties.

**NEWS-2** Table collapsed on quantiles of estimated probabilities

+--------------------------------------------------------+

| Group | Prob | Obs_1 | Exp_1 | Obs_0 | Exp_0 | Total |

|-------+--------+-------+-------+-------+-------+-------|

| 8 | 0.0756 | 9 | 9.0 | 110 | 110.0 | 119 |

| 10 | 0.1176 | 2 | 2.0 | 15 | 15.0 | 17 |

+--------------------------------------------------------+

Number of observations = 136

Number of groups = 2

Hosmer–Lemeshow chi2(0) = 0.00

Prob > chi2 = .

Warning: There are only 2 distinct quantiles because of ties.

**qSOFA** Table collapsed on quantiles of estimated probabilities

+--------------------------------------------------------+

| Group | Prob | Obs_1 | Exp_1 | Obs_0 | Exp_0 | Total |

|-------+--------+-------+-------+-------+-------+-------|

| 9 | 0.0787 | 10 | 10.0 | 117 | 117.0 | 127 |

| 10 | 0.1111 | 1 | 1.0 | 8 | 8.0 | 9 |

+--------------------------------------------------------+

Number of observations = 136

Number of groups = 2

Hosmer–Lemeshow chi2(0) = 0.00

Prob > chi2 = .

Warning: There are only 2 distinct quantiles because of ties.

**Undefined
MEWS** Table collapsed on quantiles of estimated probabilities

+--------------------------------------------------------+

| Group | Prob | Obs_1 | Exp_1 | Obs_0 | Exp_0 | Total |

|-------+--------+-------+-------+-------+-------+-------|

| 7 | 0.1316 | 10 | 10.0 | 66 | 66.0 | 76 |

| 10 | 0.2400 | 6 | 6.0 | 19 | 19.0 | 25 |

+--------------------------------------------------------+

Number of observations = 101

Number of groups = 2

Hosmer–Lemeshow chi2(0) = 0.00

Prob > chi2 = .

Warning: There are only 2 distinct quantiles because of ties.

**NEWS**Table collapsed on quantiles of estimated probabilities

+--------------------------------------------------------+

| Group | Prob | Obs_1 | Exp_1 | Obs_0 | Exp_0 | Total |

|-------+--------+-------+-------+-------+-------+-------|

| 7 | 0.1053 | 8 | 8.0 | 68 | 68.0 | 76 |

| 10 | 0.3200 | 8 | 8.0 | 17 | 17.0 | 25 |

+--------------------------------------------------------+

Number of observations = 101

Number of groups = 2

Hosmer–Lemeshow chi2(0) = 0.00

Prob > chi2 = .

Warning: There are only 2 distinct quantiles because of ties.

**NEWS-2** Table collapsed on quantiles of estimated probabilities

+--------------------------------------------------------+

| Group | Prob | Obs_1 | Exp_1 | Obs_0 | Exp_0 | Total |

|-------+--------+-------+-------+-------+-------+-------|

| 6 | 0.1000 | 7 | 7.0 | 63 | 63.0 | 70 |

| 10 | 0.2903 | 9 | 9.0 | 22 | 22.0 | 31 |

+--------------------------------------------------------+

Number of observations = 101

Number of groups = 2

Hosmer–Lemeshow chi2(0) = 0.00

Prob > chi2 = .

Warning: There are only 2 distinct quantiles because of ties.

**qSOFA**

Table collapsed on quantiles of estimated probabilities

+--------------------------------------------------------+

| Group | Prob | Obs_1 | Exp_1 | Obs_0 | Exp_0 | Total |

|-------+--------+-------+-------+-------+-------+-------|

| 8 | 0.1059 | 9 | 9.0 | 76 | 76.0 | 85 |

| 10 | 0.4375 | 7 | 7.0 | 9 | 9.0 | 16 |

+--------------------------------------------------------+

Number of observations = 101

Number of groups = 2

Hosmer–Lemeshow chi2(0) = 0.00

Prob > chi2 = .

Warning: There are only 2 distinct quantiles because of ties.
